# INFOGEST Digestion Assay of Raw and Roasted Hazelnuts and Its Impact on Allergens and Their IgE Binding Activity

**DOI:** 10.3390/foods11182914

**Published:** 2022-09-19

**Authors:** Ivana Prodić, Katarina Smiljanić, Christoph Nagl, Barbara Ballmer-Weber, Karin Hoffmann-Sommergruber, Tanja Ćirković Veličković

**Affiliations:** 1 University of Belgrade—Faculty of Chemistry, Innovation Center Ltd., 11000 Belgrade, Serbia; 2 University of Belgrade—Faculty of Chemistry, Center of Excellence for Molecular Food Sciences & Department of Biochemistry, 11000 Belgrade, Serbia; 3 Department of Pathophysiology and Allergy Research, Medical University of Vienna, 1090 Vienna, Austria; 4 Allergy Unit, Department of Dermatology, University Hospital Zurich, 8091 Zurich, Switzerland; 5 Ghent University Global Campus, Incheon 406-840, Korea; 6 Ghent University, Faculty of Bioscience Engineering, 9000 Ghent, Belgium; 7 Serbian Academy of Sciences and Arts, 11000 Belgrade, Serbia

**Keywords:** Cor a 8, Cor a 9, gastric digestion, hazelnut allergens, IgE binding, lipid-protein interaction

## Abstract

Most of the food allergens sensitized via the gastrointestinal tract resist thermal treatments and digestion, particularly digestion by pepsin. Roasted hazelnuts are more commonly consumed than raw ones. Since no studies have characterized gastric digestion protein fragments of raw and roasted hazelnuts nor their IgE binding properties, we compared these aspects of raw and roasted hazelnuts’ gastric digesta obtained by INFOGEST protocol. Their electrophoretically resolved profiles were probed with hazelnut allergic patients’ sera in 1D and 2D immunoblots. Electrophoretic profiles demonstrated pepsin digestion of all hazelnut allergens to varying extents. While 2D immunoblots indicated that roasting slightly reduced allergenicity, IgE ELISA with the pool of sera showed a slight significant (10%) increase in IgE binding in both gastric digesta. Cor a 9 isolated from the raw and roasted hazelnuts, characterized by far and near CD, remained stable after roasting, with preserved IgE reactivity. Its immunoreactivity contribution by inhibitory ELISA was noticeable in raw and roasted hazelnut digesta; its activity was slightly stronger in the roasted preparations. Roasting has a visible impact on proteins; however, it did not affect overall IgE reactivity. Gastric digestion slightly increases the overall IgE reactivity in raw and roasted hazelnuts, and may therefore impact the profiles of allergens and their fragments available to interact with the immune system in the small intestine.

## 1. Introduction

Hazelnut allergy prevalence was understudied until recently, despite its clinical importance. Studies have shown that prevalence of hazelnut allergy depends on the patients’ age and their geographical location [1]. Cor a 9, the 11S globulin, is one of the major hazelnut allergens, inducing upon ingestion moderate to severe symptoms in individuals allergic to hazelnut. IgE reactivity to Cor a 9 represents a good predictor of food allergy to hazelnut, in contrast to Cor a 1 which is major allergen associated with birch-pollen-related hazelnut allergy [2]. Cor a 9 is a storage protein of high molecular weight that consists of two subunits, acidic (40 kDa) and basic (21 kDa). These subunits are connected by a disulfide bridge and form a hexameric structure [3]. It should be emphasized that the acidic chain of Cor a 9 contains IgE-binding epitopes [4]. Cor a 8 is also a major allergen, a non-specific lipid transfer protein (nsLTP) of low molecular weight (~9 kDa) which predominantly causes food allergic reactions in patients in the Mediterranean area [5].

Wigotzki et al., (2000) used 1D immunoblot with a defatted hazelnut extract probed with the sera of patients allergic to hazelnut, to show that the IgE-binding activity of the hazelnut allergens was preserved after conventional roasting (155 °C, 15 min), decreasing substantially after 15 min of roasting at 185 °C [6]. Protein extracts of various sources of raw and roasted hazelnuts (170 °C for 10 min), resolved via 1D sodium dodecyl sulphate polyacrylamide electrophoresis (1D SDS-PAGE) gel, showed minor differences between their corresponding matching profiles in terms of storage duration and extract preparation route, including Cor a 9 [7]. In the same study, IgE binding to total extract, and specific to Cor a 9, was expected to be higher in roasted hazelnut preparation than in raw, since a higher amount of Cor a 9 was determined in respective roasted preparations [7]. Meanwhile, Cor a 8 is known to be less stable when exposed to temperatures above 90 °C, probably due to the existence of a lipid-binding tunnel [8]. Therefore, gastric digestion stability is considered an important prerequisite for determining the allergenicity of food proteins [9]. So far, post-digestion allergenicity has been evaluated only for purified proteins from raw hazelnut [10,11], with digestion protocols based on extremely low pH, in the presence of high pepsin activity, not taking into account the effect of the food matrix [12]. Two studies in particular have considered the digestion fate of raw and roasted hazelnut allergens. The first was by Korte et al., (2017), using peptides of gastrointestinal digestion of raw hazelnut (INFOGEST protocol) quantified without labels, with their IgE epitopes determined in silico [13]. The second, by Di Stasio et al., (2020), used a comprehensive proteomic comparison of raw and roasted tree nuts (INFOGEST protocol) to investigate the effect of jejunum brush border enzymes [14]. However, neither of these studies considered the structure and allergenicity assessment of peptides obtained after in vitro gastric digestion. 

In this study, we applied the INFOGEST 1.0 protocol [15] on the raw and roasted hazelnuts, simulating physiological conditions that include the food matrix applicable to whole foods. INFOGEST protocol strongly recommends following consecutive digestion phases (oral, gastric, intestinal phase) without any defatting prior to digestion, to avoid neglecting the impact of allergen-protecting lipids and the food matrix in general. Our previous studies have developed and improved this approach [16,17]. In addition, it is critical to analyze the IgE binding potency of gastric digestion products of raw and roasted hazelnuts, because it has been shown that gastric digesta of raw and roasted peanuts differ in digestion fragments and allergenicity, which are central to duodenal sensitization processes [16,17,18]. Thus, this study aimed to characterize the structural and allergenic properties of the hazelnut allergens and their gastric digestion, using fragments from raw and roasted hazelnuts, focusing on Cor a 9, which was isolated and purified from the raw and roasted hazelnut extracts. The secondary and tertiary structures of the extracts were compared by circular dichroism spectroscopy (CD), and their allergenic properties by IgE ELISA test. In addition, the digestion pattern of Cor a 8 was monitored with specific antibodies raised against Cor a 8. Differences in overall IgE reactivity between different hazelnut digesta were captured by probing 2D immunoblots and by ELISA, with raw and roasted Cor a 9 as an inhibitor, using sera from patients allergic to hazelnut. The mass spectrometry results covered the basic region, masked by lipid-rich smears in 2D SDS-PAGE gels.

## 2. Materials and Methods

### 2.1. Materials 

α-Amylase from human saliva (EC 3.2.1.1; A0521-500 UN; Type IX-A, lyophilized powder 1000–3000 U·mg^−1^ protein) and porcine pepsin from gastric mucosa (EC 3.4.23.1; P6887-1G, lyophilized powder, 3200–4500 U·mg^−1^ protein) were purchased from Sigma–Aldrich (Saint-Louis, MO, USA). The enzyme activities were measured according to the assays detailed by Minekus et al., (2014) [15]. Chemicals for gel electrophoresis (Tris, glycine, CHAPS, urea, thiourea, dithiothreitol (DTT), acrylamide, bis-acrylamide, trichloroacetic acid (TCA), and Coomassie brilliant blue R-250 (CBB)) were purchased from Sigma-Aldrich. Ampholytes and immobilized pH gradient strips (IPG) were supplied by GE Healthcare (Uppsala, Sweden). All chemicals were analytical reagent grade, and mili-Q water (18 MΩ cm at 25 °C) (Millipore, Bedford, MA, USA) was used for all experiments. 

### 2.2. Patients’ Sera

Sera from nine patients with clinically relevant hazelnut allergy were included in this study. All sera had IgE specific to hazelnut extract. Out of these, a pool of seven sera was used for immunoblots, while a pool of five sera was used for ELISA experiments (Table S1). Written informed consent was obtained from donors prior to blood donation and their data were processed and stored according to the principles stated in the Declaration of Helsinki. 

### 2.3. Hazelnut 

Raw hazelnuts (*Corylus avellana*) were purchased from a local supplier. Hazelnut kernels were subjected to dry roasting at 140 °C for 40 min [19]. Using a coffee grinder (Bosch ErgoMixx 800 W Hand Blender, Munich, Germany), raw and roasted hazelnuts with skin were ground, separately, three times for 5 min to obtain particle size <1.5 mm, mimicking chewing during oral digestion. The obtained hazelnut flour was used for all experiments in this manuscript.

### 2.4. Simulated Oral and Gastric In Vitro Digestion Conditions 

Oral and gastric in vitro digestion of ground raw and roasted hazelnut samples were carried out according to INFOGEST 1.0 protocol [15]. The concentrations of electrolytes in stock solutions of simulated salivary fluid (SSF) and simulated gastric fluid (SGF) were prepared as proposed in the protocol. 

**Oral phase:** Solid ground raw (3 g) and roasted (2.85 g) hazelnuts were mixed with 2.4 mL 1X-SSF stock solution. Human salivary α-amylase (300 μL, 1500 U·mL^−1^ in 1X-SSF) was added to achieve 75 U·mL^−1^ in the final digestion mixture, followed by CaCl_2_ (300 μL, 15 mM in 1X-SSF) to achieve 0.75 mM in the final mixture. To reach the final volume of the digestion mixture, in the control and digestion samples of the roasted hazelnut, 150 μL of water aliquots were added to each sample. This leveling was applied due to water loss during roasting. Water loss was checked in triplicate, and was found to be ~5%. The reaction mixture was incubated for 2 min at 37 °C with agitation in the water bath (Aqua bath 96, Neomedica, Genesis Diagnostics Ltd., Littleport, England). All reagents were previously pre-warmed at 37 °C for 5 min. Controls without hazelnuts (food replaced by quartz sand) and controls without amylase (replaced with 1X-SSF) were also included. The gastric digestion phase (Dg) followed the oral phase.

**Gastric phase:** Complete oral phase material (6 mL) was mixed with 2 mL of 2X-SGF stock solution and 60 μL of CaCl_2_ (15 mM; in water) to achieve 75 µM in the final digestion mixture. Porcine pepsin (1 mL; 24,000 U·mL^−1^ in 2X-SGF) was added, to achieve 2000 U·mL^−1^. The pH of final mixture was adjusted to 3 with 1 M HCl; for raw hazelnut 200 μL and for roasted hazelnut 400 μL of 1 M HCl was added. Finally, water was added (2740 μL for raw hazelnut and 2540 μL for roasted) to make 12 mL of the final oral–gastric reaction mixture. The reaction mixture was incubated for 120 min at 37 °C with intense agitation (600 rpm) in a thermoshaker (Eppendorf^®^ New Brunswick™ Innova^®^ 42/42R Incubator Shaker, Hamburg, Germany). Control samples were run in parallel to the simulated digestion; pepsin control (3 g of sand instead of oral bolus were mixed with 2.7 mL of 1X-SSF stock solution and 300 μL CaCl_2_ (15 mM in 1X-SSF) at 0 (0′) and 120 (120′) min, and hazelnut control (Ct—oral bolus without enzymes, but with the addition of 1 mL 2X-SGF instead of pepsin solution). The final volumes of the control oral–gastric reaction mixtures were 12 mL. Digestion was stopped by the addition of 1 mL 1 M NaHCO_3_ to the final reaction mixture to obtain pH = 8. The final mixtures of digesta were centrifuged at 10,000× *g* for 20 min at 4 °C. Upper fat layers were carefully removed with a spoon then once more centrifuged at 10,000× *g* for 20 min at 4 °C. The liquid phase was separated from the solid material and immediately frozen at −20 °C; this liquid phase was used for all further analyses. 

### 2.5. SDS-PAGE Analyses

#### 2.5.1. One-Dimensional (1D) SDS-PAGE of Non-Defatted Liquid Gastric Digesta

Twenty μL of each non-defatted liquid gastric phase from the raw and roasted hazelnuts were resuspended in Laemmli sample buffer (reducing and non-reducing conditions). Then, 1D SDS-PAGE, with intentional overload, was carried out on 14% polyacrylamide gel according to Laemmli [20], and gels were stained with CBB.

Another normal load range, 1D SDS-PAGE, was run under reducing conditions with the same sample load as the 2D SDS-PAGE, applying 1.5 μL of sample per well (1.5 μL per 4 mm). 

#### 2.5.2. 1D SDS-PAGE with Defatted Liquid Gastric Digesta

The non-defatted liquid of the gastric phase (400 μL) was mixed with 400 μL of 20% of TCA (previously dissolved in 100% acetone). Samples were kept overnight at −20 °C, then centrifuged at 10,000× *g* for 20 min at 4 °C. The pellets were washed two times with 1 mL of 100% acetone. Dried TCA–acetone protein pellets from the liquid portion of the gastric-simulated digesta were resuspended in Laemmli sample buffer (reducing and non-reducing conditions).

#### 2.5.3. Densitometry with ImageQuant TL Version 8.1

Imaging and analyses of SDS-PAGE profiles and 1D immunoblots of thermally treated hazelnut digesta were performed using a laser biomolecular imager Typhoon FLA 7000 series (GE Healthcare, Uppsala, Sweden) and Image 1D Quant TL 8.1 software (GE Healthcare, Howard County, MA, USA). To quantify the intensity of CBB-stained protein bands, a non-calibrated mode pixel inverter function was chosen, with automatic-width peak (band) detection, a peak slope of 85–150, and level-5 noise reduction. The minimum height of peaks was detected at less than 4% of the highest peak in the sample, and a rolling ball (radius 500–1000) was used for the background subtraction. Thermo Scientific™ Pierce™ unstained protein molecular weight markers (7 µL), a mixture of seven native proteins (14.4 kDa to 116 kDa, final concentration 1 mg·mL^−1^), were applied in a collective mode to calibrate protein band volumes (volume = area × pixel intensity, seven marker bands, total volume 7 µg). The cubic spline model of the molecular-weight calibration curve was chosen as the best fitting model. Figures S2–S5 in Excel Report S2 (Supplementary Materials) provide a representative example of the entire process of digitalization (pixelization); IgE response in 1D immunoblots was expressed by calibrated volume in μg and normalized by calibrated volume of the respective protein bands (μg).

#### 2.5.4. Two-Dimensional (2D) SDS-PAGE of Non-Defatted Liquid Gastric Digesta

For 2D SDS-PAGE using 7 cm IPG strips, 30 μL of liquid gastric digesta and their controls were resuspended in 95 μL of slightly modified rehydration buffer (8.1 M Urea, 2.3 M Thiourea, 4.6% CHAPS, 0.5% IPG buffer 3–10NL, 50 mM DTT, and 0.002% bromophenol blue). The resulting sample (125 μL) actively rehydrated a 7 cm IPG strip (pH 3–10, nonlinear-NL, GE Healthcare, Uppsala, Sweden). Isoelectrofocusing was carried out on the Ettan IPGPhor3 system (GE Healthcare, Uppsala, Sweden) according to laboratory protocol optimized for lipid-rich samples (Table S2). Focused strips were reduced with DTT and alkylated with IAA, according to Apostolovic et al. (2016) [21]. The second dimension of migration was carried out on 14% polyacrylamide gels, and colloidal CBB staining was used for spot visualization [22]. 

### 2.6. Mass Spectrometry Analysis

#### 2.6.1. Sample Preparation for Nano Liquid Chromatography Coupled to Tandem Mass Spectrometry (nLC-MS/MS)

Vertical lipid-rich smears in the basic region were excised from 14% 2D SDS-PAGE gels with resolved gastric digesta and their controls from raw and roasted hazelnuts, as per Figure S1 (total of 18 samples). Samples were prepared for nLC-MS/MS analysis with trypsin digestion, as previously described by Shevchenko et al. (2006) [23]. A corresponding map of 2D gels is provided in Figure S1 (Supplementary Materials). The excised gel protein regions had their disulfide bridges reduced by 10 mM DTT, and cysteines were alkylated with 55 mM IAA. Samples were digested with proteomics-grade trypsin (Sigma-Aldrich, Taufkirchen, Germany) overnight at 37 °C. 

##### 2.6.2. nLC-ESI-MS/MS with Orbitrap Exploris 240

Proteins from gastric digesta and their respective controls of raw hazelnut were chromatographically separated using the Dionex Ultimate 3000 LC nano system (Thermo Scientific, Bremen, Germany). The mobile phase was A: 0.1% formic acid in water, or B: 0.1% formic acid in acetonitrile. Samples were chromatographically resolved using 5–70–95% B gradients throughout 23 min at a flow rate of 250 nL·min^−1^. Peptides were analyzed using Orbitrap Exploris 240 (Thermo Scientific, Bremen, Germany) with 60,000 resolution, in positive, data-dependent mode, with the 10 most intense precursors subjected to fragmentation by collision-induced dissociation.

#### 2.6.3. Identification of Hazelnut Allergens in the Basic Region by PEAKS Studio Xpro 

Hazelnut proteins in the basic region marked with red squares on the map (Figure S1) were identified using the PEAKS Xpro platform (Bioinformatics Solutions Inc., Waterloo, ON, Canada). Signature MS/MS spectra were searched using the PEAKS PTM algorithm, with a hybrid database consisting of UniProtKB hazelnut (Corylus [13450]) data with 2064 entries (http://www.uniprot.org/, accessed on 12 November 2021) and the common repository of adventitious proteins (cRAP) database (http://www.thegpm.org/crap/, accessed on 12 November 2021). Certain modifications were taken into account as variables; i.e., oxidation (Met) and deamidation (Gln, Asn), while carbamidomethylation (Cys) was set as fixed. Up to one missed cleavage with semi-specific cleavage at either end of a peptide was allowed. Mass tolerances were set to ±7 ppm for parent ions and ±0.02 Da for fragment ions. Protein filters were set to one unique + significant peptides, and protein score at least (−10 logP) 20. Peptide filters included the resulting score (−10 logP) as the lowest value for peptide, securing less than 1% of peptide sequence false-discovery rate, and 0% false-discovery rate at the protein level, with de novo ALC Score ≥50%. The details of all protein identifications generated by the PEAKS PTM algorithm are provided in Report S1, which is an integral part of the Supplementary Materials.

### 2.7. Purification of Cor a 9 from Raw and Roasted Hazelnuts

Raw and roasted ground hazelnut flours were used for the purification of Cor a 9 allergen. Cor a 9 was isolated, according to Rigby et al., (2008) with slight changes [24]. 

Flours from raw and roasted hazelnuts (16 g each) were stirred for 30 min at room temperature (RT) with *n*-hexane (100%, *w*/*v*, 1:6). After mixing hazelnut flour with *n*-hexane, each suspension was centrifuged in an ultra-centrifuge at 4 °C, 5000–7000× *g* for 10 min (Sorvall WX80, ThermoElectron Led GmbH, Langelselbold, Germany). This process of *n*-hexane defatting was repeated three times. The hazelnut flour was then allowed to dry in the air overnight, at RT, under the fume hood. The next day, hazelnut extracts were prepared. Dried flour (6.6 g) was mixed at 1:15 (*w*/*v*) with the extraction buffer comprised of Tris/HCl 20 mM pH 8.0, 2 M NaCl, 3% (*w*/*v*) polyvinylpolypyrrolidone (Sigma Aldrich), and protease inhibitor tablets (1 tablet/25 mL, Roche Complete Protease inhibitor tablet, 1:10 *w*/*v*), and mixed at 4 °C, for 3 h. The suspension was further centrifuged for 1 h at ultracentrifuge, at 4 °C, 40,000× *g*. The supernatant was removed and collected in one bottle (approximately 100 mL of extract), and filtered through a 0.2 µm cellulose acetate filter (Millipore, Darmstadt, Germany). Aliquots of the hazelnut protein extract were prepared (10 mL of extract into a 15 mL falcon tube). 

Aliquots of 10 mL were applied to the preparative size-exclusion column (HiPrep 26/60 Sephacryl S-200 HR, PerSeptive Biosystems, MA, USA) attached to an AKTA BASIC FPLC TM system (GE Biosciences, Buckinghamshire, UK). Samples were equilibrated in 20 mM Tris pH 8.0 containing 0.5 M NaCl. Proteins were eluted with the running buffer of 1.5 column volumes, and the obtained fractions were analyzed by 14% SDS-PAGE. The fractions containing proteins of higher molecular weight (e.g., Cor a 9 and Cor a 11) were pooled and used for subsequent Concanavalin A (Con A) affinity chromatography with Con A Sepharose (self-packed column, 5 mL volume, Thermofisher, Little Chalfont, UK), equilibrated in 20 mM Tris pH 7.4 containing 0.5 M NaCl. After sample loading, unbound proteins were collected containing only Cor a 9. Bound proteins were eluted with an extraction buffer containing 0.4 M methyl α-D-mannopyranoside. The flow-through was analyzed by 14% SDS-PAGE. Protein concentration was determined using a BCA assay (Thermo Fisher Scientific Inc., Bremen, Germany).

#### 2.7.1. CD Spectroscopy of Cor a 9 Purified from Raw and Roasted Hazelnuts

CD spectra were recorded on a JASCO J-815 spectropolarimeter (JASCO, Tokyo, Japan) for both proteins, obtained from raw and roasted hazelnuts. FAR-UV spectra were recorded at a concentration of 0.5 mg·mL^−1^ in 20 mM Tris buffer (pH 7.4) containing 0.5 M NaCl. The spectra were collected over the wavelength range of 195–260 nm. The secondary structure composition was calculated using the CDPro server (program: Selcon). Near-UV spectra were recorded as separate experiment at a concentration of 1 mg·mL^−1^ in 20 mM Tris buffer (pH 7.4) containing 0.5 M NaCl. The spectra were collected in near range over the wavelength range 260–320 nm. A 1 cm cuvette was used. For both far- and near-UV spectra, the scan speed was 50 nm/min, data interval 0.5 nm, and bandwidth 1.0 nm. Each sample was recorded in quadruplicate, and the results were averaged. Results were expressed as average residue molar ellipticity.

#### 2.7.2. IgE ELISA with Raw and Roasted Cor a 9 with Patients’ Sera Allergic to Hazelnut 

An IgE ELISA test was performed with Cor a 9 obtained from raw and roasted hazelnuts. Purified Cor a 9 was diluted in 50 mM Na_2_CO_3_ buffer, pH 9.6. Microtitre plates (MaxiSorb Immunoplate, Roskilde, Denmark) were coated with 100 µL of (2 µg·mL^−1^) of the protein. The plate was washed three times with Tris-buffered saline (TBS) pH 7.4 containing 0.05% of Tween 20 (*w*/*v*), and blocked with 200 μL/well 3% BSA for 1 h at RT in TBS containing 0.05% of Tween 20 (TTBS). Five patients’ sera (Table S1) were added, and the incubation was performed with dilution (1:10) in TTBS  +  1% BSA overnight at 4 °C. IgE binding was detected by incubation with 1:1000 diluted alkaline phosphatase (AP)-conjugated mouse anti-human IgE antibodies (BD BioSciences, Heidelberg, Germany) for 2 h at RT, and color was developed with p-nitrophenyl phosphate substrate tablets (pNPP, Sigma-Aldrich Co. LLC, Germany). Optical density (OD) was measured at 405 nm, and the mean value GraphPad Prism 7.0 software (La Jolla, CA, USA) was used. 

### 2.8. IgE Binding Properties of Hazelnut Gastric Digesta

#### 2.8.1. 1D and 2D Immunoblots of Non-Defatted Hazelnut Gastric Digesta 

Proteins were separated on 2D SDS-PAGE and transferred onto nitrocellulose (NC) membranes with 0.2 µm pore size (Bio-Rad, Solna, Sweden) for 50 min, 2 mA/cm^2^. Ponceau S staining was applied to verify the success of the transfer. The membranes were blocked with 2% BSA in TBS pH 7.4 containing 0.05% (*v*/*v*) Tween-20, for 2 h at RT. Subsequently, the membrane was incubated overnight at 4 °C with a 1:10 diluted sera pool from patients with proven hazelnut allergy in TTBS with 0.5% BSA. The pool consisted of sera from seven hazelnut-sensitized patients. The secondary antibody, AP-labelled anti-human IgE produced in goats (BD BioSciences, Heidelberg, Germany), was diluted 1:1000 in 0.5% BSA dissolved in TTBS, and incubated for 2 h at RT. The binding patterns were visualized with a substrate solution consisting of 1.5 mg 5-Bromo-4-chloro-3-indolyl phosphate and 3 mg nitroblue tetrazolium in 10 mL of 100 mM Tris, containing 150 mM NaCl and 5 mM MgCl_2_, pH 9.6. Two control strips were included, one without an IgE antibody and the second with a non-allergic patient. Raw control hazelnut sample was western blotted on the control strips used in the 1D immunoblot. 

#### 2.8.2. Inhibitory ELISA of Hazelnut Gastric Digesta with Cor a 9 as an Inhibitor

The IgE binding properties of hazelnut gastric digesta purified from raw and roasted hazelnuts were analyzed using inhibitory ELISA with Cor a 9 as an inhibitor. The liquid phase of non-defatted gastric digesta and their controls were used to coat microtitre plates (96 wells, MaxiSorb Immunoplate, Roskilde, Denmark) with 100 μL per well, incubated overnight at 4 °C in coating buffer (25 mM or 50 mM Na_2_CO_3_ buffer, pH 9.6). The remaining binding sites were blocked with 200 µL/well of 3% BSA in TTBS for 1 h at RT. A sera pool from five patients with hazelnut allergy (Table S1) was made following the EMEA Note for Guidance on Allergen Products (EMEA/CHMP/BWP/304831/2007). Pure Cor a 9 protein was diluted with 0.5% BSA in TTBS to reach final concentrations of 50 µg·mL^−1^, 10 µg·mL^−1^, 1 µg·mL^−1^, 0.1 µg·mL^−1^, and 0.01 µg·mL^−1^. Samples were pre-incubated 1:1 with the pool of sera (final dilution of sera pool was 50-fold in blocking buffer) for 1 h at 37 °C before plate incubation for another 1 h at 37 °C (total 2 h of incubation). Detection of IgE binding was performed with 100 µL/well of AP-conjugated anti-human IgE (BD BioSciences, Heidelberg, Germany) diluted 1:1000 in 0.5% BSA in TTBS for 1 h at RT. Finally, staining was performed with pNPP tablets (Sigma-Aldrich Co. LLC, Germany) dissolved in a ready-to-use Tris-buffered solution (Sigma-Aldrich Co. LLC, Germany). Inhibition of IgE binding was calculated as [(OD_no inhibitor_ − OD_inhibitor_)/OD _no inhibitor_] × 100. The results were analyzed in GraphPad Prism 7.0. 

### 2.9. 1D and 2D Western Blot of Non-Defatted Gastric Liquid Digesta Probed with Anti-Cor a 8 Antibody

Non-defatted liquid digesta of raw and roasted hazelnuts were resolved by 2D SDS-PAGE, as described in Section 2.5.4. Proteins were transferred onto NC membranes. Blocking and washing steps were performed as described in Section 2.8.1. Subsequently, the membrane was incubated overnight at 4 °C with a specific antibody raised in rabbit against Cor a 8, 1:10,000 diluted in 0.5% BSA dissolved in TTBS. The secondary antibody, goat anti-rabbit, labeled with AP, was diluted at 1:750 in 0.5% BSA in TTBS and incubated for 2 h at RT. The binding patterns were visualized as in Section 2.8.1. Incubation with the substrate was no longer than 5 min.

### 2.10. Statistical Analyses

Descriptive statistics, including unpaired two-tail *t*-test and one-way ANOVA results, were employed to highlight significant differences when examining raw and roasted Cor a 9 protein concentration with the BCA method, and when examining IgE responses between control and digesta pairs with IgE and inhibitory ELISA, at *p* < 0.05, undertaken by GraphPad Prism 7.0 software. All experiments were repeated at least twice, except the mass spectrometry identifications that were carried out once. 

## 3. Results & Discussion

Food allergen resistance to digestion is an essential factor that contributes to allergenicity; we aimed to examine the outcome of in vitro gastric digestion of hazelnut based on INFOGEST protocol [15], and whether the roasting process affects gastric digestion pattern and resistance of the main hazelnut allergen, Cor a 9. These tasks were not easy to complete, since in vitro gastric digestion of hazelnut as a whole food is challenging for analyses due to the presence of lipids, sugars, phenols, and other biomolecules, especially after thermal treatment such as dry roasting [17]. IgE reactivity of the non-defatted hazelnut digesta was compared by 1D and 2D immunoblotting and ELISA, using either individual or pooled sera from hazelnut allergic patients. From the basic regions of 2D SDS-PAGE gels, 18 spots were subjected to bottom-up tandem mass spectrometry to confirm the protein identity hidden behind substantial vertical lipid smearing. Cor a 8 allergen presence was further confirmed by specific antibodies in 2D immunoblots. Closer examination of the structural changes of pure Cor a 9 imposed by roasting of hazelnut was undertaken, including its inhibitory IgE potential against raw and roasted hazelnut matrices. 

Studying the efficiency of protein extraction during in vitro gastric food digestion that has been thermally treated requires proper control of the digestion process, e.g., complete in vitro simulating digestion mixtures but without digestive enzymes [17]. Extraction during digestion represents a key element in liberating proteins from a fat-rich texture, e.g., from tree nuts. The roasting of peanuts cross-links and oligomerizes their proteins, thus impairing extractability in gastric digestion compared to raw variants [17]. Under acidic conditions, representing an unfavorable environment for peanut protein extraction, the estimated amount of extracted proteins was up to 30% of the theoretical maximum [16]. In the case of hazelnut, from 3 g of paste, the maximum theoretical yield is limited by the extent of protein content, which can be up to 15% of hazelnut mass (up to 450 mg of protein in solution). Upon simulated oral–gastric digestion, only up to 10% of the theoretical maximum of protein extraction was attained in the raw and roasted controls for gastric digestion, as shown by the densitometry measurements (Report S2 as Excel file, Supplementary Materials). This result is in agreement with the findings of Angelis et al. (2018), where extraction using different neutral and slightly basic pH buffers was reported [25]. In addition, many intact proteins remaining in the food gastric bolus and reaching intestinal mucosa can potentially elicit allergic reactions upon favorable extraction in basic conditions [16,17].

### 3.1. 1D SDS-PAGE and 1D Immunoblots of Raw and Roasted Gastric Hazelnut Digesta and Their Controls

**Figure 1:**
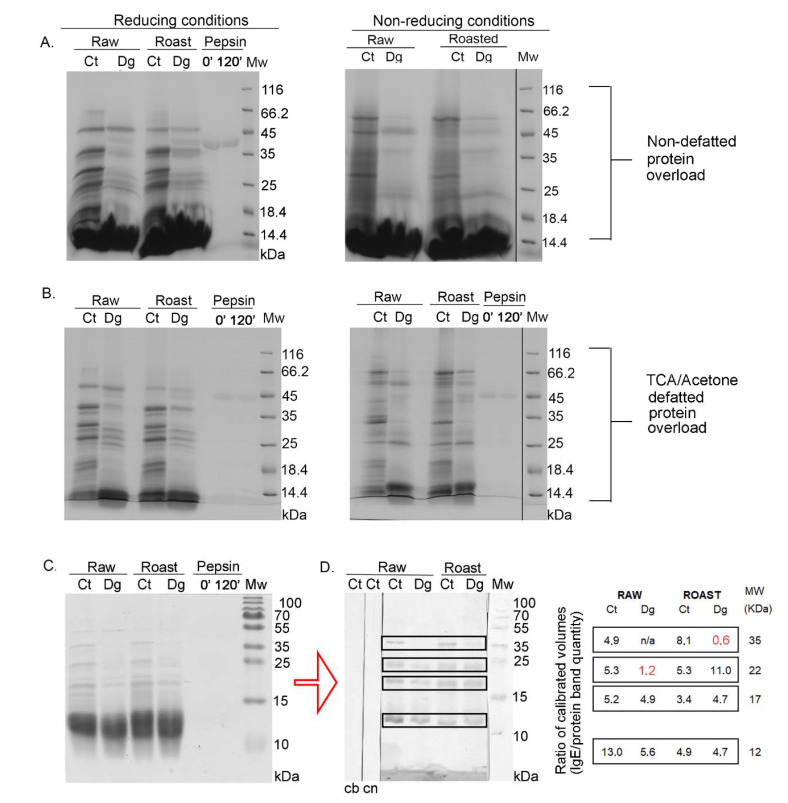
1D SDS-PAGE and 1D immunoblot of raw and roasted hazelnut digesta and their controls. (**A**) 1D SDS-PAGE, reducing condition of overloaded protein profiles from non-defatted raw and roasted gastric hazelnut digesta and their respective controls. (**B**) 1D SDS-PAGE, reducing (left) and non-reducing conditions (right), for overloaded protein profiles of raw and roasted gastric hazelnut digesta and their controls, which were defatted with TCA–acetone treatment. (**C**) 1D SDS-PAGE, reducing condition of normal load range of raw and roasted gastric hazelnut digesta and their controls, also used for 1D Western blots. (**D**) 1D Western blot of non-defatted raw and roasted gastric hazelnut digesta and their controls, probed with the pool of patients’ sera (left panel). Bound IgE was detected by rabbit anti-IgE antibody. SDS-PAGE was performed under reducing conditions. Right panel: Densitometry-quantified IgE-reactive binding, normalized with the quantity of proteins in respective gel bands (red text denotes values that have not been normalized due to absent gel band after densitometric detection). Ct, control sample (non-digested, e.g., without pepsin) after 120 min; Dg, gastric phase at 120 min; 0′—pepsin control at the start of the digestion; 120′—pepsin control at the end of digestion (120 min); cb, control of secondary antibody; cn, control with non-allergic patient serum; Mw, molecular weight markers; kDa, Kilo Daltons.

Readily-extractable proteins, released during extraction with simulated salivary and gastric fluids into the liquid phase, underwent pepsin digestion. The digestibility patterns and IgE immunoreactivity of proteins from raw and roasted milled hazelnuts are presented in Figure 1. In a whole food digestion experiment, it is desirable to use protein overloads in SDS-PAGE [16,17] to observe the different isoforms of the higher molecular mass proteins and allergens; oligomers possibly induced by food thermal treatment and larger digestion-resistant fragments may otherwise remain unnoticed with a normal protein load. Due to heavy post-translational processing of the hazelnut allergens, especially Cor a 9 which is a counterpart to the peanut allergen Ara h 3, we analyzed non-reducing gels in parallel to be able to observe full-length proteins with preserved disulfide bridges (Figure 1A,B, right panels). In addition, we were able to assess the extent of oligomerization supported by the involvement of disulfide bridges. In Figure 1A, right panel, the likely presence of the full-length Cor a 9 allergen [26,27] can be observed in all four lanes, strongest in the controls, with a slightly higher abundance in the roasted digesta compared with its raw counterpart. In addition, most of the smear noticed in the controls for the non-reducing gel had disappeared in the reducing gel, indicating that most of these oligomers were supported by disulfide bridges (Figure 1A). 

Cor a 11, a monomeric allergen seen as a band at ~48 kDa [27] in controls, was better extracted in an acidic environment in the raw preparations compared with roasted, and more efficiently digested by pepsin in roasted than in raw digesta, probably due to a higher enzyme–substrate ratio (Figure 1A). Prominent bands around 40 kDa and 25 kDa in controls (Figure 1A) corresponded to the acidic and basic subunit of Cor a 9, respectively. However, in digesta, two discrete bands around 25 kDa were noticed, probably corresponding to the basic subunit of Cor a 9. In controls, an intensive band around 30 kDa disappeared in the digesta, and most likely the digested products of this band became fragments in areas much lower than 30 kDa. The smear of lipids was more significant in the raw and roasted digesta than in their controls, and this can be explained by the release of lipids belonging to non-specific lipid-transfer proteins due to pepsin digestion, as well as from other lipid reservoirs by indirect pepsin action [28]. Pepsin quantity was the same in both lanes, as in Figure 1A’s left panel, meaning that pepsin was stable under the conditions specified in the study. Protein bands migrating below 17 kDa, in controls and digesta for both preparations corresponded to Cor a 8 and Cor a 14 [27]. However, in non-defatted sample modes, they could not be visualized due to large smears from lipids, masking that region (Figure 1A). 

To analyze the effects of digestion using the SDS-PAGE approach without lipids, liquid phases of gastric digesta and their respective controls were defatted with TCA–acetone and then electrophoretically resolved following the protocol described by Prodic et al., (2019) [17]. Figure 1B represents the protein overload defatted with TCA–acetone, and clear visualization was enabled of the resulting digestion pattern in the area below 20 kDa. In addition, with non-reducing gels, increased aggregation of hazelnut proteins was observed in the higher mass range after thermal processing, especially in the roasted control (Figure 1A,B, right panels). Similar observations have previously been made in the case of boiled, fried, and roasted peanuts [17,29]. Due to the pepsin action in the digesta of both preparations, disappearance of all well-defined protein bands was observed (Figure 1B). The excessive accumulation of peptides at l4.4 kDa bands was visible in both digesta and in the control of the roasted sample. However, caution must be applied when commenting on these results, because of the nature of the protein–peptide precipitation and defatting by the TCA–acetone method; also, it is not 100% quantitative, even when employing overnight precipitation at −20 °C [16,17,30]. In the case of hazelnut, after TCA–acetone precipitation and defatting, proteins remained detectable at their expected masses (Figure 1B); however, the loss of protein after defatting was lower compared with the peanuts [17]. However, the cause of such a result can only be speculated since no studies have been published on lipid-loss profiling during defatting.

Overall, when comparing raw and roasted digesta and their respective controls, under all conditions, it seems that roasting affected oral–gastric extraction and consequently also digestion, in terms of creating slightly different electrophoretic profiles. However, no effects on impairing or facilitating the overall extraction process were observed in an acidic environment (Report S2, densitometry assessment), the same as for the raw and roasted peanuts [17].

### 3.2. 1D Immunoblot of Non-Defatted Gastric Digesta of Raw and Roasted Hazelnuts and Their Controls

Figure 1C shows the stained gel of non-defatted hazelnut preparations with normal loads. Its unstained duplicate was used for western blotting and for subsequent 1D immunoblotting with a pool of sera from patients allergic to hazelnut (Figure 1D, left panel). The densitometric quantification of IgE responses, normalized against the quantity of proteins in the respective 1D SDS-PAGE gel bands (Figure 1C), is shown in Figure 1D, right panel. The most noticeable difference was in the lower mass range (~12 kDa), where a response more than twice as strong was observed in the raw control sample (Figure 1D, Report S2). Interestingly, only in the ~12 kDa gel region of the raw control, Cor a 14 was identified, alongside Cor a 8, fragments of Cor a 9, and Cor a 11 allergens (Report S1). Cor a 14 sensitization is known to be a risk marker for severe hazelnut allergy in children, as with Cor a 9 [2]. The other prominent difference observed was related to IgE binding corresponding to the gel position of the acidic subunit of Cor a 9 (band at ~40 kDa); while it was absent from both digesta according to the densitometry results (Report S2), which was most likely due to pepsin digestion, an IgE binding was detected in the roasted digesta and is labeled with red color to denote the original calibrated value of IgE response without protein normalization (Figure 1D, right panel). Differences were noted in the region’s pattern at the position of the basic subunit of Cor a 9 (~21 kDa), which was strongest in the roasted digesta while a faint response was recorded in the raw digesta (Figure 1D). Nitride et al., (2013) [31] showed that the Cor a 9 alkaline subunit as well as its acidic counterpart was also IgE-reactive, the former having been previously presented solely responsible for the predominant IgE reactivity [4]. In addition, IgE-reactive bands at 17 kDa were noticed in all preparations, most probably representing IgE reactivity to Cor a 12 (Figure 1D). No IgE binding was detected when using the serum of a non-allergic patient. 

When overall IgE binding was observed, IgE patterns of raw and roasted hazelnut controls were similar. Roasting of hazelnut affected its IgE response, leading to a slight increase in the gel region of the acidic Cor a 9, and an opposite decreasing trend in the region of 12 kDa. Gastric digestion decreased IgE reactivity in the raw hazelnut digesta, while in roasted hazelnut digesta, it brought almost no net change. This may point to a potential protective role for roasting, leading to the preservation of proteins’ IgE-binding capabilities. Namely, the roasting of food can cause a Maillard reaction that may affect the allergenicity of proteins by disrupting the conformational epitope, disclosing the hidden epitope, masking the linear epitope, and/or forming a new epitope [32]. Maleki et al. (2000) showed in the case of peanuts that the formation of novel epitopes occurred after roasting, even when performing experiments on pure proteins [33].

Allergenicity of hazelnut proteins was reduced after 40 min of roasting at 140 °C, as revealed by double-blind, placebo-controlled food challenge (DBPCFC), skin prick test, histamine release, and enzyme allergosorbent test (EAST), especially in the patient group that was sensitized to birch-pollen-related hazelnut allergens [19]. The explanation at the molecular level is yet to be delivered; however, EAST inhibition experiments have shown that both patient groups, in a negative and a positive DBPCFC for roasted hazelnut, recognize the same raw and roasted allergens with equal potency [19]. 

Concerning resistance to oral–gastric digestion, hazelnut allergens were at least partially resistant to oral–gastric digestion according to our results (Figure 1), and therefore able to sensitize the mucosal immune system. Vieths et al. (1999) [9], working on raw hazelnut, demonstrated according to 1D SDS-PAGE that all proteins of higher molecular masses (>14.4 kDa) underwent pepsinolysis, which is not the case for our findings. This discrepancy in results is probably due to different applications of gastric digestion protocols. Contrary to our results, in that study it was shown that IgE reactivity to hazelnut in all patients’ sera (four patients) was almost completely diminished due to pepsin digestion [9]. 

### 3.3. 2D SDS-PAGE and 2D Immunoblots of Non-Defatted Gastric Digesta

**Figure 2:**
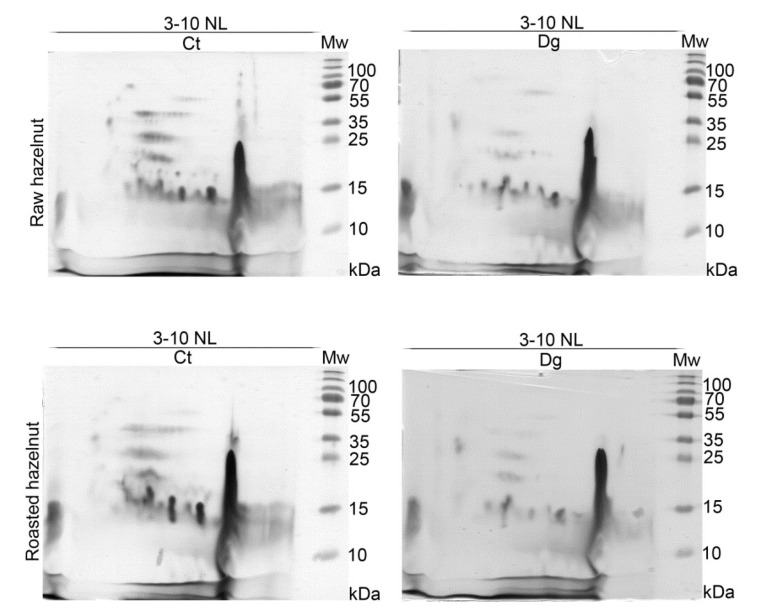
2D SDS-PAGE (14%) in reducing conditions with normal load range of non-defatted raw and roasted hazelnut digesta and their respective controls; Ct—control sample, Dg—gastric phase; Mw—molecular weight markers; kDa—Kilo Daltons; 3–10 NL—nonlinear pH range 3–10.

2D profiles of raw and roasted controls and their gastric digesta revealed rather similar proteomic profiles (Figure 2). Differences between controls and their respective digesta were observed, with equal overall extents of pepsin digestion, as revealed by 2D SDS-PAGE gels based on the same volume of digesta applied per strip. However, we assume that Cor a 11 (48 kDa) did not survive in the roasted digesta (Figure 2, Report S1). An acid subunit of Cor a 9 (40 kDa) was fully and partly proteolyzed in raw and roasted samples, respectively, which was in accordance with 1D SDS-PAGE results. In all four 2D gels, a very intense lipid-like vertical smear was noticed around pH 9. While for the majority of other spots in the acidic and neutral regions of the 2D maps, we have relied on the identifications from literature data [13,14], among other proteins in this specific region we identified Cor a 8, 9, 11, 12, and 14 allergens and their fragments (Report S1). 

**Figure 3:**
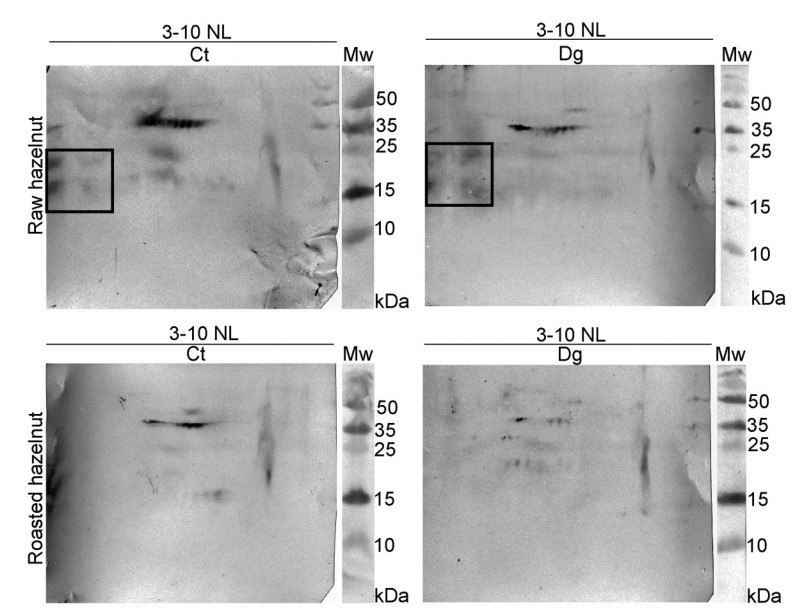
2D immunoblots probed with the pool of hazelnut-allergic patients’ sera on resolved non-defatted raw and roasted hazelnut gastric digesta and their respective controls in reducing conditions; Ct—control sample, Dg—gastric phase; Mw—molecular weight markers; kDa—Kilo Daltons; 3–10 NL—nonlinear pH range 3–10.

The overall 2D immunoblot outlook map shows that the raw hazelnut control had the highest IgE binding, while IgE reactivity in the roasted digesta was the weakest (Figure 3). Faint IgE immunoreactivity was detected in the raw preparations, assumed to belong to the 48 kDa-glycoprotein, Cor a 11, with the fainter version in the respective digesta. IgE binding induced by an acidic subunit of Cor a 9 was prominent in both controls. An obvious difference between raw and roasted hazelnut preparations was observed in the acidic region, where four IgE reactive spots around 15–25 kDa were found only in raw control and digesta, partially resisting pepsin digestion. This probably includes fragments of Cor a 9 formed during natural proteolysis of Cor a 9, similarly observed for 11S globulin Ara h 3 [34]. The negative control for 1D immunoblotting was conducted on raw control sample strips and with healthy subjects’ serum; the immunoblot showed no visible signals of IgE reactivity (Figure 1D, cn lane).

The basic chain of 11S globulin-like protein (Cor a 9) could easily overlap with a trace of nsLTP (Cor a 8). Therefore, proteomic experiments were conducted to elucidate the nature of this trailing IgE immunoreactivity around pH 9. The presence of Cor a 8, Cor a 9, and Cor a 14 was determined in the control of raw sample, while in its digesta, besides Cor a 8 and Cor a 9, Cor a 11 and Cor a 12 were also found in the low-mass gel region (10–15 kDa, Figure S1, Report S1). These findings are probably the result of pepsin digestion of these allergens, and their fragments’ migration into the corresponding basic gel region (Figure S1, Table S3, Report S1). In the basic smear region of the roasted hazelnut, only Cor a 8 [35] at different mass positions was identified in control and gastric digesta samples (Figure S1, Table S3, Report S1). 

### 3.4. Roasting Induced Slight Structural Changes with No Overall Effect on IgE Binding Capacities of Cor a 9 Purified from Raw and Roasted Hazelnuts

**Figure 4:**
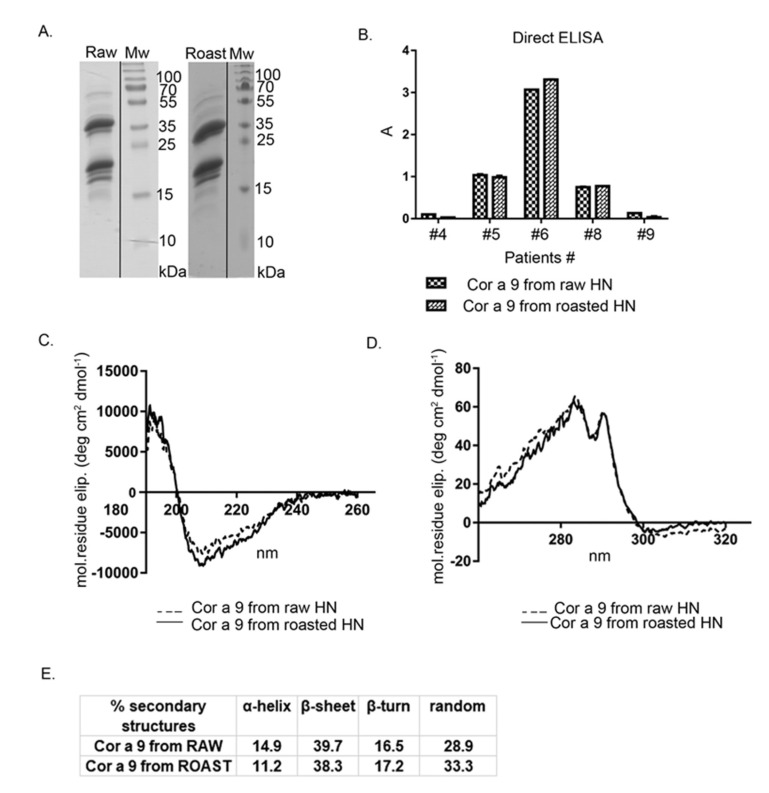
(**A**) 1D SDS-PAGE (14%) profile of Cor a 9 obtained from raw and roasted hazelnuts, isolated under the same reducing experimental conditions. Mw—molecular weight markers; (**B**) comparison of FAR-UV CD spectra of purified Cor a 9 obtained from raw and roasted hazelnuts; (**C**) comparison of the secondary structure of purified Cor a 9 obtained from raw and roasted hazelnuts; (**D**) comparison of Near-UV CD spectra of purified Cor a 9 obtained from raw and roasted hazelnuts; (**E**) IgE binding from individual sera of five hazelnut-allergic patients, with the raw and roasted hazelnuts, as revealed by IgE ELISA. A is the absorbance at 405 nm; HN—hazelnut.

Cor a 9, one of the most abundant hazelnut 11S seed storage globulins, was purified by gel permeation chromatography on Sephacryl S-200, followed by a purification step on the column of Con A Sepharose-4B. After the gel chromatography step, Cor a 9 and Cor a 11 were co-eluted. Cor a 11, a glycosylated allergen, bound to the Con A column, unlike Cor a 9, non-glycosylated protein. This two-step, relatively fast chromatographic approach obtained high-purity Cor a 9 preparations from previously defatted raw and roasted hazelnut flours (Figure 4A). The Cor a 9 profiles shown in Figure 4A show the typical features of the 11S globulin subunit structure, also described in peanut and pea [35,36]. At first glance, the electrophoretic profiles of Cor a 9 isolated from raw and roasted hazelnuts revealed no difference caused by the applied thermal treatment; both profiles were almost identical, with multiple bands on the SDS-PAGE (Figure 4A). 

An intense protein band at ~40 kDa, with an accompanying band at 34 kDa, corresponded to an acidic subunit, while bands between 20–23 kDa were associated with basic polypeptide chains of the Cor a 9 protein (Figure 4A). This is in agreement with the electrophoretic band patterns of Cor a 9 purified with different buffer systems [25,26]. Up to three minor groups of light polypeptides were also detected, at masses of 19, 18, and 15 kDa. These low-mass peptides probably represent the products of partial hydrolysis of Cor a 9 [24]. The protein profile of Cor a 9 obtained from roasted hazelnut extract possessed one additional faint band around 15 kDa, absent in the corresponding area of the raw hazelnut SDS-PAGE profile (Figure 4A). Only a few papers have reported on the evaluation of thermal processing effects on hazelnut proteins, and most considered Cor a 9 as thermostable [7,37]. 

Circular dichroism spectroscopy was applied to assess whether roasting treatment influenced Cor a 9 folding, compared with Cor a 9 obtained from the raw hazelnut. According to our preliminary immunochemical studies, purified Cor a 9 from roasted hazelnut retained its immunological properties in terms of IgE binding, determined by IgE ELISA with sera of hazelnut allergic patients. 

Far-UV CD spectra (Figure 4B) of Cor a 9 obtained from raw and roasted hazelnuts were characteristic of seed storage globulins, rich in β-sheet structures [38]. In our study, the Cor a 9 spectrum showed a positive maximum around 195 nm, similar to 11S globulin from soybean and glycinin, and a negative minimum between 208–220 nm [39]. Comparison of far-UV CD spectra between raw and roasted Cor a 9 indicated minor changes in secondary structures, caused by roasting. A slight shift of peak towards lower wavelengths was noticeable at 208 nm in the case of Cor a 9 obtained from roasted hazelnut (Figure 4B). Calculated α-helix, β-sheet, β-turn, and random coil values are listed in Figure 4C. Hazelnut roasting resulted in minor alterations to secondary structures, reflected in a slight decrease in α-helix content and a parallel increase in random coil content. However, these changes in secondary structure were not significant, suggesting that the secondary structure of this protein is quite stable. The near-UV CD (Figure 4D) spectrum of the Cor a 9 obtained from raw and roasted hazelnuts displayed peaks at about 293 and 285 nm. The near-UV CD spectra showed changes in the tertiary structure of the protein; according to our results, there were minimal changes in the structures of aromatic amino acids on the protein Cor a 9 isolated from raw and roasted hazelnuts. This result indicates that thermal changes had a minimal effect on the tertiary structure of the Cor a 9 allergen. 

Dyer et al., (2018) reported that the thermal processing of Ara h 3 in peanuts resulted in increased IgE binding for every serum tested, compared with the raw peanut counterpart [40]. However, the CD data tended to be very similar, and the percentages of the predicted secondary structural elements did not change significantly after heating, which indicated that the roasting process did not substantially influence Ara h 3 [40]. Results reported in that paper for the same type of protein 11S globulin, resemble the results of this current study. 

Since Cor a 9 remained stable after roasting [7,41], an examination of IgE binding was conducted (Figure 4E). As expected from CD spectral analyses, raw and roasted Cor a 9 proteins were recognized by specific IgE antibodies derived from allergic patients’ sera, with almost no changes in intensity. In our study, Cor a 9 was recognized by sera of three patients out of five, similarly for both preparations (Figure 4E). However, Cor a 9 from raw hazelnut was recognized by sera of 86% of patients in the study by Beyer et al. (2002) [4]. It is important to emphasize that conformational and linear epitopes are relevant in this experiment. Based on similar IgE reactivity profiles of raw and roasted Cor a 9, we assume that these were not distorted by roasting. It has been suggested that the allergenicity of Cor a 9 is predominantly related to conformational and not to linear epitopes [42]. 

### 3.5. Assessment of IgE Binding Potency after Simulated Gastric Digestion of Raw and Roasted Hazelnuts

**Figure 5:**
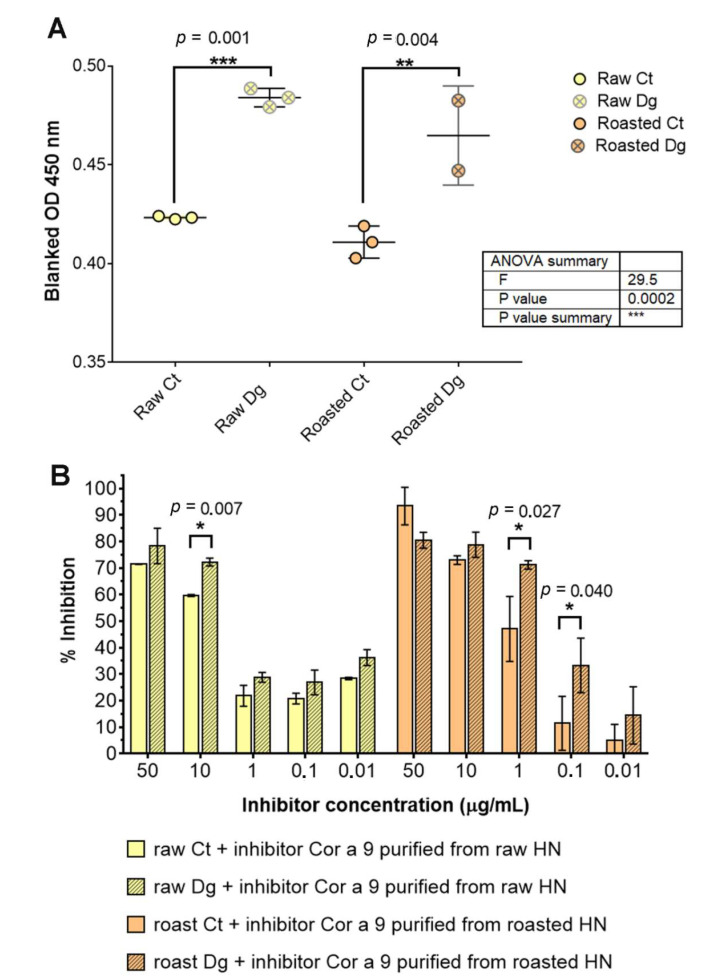
IgE and inhibitory ELISA tests with the pool of hazelnut-allergic patients’ sera: (**A**) IgE ELISA was performed in triplicate with the pool of sera of designated patients allergic to hazelnut, as specified in Table S1. One-way ANOVA test with Tukey’s multiple comparison post-test was applied to search for significant differences at an alpha value less than 0.05. (**B**) Inhibition of specific IgE binding by Cor a 9 obtained from raw and roasted hazelnuts. Unpaired two-tail t-testing was used with corrected multiple comparisons by the Holm–Sidak method with alpha set to 0.05. * significant difference at 0.005 < *p* < 0.05, ** significant difference at 0.0005 < *p* < 0.005, *** significant difference at *p* < 0.0005, Ct—control sample, Dg—gastric phase, HN—hazelnut.

To compare the IgE-binding potency of raw and roasted hazelnut protein extracts, IgE ELISA and inhibitory ELISA tests were applied (Figure 5) using a pool of sera from five patients allergic to hazelnut. In these assays, the same volumes of raw and roasted digesta/controls (100 μL) were added to the plate. Concentrations estimated by densitometry were within the recommended range of 1–2 mg·mL^−1^ for protein extracts, and corresponding resulting protein amounts were applied per well in the ratio raw Ct: raw Dg: roast Ct: roast Dg = 1:0.6:1.1:0.7 (Report S2). Looking only at the data of the IgE ELISA in Figure 5A, it can be observed that the IgE reactivity of each gastric digesta was significantly higher (~10%) compared with their controls. This result suggests that roasting per se did not influence IgE reactivity, in contrast to gastric digestion itself. This could be due to the increased number of small peptides created during the digestion that continued to retain their allergenic properties, as shown in the study by Prodic et al. (2018) [16].

Cor a 9, isolated from the raw and roasted hazelnuts, was used as inhibitor in a concentration-increasing manner in the inhibitory ELISA (Figure 5B). The results showed that pure raw Cor a 9 inhibited raw gastric digesta more strongly than its control, at each concentration applied, with the greatest difference being 15% at 10 μg·mL^−1^ (Figure 5). In the case of roasted hazelnut with the roasted Cor a 9 as an inhibitor, the situation was replicated except at the 50 mg·mL^−1^ point. Observing all samples, we can assume that Cor a 9 isolated from the roasted hazelnut was more potent than its raw counterpart in inhibiting the IgE binding of the roasted digesta. It remains unclear whether this effect is genuine in spite of unequal coating amounts (especially between controls and digesta), because coating saturation plateau was reached in all four preparations, or if it is false due to unequal coating amounts (i.e., saturation plateau was not reached in digesta). It is logical to suspect that the effect observed was due to decreased Cor a 9 concentration in the roasted samples (Figure 5B). 

Wigotzki et al. (2000) published a study where their subjects of interest were different hazelnut varieties [6]. They showed that each of the varieties was allergenic, as demonstrated by their ability to inhibit the binding of serum IgE. Our results indicate that the allergenicity of the protein extracts did not depend on heating time, and that proteins with a molecular weight higher than 14 kDa seemed to be heat-stable. Studies have demonstrated that caution should be taken when evaluating the IgE binding capacity of peptide fragments, and that the immunological assay should be chosen with care. For example, digestion products of hazelnut allergens showed no IgE binding capacity when tested by immunoblotting, and the digestion products had a very strong binding capacity in EAST assays [42].

### 3.6. Specific Antibody Binding to Cor a 8 in Electrophoretically Resolved Raw and Roasted Hazelnut Gastric Digesta

**Figure 6:**
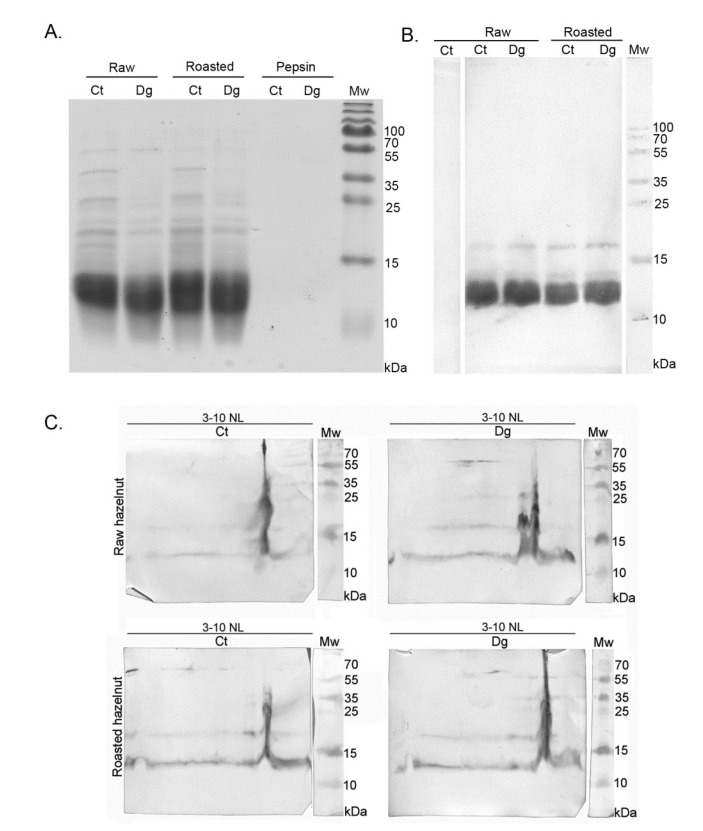
(**A**) 1D SDS-PAGE (14%) protein profiles of non-defatted raw and roasted hazelnut gastric digesta and their corresponding controls. SDS-PAGE was performed in reducing conditions, corresponding to western blot analysis; (**B**) 1D western blot probed with Cor a 8 specific antibody on resolved raw and roasted hazelnut gastric digesta and their controls in reducing conditions; (**C**) 2D western blot probed with Cor a 8 specific antibody on resolved raw and roasted hazelnut gastric digesta and their controls in reducing conditions; Ct—control sample, Dg—gastric phase; Mw—molecular weight markers; kDa—Kilo Daltons; 3–10 NL—nonlinear pH range.

1D gel (Figure 6A) and 1D/2D western blot analyses of hazelnut gastric digesta and corresponding controls were probed with Cor a 8 specific antibodies; the respective immunoreactive maps are presented in Figure 6B. In all four preparations, Cor a 8 (~12 kDa) was recognized by specific antibodies with comparable intensity (Figure 6B,C).

It has been shown that the nsLTP is thermo-labile over 90 °C, probably due to the existence of the lipid-binding tunnel [8], so thermal processing such as dry roasting presumably reduces the allergenicity of Cor a 8. Interestingly, the hazelnut in this study was submitted to roasting at 140 °C, yet Cor a 8 was the only allergen found by mass spectrometry in the roasted digesta (Table S3, Report S1). Comparing raw and roasted hazelnut pairs of gastric digesta and their controls, digesta reacted more intensely with the specific antibody for Cor a 8 (Figure 6C). It is likely that pepsin digestion occurred close to the lipid- binding tunnel, so lipids were released across the entire vertical basic region and prevented easy positioning of the protein to the ideal pI/Mw spot position. Negative control testing was performed on the raw control sample (not shown). These data indicate that hazelnut allergenicity overall originates significantly from Cor a 8 as well as from Cor a 9.

## 4. Conclusions

In this study, we investigated the stability and IgE binding of raw and roasted hazelnut allergens when subjected to gastric digestion. The thermal processing, i.e., roasting, visibly affected the hazelnut protein profiles compared with the raw hazelnut profiles. The overall extractable quantities of proteins and peptides were slightly increased by roasting in the acidic environment (raw control vs. roasted control). In addition, the overall extent of the pepsin digestion was not significantly affected when comparing digestion extents between the raw control and raw digesta, and the roasted control and roasted digesta. 1D immunoblots showed differential IgE responses; within the lower mass region (~12 kDa), IgE binding was dominant in the raw controls, while in regions corresponding to Cor a 9 subunits, slightly stronger IgE binding was observed in roasted hazelnut preparations. 2D immunoblots indicated that roasting slightly reduced the allergenicity of control and digesta compared to their raw counterparts. This was opposite to the IgE ELISA with the pool of hazelnut-allergic patients’ sera, which showed a significant 10% increase in IgE binding in both gastric digesta, probably due to the creation of additional smaller peptides during digestion that retained their immunoreactivity. This discrepancy between results most likely arose from the contribution of conformational epitopes in the IgE ELISA tests.

Cor a 9 in all preparations (raw and roasted) was a dominant contributor to the allergenicity of the whole hazelnut, implying that the immunoreactivity of Cor a 9 was preserved after roasting and after gastric digestion. In support of this, purified raw and roasted Cor a 9 under physiological conditions had a slightly stronger inhibitory effect on the binding of IgE antibodies from the pool of patients’ sera, in all gastric digestions, compared with controls, meaning that there was a slight decrease in the quantity of available Cor a 9 IgE epitopes after 2 h of simulated gastric digestion. In addition, purified roasted Cor a 9 was an even stronger inhibitor in roasted samples than its raw counterpart against raw hazelnut matrices. In addition to possible epitopes of Cor a 9 due to eventual modifications introduced by roasting, this could also point to a decreased relative abundance of Cor a 9 in the roasted samples. Spectroscopic results and mass spectrometry identifications from the 2D gel basic region are in the support of the latter hypothesis. Namely, the presence of Cor a 9 alongside several other allergens (Cor a 8, Cor a 11, Cor a 12, and Cor a 14) was shown in basic smears of the raw samples, in contrast to the roasted hazelnut, where only Cor a 8 was identified. In addition, spectroscopic studies performed on Cor a 9 revealed that the secondary structure did not change upon roasting, but instead the tertiary structure was slightly affected. 

Overall hazelnut allergenicity originates significantly from Cor a 8, as well as from Cor a 9. Further experiments are warranted to elucidate why digesta reacted with greater intensity to the specific antibody for Cor a 8, compared with the controls. 

To summarize, roasting per se did not affect the overall IgE reactivity of the hazelnut, despite the differential electrophoretic profiles and slightly increased protein extraction in the roasted control. However, gastric digestion per se did affect the overall IgE binding, which was increased significantly in the raw and roasted hazelnut gastric digesta. These results point to the likely protective effect of the lipid-rich food matrix that delayed the extraction of proteins. During the simulated gastric digestion, the lipid-rich food matrix was liberated, further delaying gastrointestinal digestion, which may affect allergen-sensitizing capacity and clinical symptoms.

## Data Availability

Data are contained within the article and Supplementary Material.

## Supplementary Materials

The following Supplementary Materials can be downloaded at: https://www.mdpi.com/article/10.3390/foods11182914/s1, Table S1: Use of patients’ sera allergic to hazelnut in study experiments. Marked with “+” was used in the indicated experiment, marked with “/” was not used, Table S2: The modified IEF protocol optimized for focusing of lipid-rich samples conducted on 7 cm IPG-strips, pH 3–10, NL, Figure S1: Excision map of proteins on 14% 2D PAGE profile of raw and roasted hazelnut proteins analyzed by Dionex Ultimate 3000 nLC coupled to Orbitrap Exploris 240 mass spectrometry, Table S3: Identification of proteins spots of raw hazelnut control (Ct) and digest (Dg) achived by Dionex Ultimate 3000 nLC coupled to Orbitrap Exploris 240 mass spectrometry and PEAKS Xpro software, Report S1: Original identification results of proteins and their fragments from basic, smear region od 2D SDS PAGE (Figure S1) of raw and roasted hazelnut digest (Dg) and their counterpart controls (Ct) obtained from PEAKS XPro software, by PEAKS PTM algorithm, Report S2: A representative example of the entire process of digitalization (pixelization).
