# Supplementary material for: INFOGEST Digestion Assay of Raw and Roasted Hazelnuts and Its Impact on Allergens and Their IgE Binding Activity
Source: Foods. 2022 Sep 19;11(18):2914. doi: 10.3390/foods11182914 (PMC9498782; doi:10.3390/foods11182914)
Supplement: Supplementary file 1 [file foods-11-02914-s001.zip › foods-1868141-supplementary.docx]

Supporting Information

INFOGEST digestion assay of raw and roasted hazelnuts and its impact on allergens and their IgE binding activity

Ivana Prodić ^1^, Katarina Smiljanić ^2^, Christoph Nagl ^3^, Barbara Ballmer-Weber ^4^, Karin Hoffmann Sommergruber ^3 *^, Tanja Ćirković Veličković ^2, 5, 6, 7 *^

^1^ University of Belgrade—Faculty of Chemistry, Innovation Center Ltd, 11000 Belgrade, Serbia

^2^ University of Belgrade—Faculty of Chemistry, Center of Excellence for Molecular Food Sciences & Department of Biochemistry, 11000 Belgrade, Serbia

^3^ Department of Pathophysiology and Allergy Research, Medical University of Vienna, 1090 Vienna, Austria

^4^ Allergy Unit, Department of Dermatology, University Hospital Zurich, 8091 Zurich, Switzerland

^5^ Ghent University Global Campus, Incheon 406-840, Korea

^6^ Ghent University, Faculty of Bioscience Engineering, 9000 Ghent, Belgium

^7^ Serbian Academy of Sciences and Arts, 11000 Belgrade, Serbia

^*^ Correspondence: tanja.velickovic@ghent.ac.kr (T.Ć.V.); karin.hoffmann-sommergruber@meduniwien.ac.at (K.H.S.)

**Abbreviations**:

AEBSF – 4-(2-aminoethyl) benzenesulfonyl fluoride hydrochloride

AP - peroxydase

1D – one dimensional

BCA – Bicinchoninic acid

CBB – Coomassie Brilliant Blue

CHAPS – 3-[(3-cholamidopropyl)-dimethylammonio]-1-propanesulfonate

DTT – dithiothreitol

GIT – gastrointestinal tract

GP – gastric phase

IPG - immobilized pH gradient

kDa – Kilo Daltons

MS/MS – tandem mass spectrometry

nLC-MS/MS – nano-liquid chromatography coupled to tandem mass spectrometry

nsLTP – non-specific lipid transfer protein

PBS – phosphate – buffered saline

pNPP ­– p-nitrophenyl Phosphate

PVPP – Polyvinylpolypyrrolidone

PTM – post-translational modification

SDS–PAGE – sodium dodecyl sulphate polyacrylamide gel electrophoresis

SSF – simulated salivary fluid

SGF – simulated gastric fluid

SIF – simulated intestinal fluid

RT – room temperature (between 20°C and 25°C)

TCA – trichloroacetic acid

TBS - tris-buffered saline

TTBS - tris-buffered saline with Tween

WB – Western blot

Supplementary Figures and Tables

**Table S1.** Use of patients’ sera allergic to hazelnut in study experiments. Marked with "+" was used in the indicated experiment, marked with "/" was not used.

| **No. of patients** | **WB (pool)** | **Inhibition ELISA (pool)** | **Direct ELISA** |
| --- | --- | --- | --- |
| 1 | + | / | / |
| 2 | + | / | / |
| 3 | + | / | / |
| 4 | / | / | + |
| 5 | + | + | + |
| 6 | + | + | + |
| 7 | + | + | / |
| 8 | + | + | + |
| 9 | / | + | + |

**Table S2.** The modified IEF protocol optimized for focusing of lipid-rich samples conducted on 7 cm IPG-strips, pH 3-10, NL.

| **Steps** | **Voltage (V)** | **Time (h)** |
| --- | --- | --- |
| 1. Active rehydration | 10 | 12 : 00 |
| 2. Step and hold | 100 | 1 : 00 |
| 3. Step and hold | 300 | 1 : 00 |
| 3. Step and hold | 500 | 1 : 00 |
| 4. Gradient | 1000 | 0 : 45 |
| 5. Gradient | 5000 | 1 : 30 |
| 6. Step and hold | 5000 | 0 : 20 |
| 7. TOTAL | 6061 | 17: 35 |


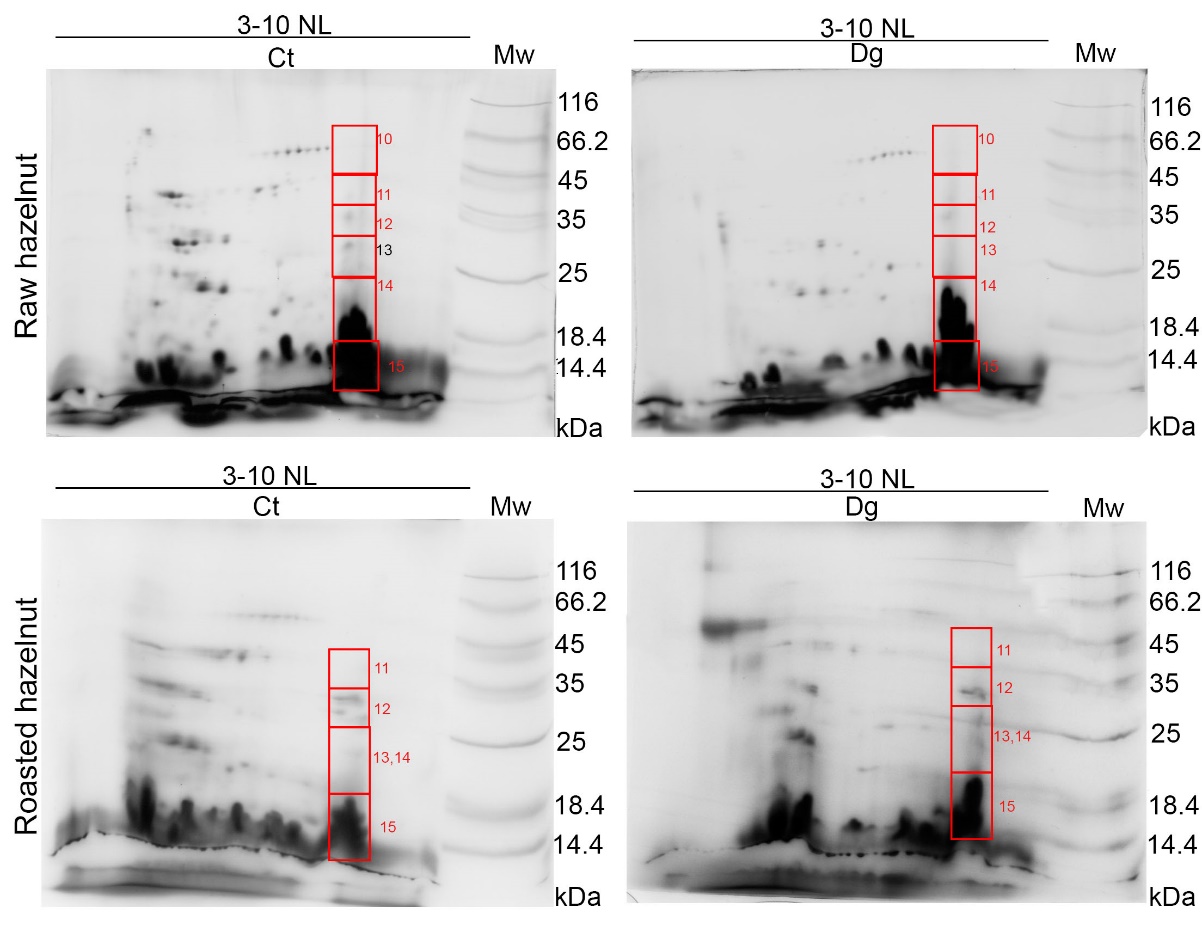


**Figure S1.** Excision map of proteins on 14% 2D PAGE profile of raw and roasted hazelnut proteins analyzed by Dionex Ultimate 3000 nLC coupled to Orbitrap Exploris 240 mass spectrometry.

**Table S3.** Identification of proteins spots of raw hazelnut control (Ct) and digest (Dg) achived by Dionex Ultimate 3000 nLC coupled to Orbitrap Exploris 240 mass spectrometry and PEAKS Xpro software.

| **RAW Hazelnut** | | | | **ROASTED Hazelnut** | | | | **Gel region**  **No from Fig. S2** |
| --- | --- | --- | --- | --- | --- | --- | --- | --- |
| **Control (Ct)** | | **Digest (Dg)** | | **Control (Ct)** | | **Digest (Dg)** | |  |
| **Allergen/**  **Accession No** | **Score** | **Allergen/**  **Accession No** | **Score** | **Allergen/**  **Accession No** | **Score** | **Allergen/**  **Accession No** | **Score** |  |
| Not determined (n. d.) | | n. d. | | Not applicable | | | | **10** |
| n. d. | | Cor a 8 / Q9ATH2 Cor a 9 /A0A0A0P7E3 | 389.9  279.2 | n. d. | | | | **11** |
| n. d. | | Cor a 8 / Q9ATH2 | 102.9 | n. d. | | | | **12** |
| Cor a 9 /A0A0A0P7E3  Cor a 8 / Q9ATH2 | 493.9  228.7 | n. d. | | n. d. | | Cor a 8 / Q9ATH2 | 135.4 | **13** |
| Cor a 9 / Q8W1C2 | 74.4 | n. d. | | n. d. | | | | **14** |
| Cor a 9 /A0A0A0P7E3  Cor a 9 / Q8W1C2  Cor a 8 / Q9ATH2  Cor a 14 D0PWG2 | 454.7  450.6  252.4  62.6 | Cor a 8 / Q9ATH2  Cor a 9 /A0A0A0P7E3  Cor a 9 / Q8W1C2  Cor a 11 / Q8S4P9  Cor a 12, (oleosin) / Q84T21 | 267.6  226.2  221.8  78.9  38.4 | n. d. | | | | **15** |

**Report S1.** Original identification results of proteins and their fragments from basic, smear region od 2D SDS PAGE (Figure S1) of raw and roasted hazelnut digest (Dg) and their counterpart controls (Ct) obtained from PEAKS XPro software, by PEAKS PTM algorithm.
