# Supplementary material for: INFOGEST Digestion Assay of Raw and Roasted Hazelnuts and Its Impact on Allergens and Their IgE Binding Activity
Source: Foods. 2022 Sep 19;11(18):2914. doi: 10.3390/foods11182914 (PMC9498782; doi:10.3390/foods11182914)
Supplement: Supplementary file 1 [file foods-11-02914-s001.zip › Report S1.pdf]

Summary

Spot 11 from RAW digested hazelnut

1. Notes

imam cor a 8, cor a 9, cor a 11 i 2s albumine

2. Result Statistics

Figure 1. False discovery rate (FDR) curve. X axis is the number of peptides being kept. Y axis is the corresponding FDR.

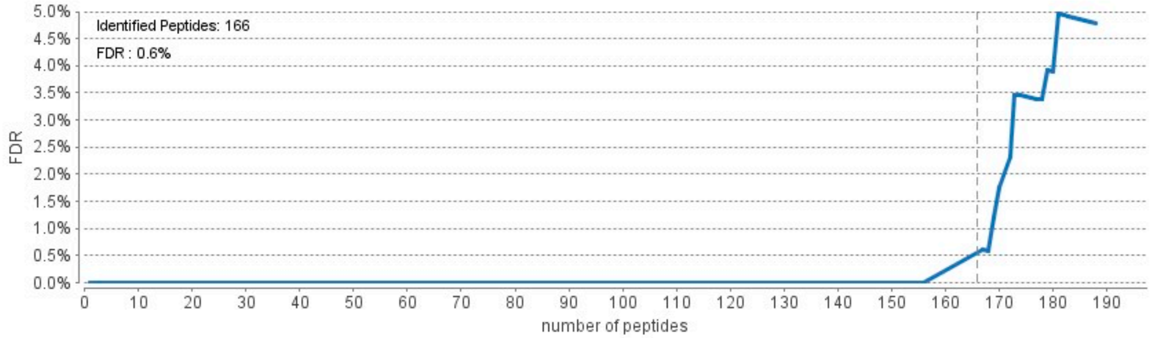

Figure 2. PSM score distribution. **(a)** Distribution of PEAKS peptide score; **(b)** Scatterplot of PEAKS peptide score versus precursor mass error.

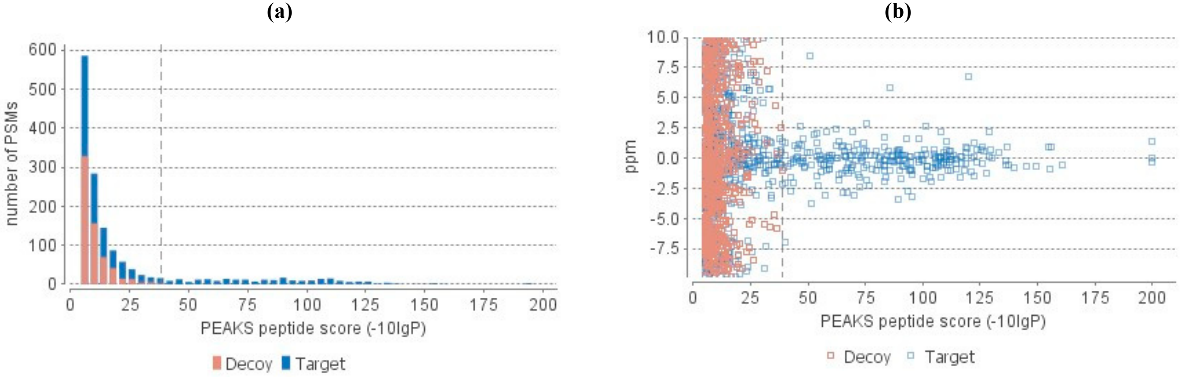

Table 1. Statistics of data.

	#Scans			#Features	Identified			#Peptides	#Sequences	#Proteins*		
	MS1	MS/MS	#Chimera		#PSMs	#Scans	#Features**			Groups	All	Top
Total	3976	5017	13	3904	258	256	127	166	144	5	8	7

gastric RAW 11	3976	5017	13	3904	258	256	127	166	144	5	8	7

* proteins with significant peptides are used in counts.
** features are identified by DB search only.

Figure 3. Sample overlap for Proteins and Peptides (up to 8 samples). **(a)** All Proteins; **(b)** Top Proteins; **(c)** Peptides;

(a)	(b)	(c)
**Not applicable to only one sample**	**Not applicable to only one sample**	**Not applicable to only one sample**

Figure 4. Distribution of peptide feature detection. **(a)** Feature m/z distribution; **(b)** Feature RT distribution.

(a)

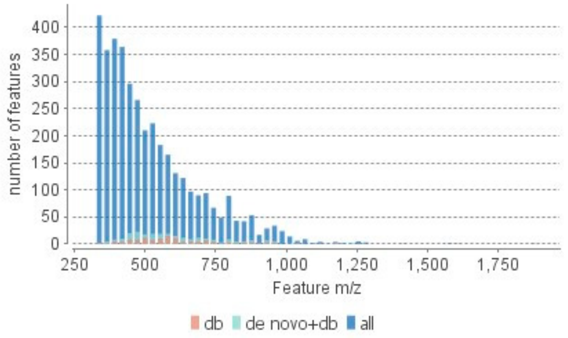

(b)

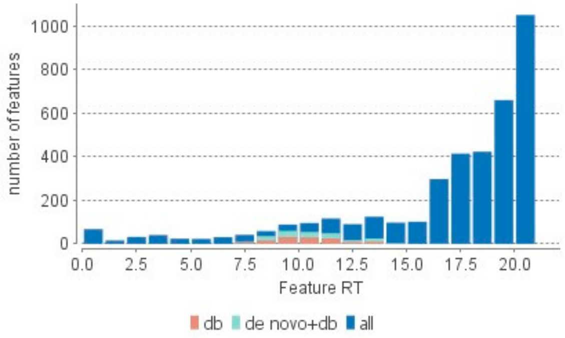

Figure 5. Distribution of identified peptide features. **(a)** Feature abundance distribution; **(b)** *De novo* sequencing validation.

(a)

(b)

proteins

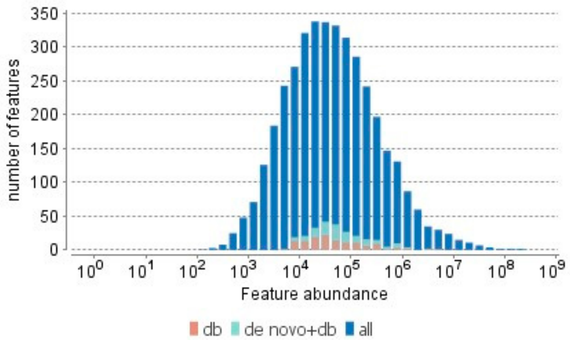

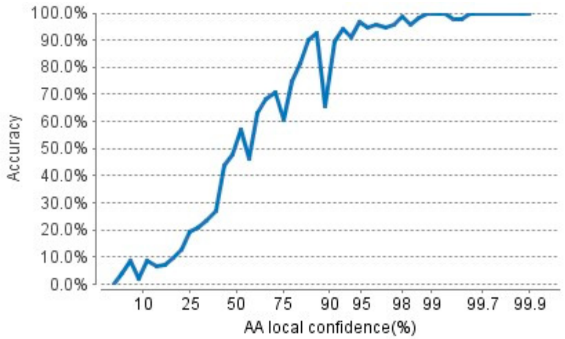

Table 2. Result filtration parameters.

Peptide -10lgP	≥38.6
PTM Ascore	≥20
Protein -10lgP	≥20
Proteins unique peptides	≥1
De novo score(%)	≥50%

Table 4. PTM profile.

Name	ΔMass	Position	#PSM	-10lgP	Abundance	AScore
Carbamidomethyl	57.02	C	48	155.73	2.06E5	1000.00
Oxidation	15.99	M	20	200.00	1.38E6	1000.00
Deamidation	.98	NQ	18	128.72	3.31E5	32.28
Methylation(KR)	14.02	K	4	57.12	9.07E4	1000.00

Table 3. Statistics of filtered result.

FDR (Peptide-Spectrum Matches)	0.4%
FDR (Peptide Sequences)	0.6%
FDR (Protein Group)	0.0%
De Novo Only Spectra	239

3. Experiment Control

Figure 6. Precursor mass error of peptide-spectrum matches (PSM) in filtered result. **(a)** Distribution of precursor mass error in ppm; **(b)** Scatterplot of precursor m/z versus precursor mass error in ppm.

(a) **(b)**

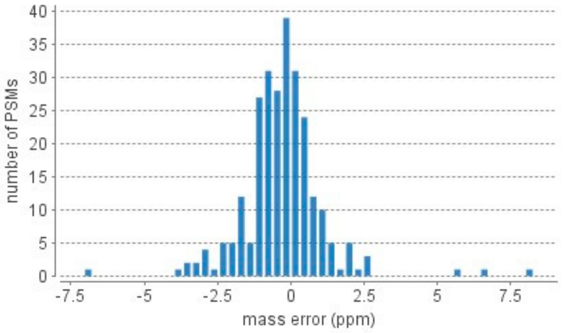

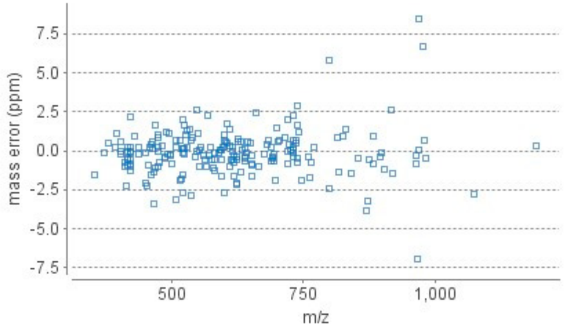

Table 5. Number of identified peptides in each sample by the number of missed cleavages.

Missed Cleavages 0 1 2 3 4+ gastric RAW 11 133 32 1 0 0

4. Other Information

Table 6. Search parameters.
PEAKS Version: PEAKS Studio 10.6 build 20201015
Search Engine Name: PEAKS
Parent Mass Error Tolerance: 10.0 ppm
Fragment Mass Error Tolerance: 0.02 Da
Precursor Mass Search Type: monoisotopic
Enzyme: Trypsin
Max Missed Cleavages: 2
Digest Mode: Semispecific
Fixed Modifications:
Carbamidomethylation: 57.02
Variable Modifications:
Oxidation (M): 15.99
Deamidation (NQ): 0.98

Acetylation (K): 42.01
Acetylation (Protein N-term): 42.01
Acetylation (N-term): 42.01
Amidation: -0.98
Beta-methylthiolation: 45.99
Biotinylation: 226.08 and
303 more...
Max Variable PTM Per Peptide: 3
Database: Corylus aveliana iz 2020 od 156 KB
Taxon: All
Contaminant Database: crap

Table 7. Instrument parameters.
Fractions: 11y.raw
Ion Source: ESI(nano-spray)
Fragmentation Mode: CID, CAD(y and b ions)
MS Scan Mode: FT-ICR/Orbitrap
MS/MS Scan Mode: FT-ICR/Orbitrap

Searched Entry: 602
FDR Estimation: Enabled
De novo score(%) threshold: 15
Peptide hit threshold (-10logP): 30.0
Peaks run ID: 139
Merge Options: no merge
Precursor Options: corrected
Charge Options: no correction
Filter m/z: 200.0 - 2000.0
Filter Charge: 2 - 8
Process: true
Associate chimera: yes

Protein List

Protein Accession Contains:
Protein Description Contains:
Protein Sample Area >=
Protein PTM Contains:

Protein Group	Protein ID	Accession	-10lgP	Coverage (%)	Coverage (%) gastric RAW 11	Area gastric RAW 11	#Peptides	#Unique	#Spec gastric RAW 11	PTM	Avg. Mass	Description
**2**	10069	sp|Q9ATH2|NLTP_CORAV	389.70	67	67	6.332E6	16	16	52	Y	11806	Non-specific lipid-transfer protein Cor a 8 OS=Corylus avella na OX=13451 PE=1 SV=1
**8**	10065	A0A0A0P7E3|A0A0A0P7E3_CORAV	279.17	11	11	3.1802E5	5	5	8	Y	58837	Cor a 9 allergen OS=Corylus avellana OX=13451 PE=2 SV=1
**13**	9619	Q8S4P9|Q8S4P9_CORAV	83.52	2	2	1.3694E4	1	1	1	N	50856	48-kDa glycoprotein OS=Corylus avellana OX=13451 PE=2 S V=1
**12**	10123	D0PWG2|D0PWG2_CORAV	59.32	8	8	2.5304E4	1	1	1	Y	17078	2S albumin OS=Corylus avellana OX=13451 PE=2 SV=1
**14**	10126	C7A7M8|C7A7M8_CORAV	40.07	5	5	0	1	1	1	N	18859	Kinase-like protein (Fragment) OS=Corylus avellana OX=134 51 PE=4 SV=1
**14**	10125	C7A7M9|C7A7M9_CORAV	40.07	5	5	0	1	1	1	N	18844	Kinase-like protein (Fragment) OS=Corylus avellana OX=134 51 PE=4 SV=1
**14**	10161	C7A7N3|C7A7N3_CORAV	40.07	5	5	0	1	1	1	N	18338	Kinase-like protein (Fragment) OS=Corylus avellana OX=134 51 PE=4 SV=1
**total 7 proteins**												

sp|Q9ATH2|NLTP_CORAV

back to list

| Protein Coverage | Supporting Peptides |

Protein Coverage:

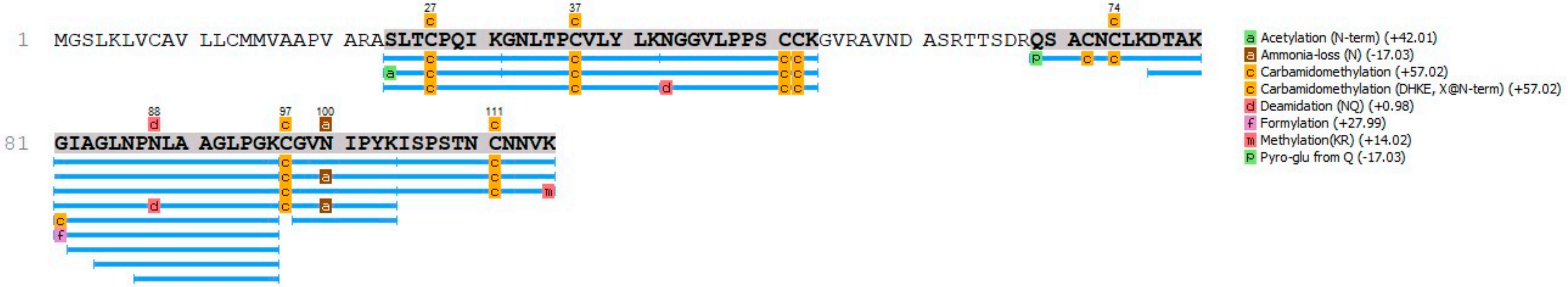

Supporting Peptides:

Peptide	Uniq	-10lgP	Mass	Length	ppm	m/z	z	RT	Fraction	Scan	Source File	Area gastric RAW 11	#Feature	#Feature gastric RAW 11	Start	End	PTM	AScore	Found By
K.C(+57.02)GVNIPYK.I	Y	136.65	949.4691	8	0.9	475.7422	2	10.00	5	3949	11y.raw	6.1132E4	1	1	97	104	Carbamidomethylation	C1:Carbamidomethylation:1000.00	PEAKS DB
K.GIAGLNPNLAAGLPGK.C	Y	130.11	1461.8303	16	0.1	731.9225	2	11.91	5	4706	11y.raw	2.6234E6	1	1	81	96			PEAKS DB
K.GIAGLNPN(+.98)LAAGLPGK.C	Y	128.72	1462.8143	16	2.2	732.4161	2	12.13	5	4794	11y.raw	3.3091E5	1	1	81	96	Deamidation (NQ)	N8:Deamidation (NQ):32.28	PEAKS DB
K.DTAKGIAGLNPNLAAGLPGK.C	Y	126.30	1877.0370	20	0.6	626.6866	3	11.55	5	4569	11y.raw	8.3572E3	1	1	77	96			PEAKS DB
K.GNLTPC(+57.02)VLYLK.N	Y	116.88	1276.6849	11	-0.4	639.3495	2	11.88	5	4683	11y.raw	6.9964E5	1	1	32	42	Carbamidomethylation	C6:Carbamidomethylation:1000.00	PEAKS DB
K.NGGVLPPSC(+57.02)C(+57.02)K.G	Y	112.06	1187.5427	11	0.1	594.7787	2	8.79	5	3465	11y.raw	7.5541E3	1	1	43	53	Carbamidomethylation	C9:Carbamidomethylation:1000.00;C10:Carbamidomethylation:1000.00	PEAKS DB
A.GLNPNLAAGLPGK.C	Y	111.13	1220.6876	13	-1.0	611.3505	2	10.96	5	4344	11y.raw	8.5495E4	1	1	84	96			PEAKS DB
K.GIAGLN(+.98)PNLAAGLPGK.C	Y	107.86	1462.8143	16	2.2	732.4161	2	12.13	5	4831	11y.raw	3.3091E5	1	1	81	96		N6:Deamidation (NQ):14.04	PEAKS DB
K.ISPSTNC(+57.02)NNVK	Y	107.15	1232.5819	11	0.0	617.2982	2	7.80	5	3076	11y.raw	3.8069E5	1	1	105	115	Carbamidomethylation	C7:Carbamidomethylation:1000.00	PEAKS DB

total 28 peptides

K.ISPSTNC(+57.02)N(+.98)NVK	Y	102.01	1233.5659	11	-0.7	617.7898	2	7.97	5	3146	11y.raw	1.3829E5	1	1	105	115	Carbamidomethylation	C7:Carbamidomethylation:1000.00;N8:Deamidation (NQ):17.01	PEAKS DB

Peptide	Uniq	-10lgP	Mass	Length	ppm	m/z	z	RT	Fraction	Scan	Source File	Area gastric RAW 11	#Feature	#Feature gastric RAW 11	Start	End	PTM	AScore	Found By
A.SLTC(+57.02)PQIK.G	Y	92.31	945.4954	8	0.2	473.7551	2	9.38	5	3715	11y.raw	1.1619E6	1	1	24	31	Carbamidomethylation	C4:Carbamidomethylation:1000.00	PEAKS DB
K.ISPSTN(+.98)C(+57.02)NNVK	Y	91.29	1233.5659	11	-0.7	617.7898	2	7.97	5	3196	11y.raw	1.6453E5	2	2	105	115	Carbamidomethylation	N6:Deamidation (NQ):14.04;C7:Carbamidomethylation:1000.00	PEAKS DB
K.N(+.98)GGVLPPSC(+57.02)C(+57.02)K.G	Y	90.73	1188.5267	11	-1.7	595.2697	2	9.70	5	3836	11y.raw	1.2809E4	1	1	43	53	Deamidation (NQ); Carbamidomethylation	N1:Deamidation (NQ):1000.00;C9:Carbamidomethylation:1000.00;C10:Carbamidomethylation:1000.00	PEAKS DB
R.Q(-17.03)SAC(+57.02)NC(+57.02)LKDTAK.G	Y	90.34	1377.6017	12	0.2	689.8083	2	8.71	5	3428	11y.raw	7.316E4	1	1	69	80	Pyro-glu from Q; Carbamidomethylation	Q1:Pyro-glu from Q:1000.00;C4:Carbamidomethylation:1000.00;C6:Carbamidomethylation:1000.00	PEAKS PTM
A.SLTC(+57.02)PQIKGNLTPC(+57.02)VLYLK.N	Y	89.84	2204.1697	19	0.7	735.7310	3	11.84	5	4688	11y.raw	2.2417E4	1	1	24	42	Carbamidomethylation	C4:Carbamidomethylation:1000.00;C14:Carbamidomethylation:1000.00	PEAKS DB

total 28 peptides

K.GIAGLN(+.98)PNLAAGLPGKC(+57.02)GVNIPYK.I	Y	85.46	2394.2729	24	5.8	799.1029	3	11.94	5	4738	11y.raw	3.8441E4	1	1	81	104	Carbamidomethylation	N6:Deamidation (NQ):14.04;C17:Carbamidomethylation:1000.00	PEAKS DB
K.G(+27.99)IAGLNPNLAAGLPGK.C	Y	83.12	1489.8252	16	-1.9	745.9185	2	13.48	5	5328	11y.raw	0	0	0	81	96	Formylation	G1:Formylation:46.00	PEAKS PTM
K.GIAGLNPNLAAGLPGKC(+57.02)GVNIPYK.I	Y	72.72	2393.2888	24	-2.4	798.7683	3	11.99	5	4738	11y.raw	0	0	0	81	104	Carbamidomethylation	C17:Carbamidomethylation:1000.00	PEAKS DB

Peptide	Uniq	-10lgP	Mass	Length	ppm	m/z	z	RT	Fraction	Scan	Source File	Area gastric RAW 11	#Feature	#Feature gastric RAW 11	Start	End	PTM	AScore	Found By
K.C(+57.02)GVN(-17.03)IPYK.I	Y	72.10	932.4426	8	-1.4	467.2279	2	12.00	5	4755	11y.raw	1.9612E4	1	1	97	104	Carbamidomethylation; Ammonia-loss (N)	C1:Carbamidomethylation:1000.00;N4:Ammonia-loss (N):1000.00	PEAKS PTM
C.GVNIPYK.I	Y	71.31	789.4385	7	1.1	395.7270	2	9.77	5	3856	11y.raw	0	0	0	98	104			PEAKS DB
K.C(+57.02)GVN(-17.03)IPYKISPSTNC(+57.02)NNVK	Y	65.18	2147.0139	19	-2.8	1074.5112	2	11.62	5	4600	11y.raw	4.3208E4	2	2	97	115	Carbamidomethylation; Ammonia-loss (N)	C1:Carbamidomethylation:1000.00;N4:Ammonia-loss (N):74.64;C15:Carbamidomethylation:1000.00	PEAKS PTM
K.GNLTPC(+57.02)VLYLKN(+.98)GGVLPPSC(+57.02)C(+57.02)K.G	Y	63.25	2447.2012	22	-1.4	816.7399	3	11.85	5	4684	11y.raw	0	0	0	32	53	Carbamidomethylation	C6:Carbamidomethylation:1000.00;N12:Deamidation (NQ):3.41;C20:Carbamidomethylation:1000.00;C21:Carbamidomethyl	PEAKS DB

total 28 peptides

																		ation:1 000.00	
K.ISPSTNC(+57.02)NNVK(+14.02)	Y	57.12	1246.5975	11	-0.6	624.3057	2	7.93	5	3135	11y.raw	9.0708E4	1	1	105	115	Carbamidomethylation; Methylation(KR)	C7:Carbamidomethylation:1000.00; K11:Methylation(KR):1000.00	PEAKS PTM
G.IAGLNPNLAAGLPGK.C	Y	54.54	1404.8088	15	0.6	703.4121	2	11.74	5	4638	11y.raw	0	0	0	82	96			PEAKS DB
N.PNLAAGLPGK.C	Y	47.76	936.5392	10	1.6	469.2776	2	10.07	5	3973	11y.raw	0	0	0	87	96			PEAKS DB
K.G(+57.02)IAGLNPNLAAGLPGK.C	Y	47.29	1518.8518	16	-0.4	760.4329	2	11.89	5	4712	11y.raw	1.7287E4	1	1	81	96	Carbamidomethylation (DHKE, X@N-term)	G1:Carbamidomethylation (DHKE, X@N-term):36.52	PEAKS PTM

total 28 peptides

Peptide	Uniq	-10lgP	Mass	Length	ppm	m/z	z	RT	Fraction	Scan	Source File	Area gastric RAW 11	#Feature	#Feature gastric RAW 11	Start	End	PTM	AScore	Found By
A.S(+42.01)LTC(+57.02)PQIK.G	Y	45.79	987.5059	8	1.1	494.7608	2	11.04	5	4372	11y.raw	8.612E3	1	1	24	31	Acetylation (N-term); Carbamidomethylation	S1:Acetylation (N-term):1000.00;C4:Carbamidomethylation:1000.00	PEAKS PTM
K.ISPSTN(+.98)C(+57.02)NNVK(+14.02)	Y	43.40	1247.5815	11	-2.1	624.7968	2	8.09	5	3185	11y.raw	1.2981E4	2	2	105	115	Carbamidomethylation; Methylation(KR)	N6:Deamidation (NQ):0.00;C7:Carbamidomethylation:1000.00;K11:Methylation(KR):1000.00	PEAKS PTM
**total 28 peptides**																			

A0A0A0P7E3|A0A0A0P7E3_CORAV

back to list

| Protein Coverage | Supporting Peptides |

Protein Coverage:

```
  1 MAKLILVSFS LCLLVLFNGC LGIDVGLRRQ QQRHFGECNL DRLNALEPTN RIEAEAGQIE SWDHNDQQFQ CAGVAVIRRT
 81 IEPNGLLLPQ YSNAPELIYI ERGRGITGVL FPGCPETFED PQQQSQQGQG QGQSQRSEQD RHQKIRYFQE GDIIALPAGV
161 AHWCYNDGDS PVVAVSLLHT NNYANQLDEN PRHFYLAGNP DDEHQRQGQQ QFGQRRRQQQ HSRGKEGEQE QQGEGNNVFS
241 GFDAEFLADA FNVDVDTARR LQSNQDKRRN IVKVEGRLQM VRPERSRQEW ERQERQERES EQERERQRRQ GGRGRDVNGF
321 EETICSLRLM ENIGSRSRAD IYTEQVGRIN TVNSNTLPVL RWLQLSAERG DLQREGLYVP HWNLNAHSVV YAIRGRARVQ
401 VVDDNGNTVF DDELRQGQVL TIPQNFAVAK RAESEGFEWV AFKTNDNAQI SPLAGRTSAI RALPDDVLAN AFQISREEAR
481 RLKYNRQETT LARSSRSSSE RMRRRSESEG RAEA
```

C Carbamidomethylation (+57.02)
O Oxidation (M) (+15.99)

Supporting Peptides:

Peptide	Uniq	-10lgP	Mass	Length	ppm	m/z	z	RT	Fraction	Scan	Source File	Area gastric RAW 11	#Feature	#Feature gastric RAW 11	Start	End	PTM	AScore	Found By
R.ALPDDVLANAFQISR.E	Y	154.93	1628.8522	15	0.9	815.4341	2	13.42	5	5305	11y.raw	1.0909E5	1	1	462	476			PEAKS DB
R.INTVNSNTLPVLR.W	Y	114.70	1439.8096	13	-0.9	720.9114	2	11.23	5	4438	11y.raw	1.4575E5	1	1	349	361			PEAKS DB
R.LM(+15.99)ENIGSR.S	Y	89.58	934.4542	8	-3.4	468.2328	2	8.43	5	3321	11y.raw	0	0	0	329	336	Oxidation (M)	M2:Oxidation (M):1000.00	PEAKS DB
N.GFEETIC(+57.02)SLR.L	Y	83.13	1210.5652	10	1.7	606.2909	2	11.47	5	4542	11y.raw	2.2967E4	1	1	319	328	Carbamidomethylation	C7:Carbamidomethylation:1000.00	PEAKS DB
R.ADIYTEQVGR.I	Y	81.23	1150.5618	10	-0.6	576.2878	2	9.72	5	3835	11y.raw	1.6183E4	1	1	339	348			PEAKS DB

R.INTVN(+.98)SNTLPVLR.W	Y	54.10	1440.7936	13	2.0	721.4055	2	11.53	5	4560	11y.raw	2.4038E4	1	1	349	361		N5:Deamidation (NQ): 0.00	PEAKS DB
**total 6 peptides**																			

Q8S4P9|Q8S4P9_CORAV

back to list

| Protein Coverage | Supporting Peptides |

Protein Coverage:

```
  1 MLPKEDPELK KCKHKCRDER QFDEQQRRDG KQICEEKARE RQQEEGNSSE ESYGKEQEEN PYVFQDEHFE SRVKTEEGRV
 81 QVLENFTKRS RLLSGIENFR LAILEANPHT FISPAHFDAE LVLFVAKGRA TITMVREEKR ESFNVEHGDI IRIPAGTPVY
161 MINRDENEKL FIVKILQPVS APGHFEAFYG AGGEDPESFY RAFSWEVLEA ALKVRREQLE KVFGEQSKGS IVKASREKIR
241 ALSQHEEGPP RIWPFGGESS GPINLLHKHP SQSNQFGRLY EAHPDDHKQL QDLDLMVSFA NITKGSMAGP YYNSRATKIS
321 VVVEGEGFFE MACPHLSSSS GSYQKISARL RRGVVFVAPA GHPVAVIASQ NNNLQVLCFE VNAHGNSRFP LAGKGNIVNE
401 FERDAKELAF NLPSREVERI FKNQDQAFFF PGPNKQQEEG GRGGRAFE
```

Supporting Peptides:

Peptide	Uniq	-10lgP	Mass	Length	ppm	m/z	z	RT	Fraction	Scan	Source File	Area gastric RAW 11	#Feature	#Feature gastric RAW 11	Start	End	PTM	AScore	Found By
K.GNIVNEFER.D	Y	83.52	1076.5250	9	0.9	539.2703	2	10.78	5	4253	11y.raw	1.3694E4	1	1	395	403			PEAKS DB
**total 1 peptides**																			

D0PWG2|D0PWG2_CORAV

back to list

| Protein Coverage | Supporting Peptides |

Protein Coverage:

```
  1 MARLATLAAL FAALLLVAHA AAFRTTITTV DVDEDIVNQQ GRRGESCREQ AQRQQNLNQC QRYMRQQSQY GSYDGSNQQQ
 81 QQELEQCCQQ LRQMDERCRC EGLRQAVMQQ QGEMRGEEMR EVMETARDLP NQCRLSPQRC EIRSARF
```

Oxidation (M) (+15.99)

Supporting Peptides:

Peptide	Uniq	-10lgP	Mass	Length	ppm	m/z	z	RT	Fraction	Scan	Source File	Area gastric RAW 11	#Feature	#Feature gastric RAW 11	Start	End	PTM	AScore	Found By
R.YM(+15.99)RQQSQYGSYD.G	Y	59.32	1540.6252	12	0.2	771.3201	2	8.81	5	3472	11y.raw	2.5304E4	1	1	63	74	Oxidation (M)	M2:Oxidation (M):100 0.00	PEAKS DB
**total 1 peptides**																			

C7A7M8|C7A7M8_CORAV

back to list

| Protein Coverage | Supporting Peptides |

Protein Coverage:

```
  1 YKGILDQEKR MVAVKVLNLQ QKGASKSFMA ECNALRNIRH RNLVKILTCC SSVDYKGNEF KALVYEFMAN GNLDKWLHHD
 81 RENESPQRYL NLLQRLNIAI DVASSLHYLH DYCETPIIHC DLKPSNVLLD DDMIAKVSDF GLARILFATN DDSQNQTSTA
161 GIKGTI
```

Supporting Peptides:

Peptide	Uniq	-10lgP	Mass	Length	ppm	m/z	z	RT	Fraction	Scan	Source File	Area gastric RAW 11	#Feature	#Feature gastric RAW 11	Start	End	PTM	AScore	Found By
K.RMVAVKVL.N	Y	40.07	914.5735	8	0.2	458.2942	2	16.69	5	6605	11y.raw	0	0	0	10	17			PEAKS DB

total 1 peptides

C7A7M9|C7A7M9_CORAV

back to list

| Protein Coverage | Supporting Peptides |

Protein Coverage:

1 YKGILDQEKR MVAVKVLNLQ QKGASKSFMA ECNALRNIRH RNLVKILTCC SSVDYNGNEF KALVYEFMAN GNLDKWLHHD

81 RENESPQRYL NLLQRLNIAI DVASSLHYLH DYCETPIIHC DLKPSNVLLD DDMIAKVSDF GLARILFATN DDSQNQTSTA

161 GIKGTI

Supporting Peptides:

Peptide	Uniq	-10lgP	Mass	Length	ppm	m/z	z	RT	Fraction	Scan	Source File	Area gastric RAW 11	#Feature	#Feature gastric RAW 11	Start	End	PTM	AScore	Found By
K.RMVAVKVL.N	Y	40.07	914.5735	8	0.2	458.2942	2	16.69	5	6605	11y.raw	0	0	0	10	17			PEAKS DB
total 1 peptides																			

C7A7N3|C7A7N3_CORAV

back to list

| Protein Coverage | Supporting Peptides |

Protein Coverage:

1 ILDKEKRMVA VKVLNLQQKG ASKSFMAECN ALRNIRHRNL VKILTCCSSV DYKGNEFKAL VYEFMENGNL DKWLHHDRDN

81 ESPPRYLNLL QRLNIAIDVA SSLHYLHDHC ETPIIHCDLK PSNVLLDDDM IAKVSDFGLA RIISTTNDAS QNQTSTVGIK

161 GT

Supporting Peptides:

Peptide	Uniq	-10lgP	Mass	Length	ppm	m/z	z	RT	Fraction	Scan	Source File	Area gastric RAW 11	#Feature	#Feature gastric RAW 11	Start	End	PTM	AScore	Found By
K.RMVAVKVL.N	Y	40.07	914.5735	8	0.2	458.2942	2	16.69	5	6605	11y.raw	0	0	0	7	14			PEAKS DB
total 1 peptides																			

Peptide List

Prepared with PEAKS ™ (bioinfor.com)

Summary

Spot 12 from RAW digested hazelnut

1. Notes

Only Cor a 8

2. Result Statistics

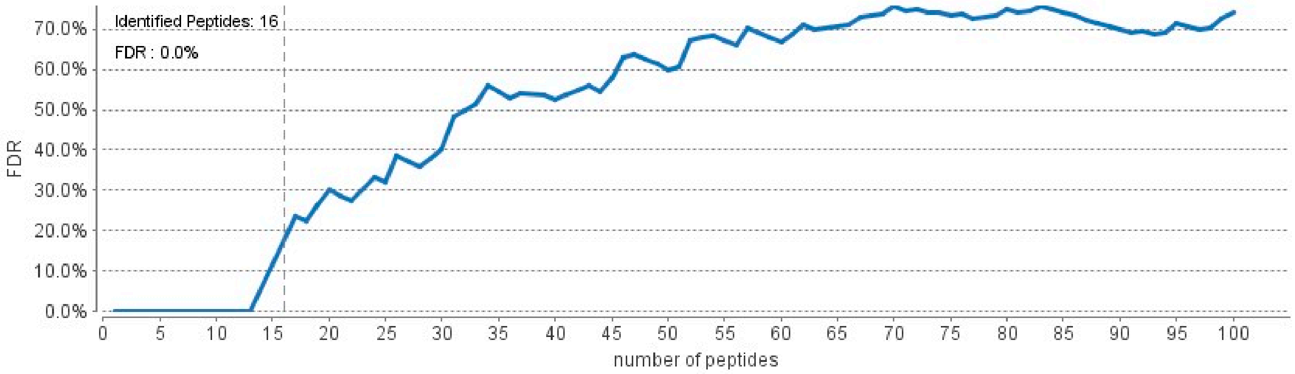

Figure 1. False discovery rate (FDR) curve. X axis is the number of peptides being kept. Y axis is the corresponding FDR.

Figure 2. PSM score distribution. **(a)** Distribution of PEAKS peptide score; **(b)** Scatterplot of PEAKS peptide score versus precursor mass error.

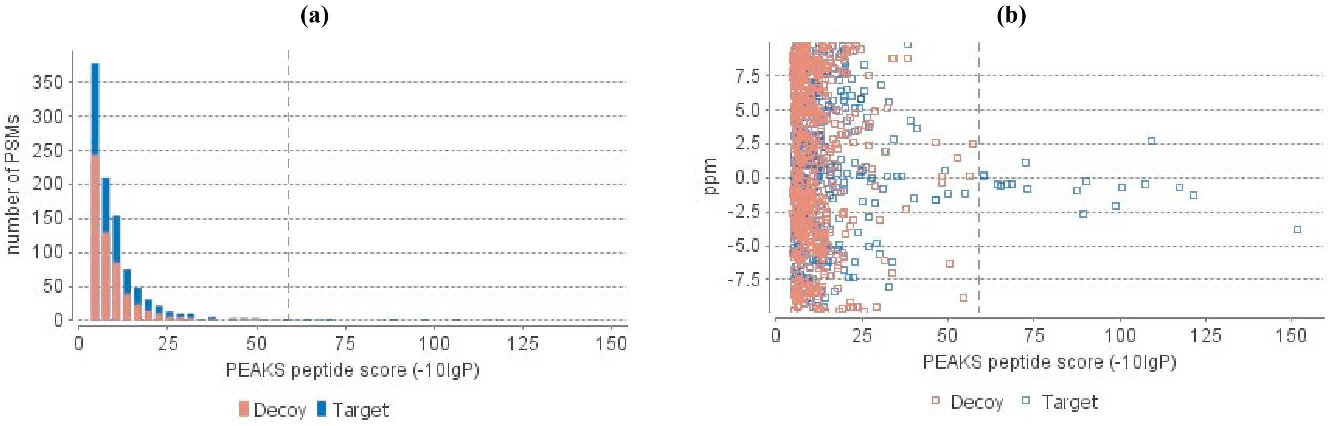

Table 1. Statistics of data.

	#Scans			#Features	Identified			#Peptides	#Sequences	#Proteins*		
	MS1	MS/MS	#Chimera		#PSMs	#Scans	#Features**			Groups	All	Top
Total	4297	4677	3	4128	18	18	13	16	16	1	1	1
Gastric RAW 12	4297	4677	3	4128	18	18	13	16	16	1	1	1

* proteins with significant peptides are used in counts.

** features are identified by DB search only.

Figure 3. Sample overlap for Proteins and Peptides (up to 8 samples). **(a)** All Proteins; **(b)** Top Proteins; **(c)** Peptides;

(a)

Not applicable to only one sample

(b)

Not applicable to only one sample

(c)

Not applicable to only one sample

Figure 4. Distribution of peptide feature detection. **(a)** Feature m/z distribution; **(b)** Feature RT distribution.

(a)

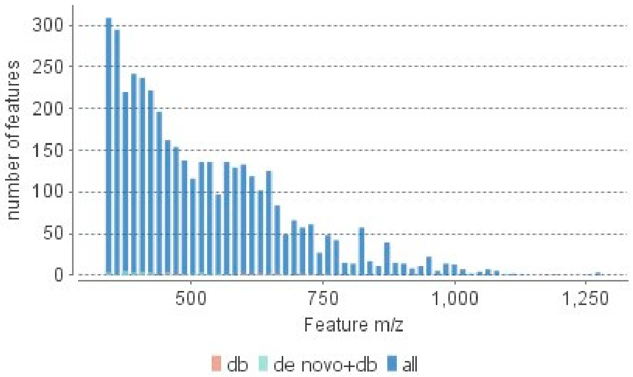

(b)

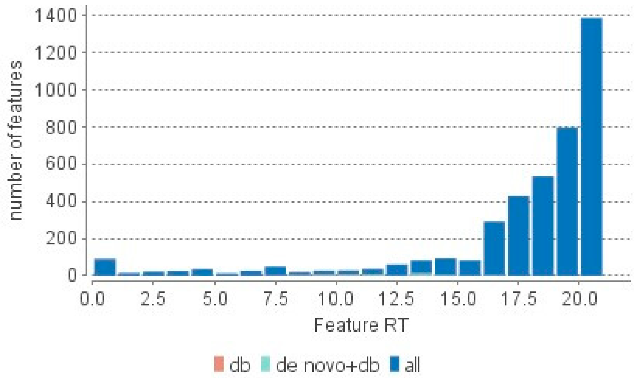

Figure 5. Distribution of identified peptide features. **(a)** Feature abundance distribution; **(b)** *De novo* sequencing validation.

(a)

(b)

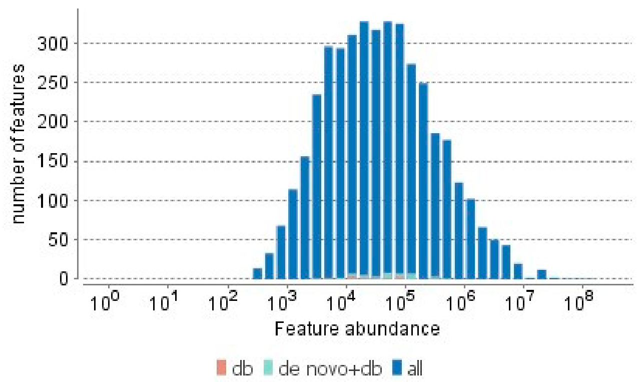

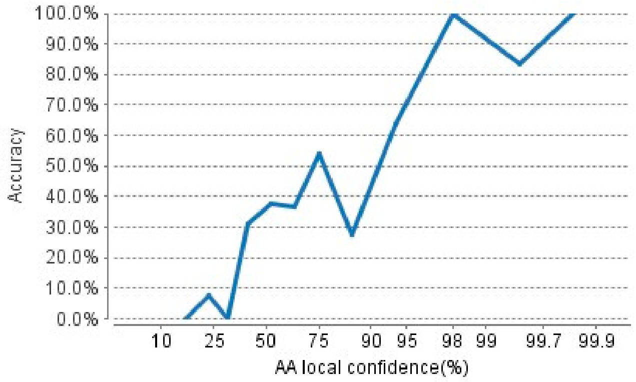

Table 2. Result filtration parameters.

Peptide -10lgP	≥59.1
PTM Ascore	≥20
Protein -10lgP	≥20
Proteins unique peptides	≥1
De novo score(%)	≥50%

Table 3. Statistics of filtered result.

FDR (Peptide-Spectrum Matches)	0.0%
FDR (Peptide Sequences)	0.0%
FDR (Protein Group)	0.0%
De Novo Only Spectra	163

3. Experiment Control

Table 4. PTM profile.

Name	ΔMass	Position	#PSM	-10lgP	Abundance	AScore

Figure 6. Precursor mass error of peptide-spectrum matches (PSM) in filtered result. **(a)** Distribution of precursor mass error in ppm; **(b)** Scatterplot of precursor m/z versus precursor mass error in ppm.

(a)

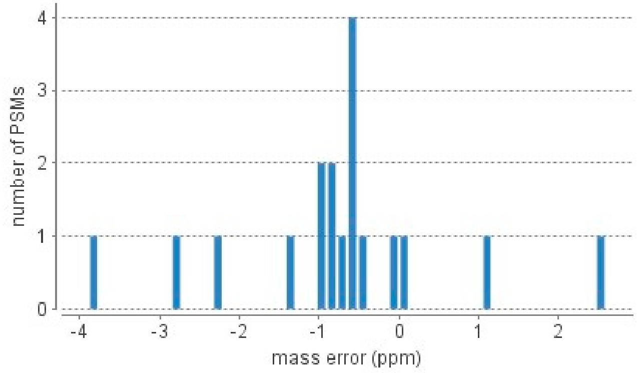

(b)

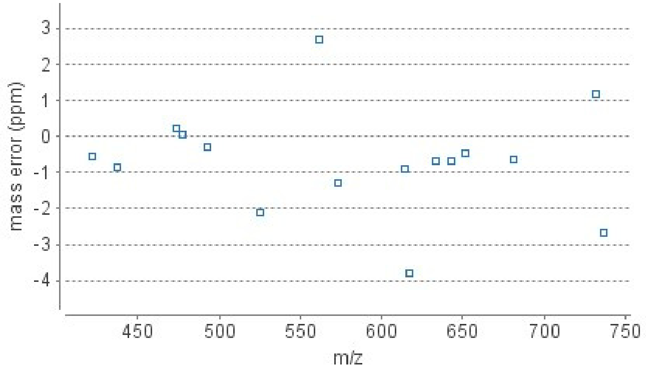

Table 5. Number of identified peptides in each sample by the number of missed cleavages.

Missed Cleavages	0	1	2	3	4+
Gastric RAW 12	13	3	0	0	0

4. Other Information

Table 6. Search parameters.

PEAKS Version: PEAKS Studio 10.6 build 20201015
Search Engine Name: PEAKS
Parent Mass Error Tolerance: 10.0 ppm
Fragment Mass Error Tolerance: 0.02 Da
Precursor Mass Search Type: monoisotopic
Enzyme: Trypsin
Max Missed Cleavages: 2
Digest Mode: Semispecific
Fixed Modifications:
Carbamidomethylation: 57.02
Variable Modifications:
Oxidation (M): 15.99
Deamidation (NQ): 0.98
Searched Entry: 602
FDR Estimation: Enabled
De novo score(%) threshold: 15
Peptide hit threshold (-10logP): 30.0
Peaks run ID: 133
Merge Options: no merge
Precursor Options: corrected
Charge Options: no correction
Filter m/z: 200.0 - 2000.0
Filter Charge: 2 - 8
Process: true
Acetylation (K): 42.01
Acetylation (Protein N-term): 42.01
Acetylation (N-term): 42.01
Amidation: -0.98
Beta-methylthiolation: 45.99
Biotinylation: 226.08 and 303 more...
Max Variable PTM Per Peptide: 3
Database: Corylus aveliana iz 2020 od 156 KB
Taxon: All
Contaminant Database: crap

Table 7. Instrument parameters.

Fractions: 12y.raw
Ion Source: ESI(nano-spray)
Fragmentation Mode: CID, CAD(y and b ions)
MS Scan Mode: FT-ICR/Orbitrap
MS/MS Scan Mode: FT-ICR/Orbitrap

Associate chimera: yes

Protein List

Protein Accession Contains:

Protein Description Contains:

Protein Sample Area >=

Protein PTM Contains:

Protein Group	Protein ID	Accession	-10lgP	Coverage (%)	Coverage (%) Gastric RAW 12	Area Gastric RAW 12	#Peptides	#Unique	#Spec Gastric RAW 12	PTM	Avg. Mass	Description
**5**	10069	sp|Q9ATH2|NLTP_CORAV	102.93	21	21	3.3107E4	2	2	2	Y	11806	Non-specific lipid-transfer protein Cor a 8 OS=Corylus avellana OX=13451 PE=1 SV=1
**total 1 proteins**												

sp|Q9ATH2|NLTP_CORAV

back to list

| Protein Coverage | Supporting Peptides |

Protein Coverage:

27 C

1 MGSLKLVCAV LLCMMVAAPV ARA**SLTCPQI K**GNLTPCVLY LKNGGVLPPS CCKGVRAVND ASRTTSDRQS ACNCLKDTAK

C Carbamidomethylation (+57.02)

81 **GIAGLNPNLA AGLPGK**CGVN IPYKISPSTN CNNVK

Supporting Peptides:

Peptide	Uniq	-10lgP	Mass	Length	ppm	m/z	z	RT	Fraction	Scan	Source File	Area Gastric RAW 12	#Feature	#Feature Gastric RAW 12	Start	End	PTM	AScore	Found By
K.GIAGLNPNLAAGLPGK.C	Y	72.81	1461.8303	16	1.2	731.9233	2	11.88	9	4687	12y.raw	2.1786E4	1	1	81	96			PEAKS DB
A.SLTC(+57.02)PQIK.G	Y	60.23	945.4954	8	0.2	473.7551	2	9.36	9	3708	12y.raw	1.1321E4	1	1	24	31	Carbamidomethylation	C4:Carbamidomethylation:1000.00	PEAKS DB
**total 2 peptides**																			

Peptide List

Prepared with PEAKS ™ (bioinfor.com)

Summary

1. Notes

Spot 13 from RAW control hazelnut

cor a 9 and cor a 8

2. Result Statistics

Figure 1. False discovery rate (FDR) curve. X axis is the number of peptides being kept. Y axis is the corresponding FDR.

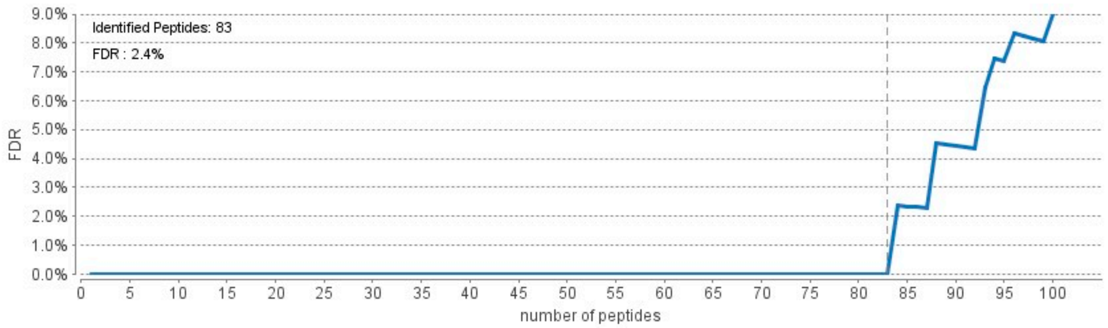

Figure 2. PSM score distribution. **(a)** Distribution of PEAKS peptide score; **(b)** Scatterplot of PEAKS peptide score versus precursor mass error.

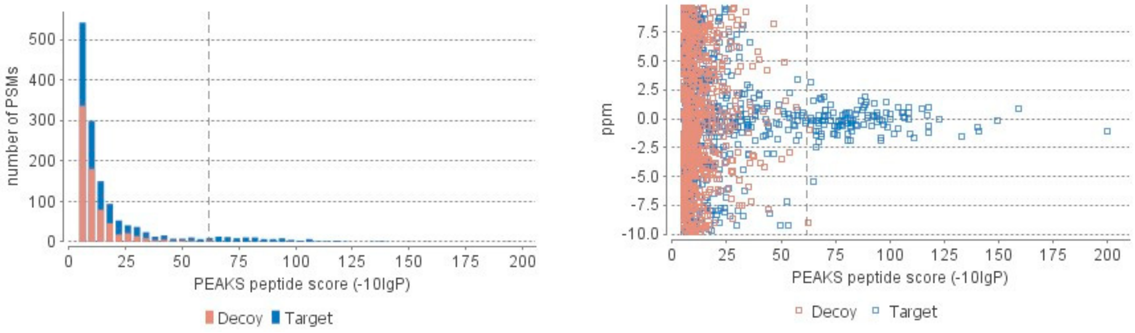

Table 1. Statistics of data.

	#Scans			#Features	Identified			#Peptides	#Sequences	#Proteins*		
	MS1	MS/MS	#Chimera		#PSMs	#Scans	#Features**			Groups	All	Top
Total	3998	4962	22	3832	114	114	68	83	74	2	3	2
Control RAW 13	3998	4962	22	3832	114	114	68	83	74	2	3	2

(a) (b)

* proteins with significant peptides are used in counts.
** features are identified by DB search only.

Figure 3. Sample overlap for Proteins and Peptides (up to 8 samples). **(a)** All Proteins; **(b)** Top Proteins; **(c)** Peptides;

(a)	(b)	(c)
**Not applicable to only one sample**	**Not applicable to only one sample**	**Not applicable to only one sample**

Figure 4. Distribution of peptide feature detection. **(a)** Feature m/z distribution; **(b)** Feature RT distribution.

(a)

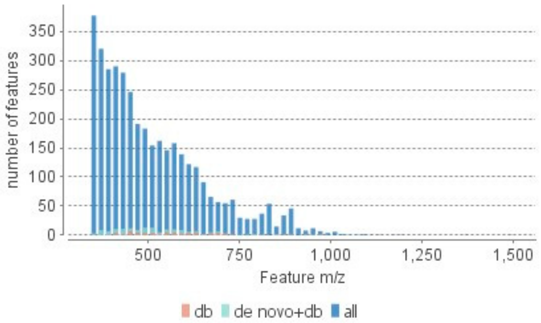

(b)

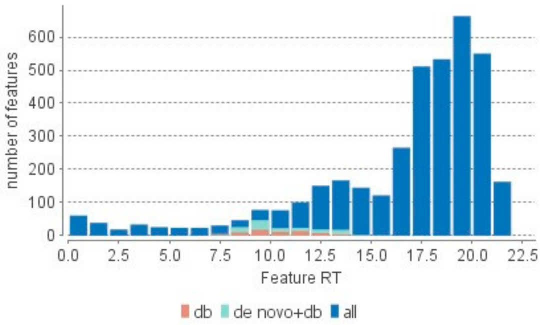

Figure 5. Distribution of identified peptide features. **(a)** Feature abundance distribution; **(b)** *De novo* sequencing validation.

(a) (b)

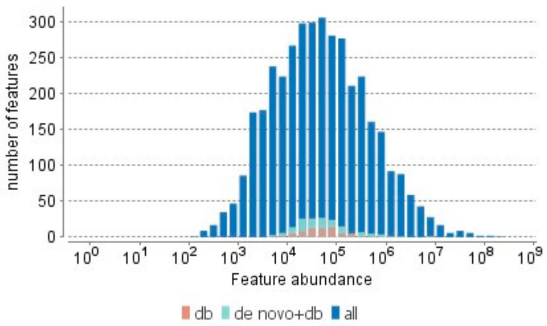

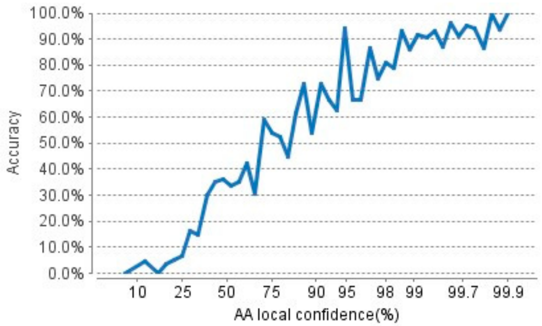

Table 2. Result filtration parameters.

Peptide -10lgP	≥61.7
PTM Ascore	≥20
Protein -10lgP	≥20
Proteins unique peptides	≥1
De novo score(%)	≥50%

Table 3. Statistics of filtered result.

FDR (Peptide-Spectrum Matches)	1.8%
FDR (Peptide Sequences)	2.4%

Table 4. PTM profile.

Name	ΔMass	Position	#PSM	-10lgP	Abundance	AScore
Carbamidomethyl	57.02	C	15	117.48	1.28E6	1000.00

FDR (Protein Group) 0.0%
De Novo Only Spectra 232

3. Experiment Control

Figure 6. Precursor mass error of peptide-spectrum matches (PSM) in filtered result. **(a)** Distribution of precursor mass error in ppm; **(b)** Scatterplot of precursor m/z versus precursor mass error in ppm.

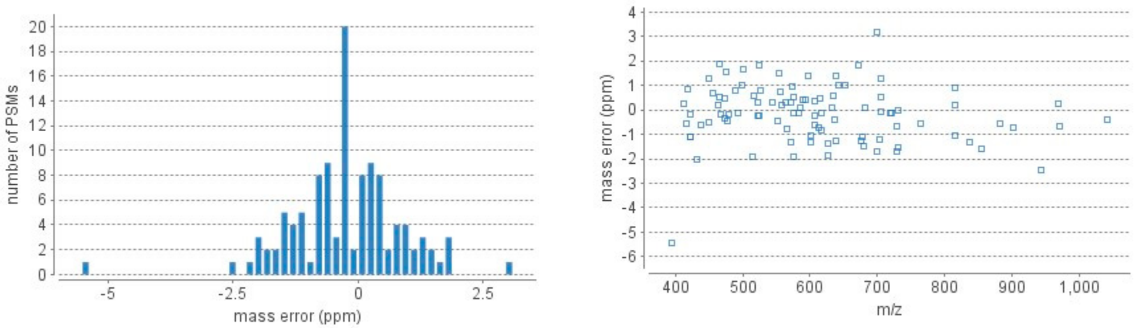

(a) **(b)**

Table 5. Number of identified peptides in each sample by the number of missed cleavages.

Oxidation 15.99 M 5

89.59 5.47E4 1000.00

Missed Cleavages 0 1 2 3 4+ Control

RAW 13 60 23 0 0 0

4. Other Information

Table 6. Search parameters.

PEAKS Version: PEAKS Studio 10.6 build 20201015
Search Engine Name: PEAKS
Parent Mass Error Tolerance: 10.0 ppm
Fragment Mass Error Tolerance: 0.02 Da
Precursor Mass Search Type: monoisotopic
Enzyme: Trypsin
Max Missed Cleavages: 2
Database: Corylus aveliana iz 2020 od 156 KB
Taxon: All
Contaminant Database: crap
Digest Mode: Semispecific
Fixed Modifications:
Carbamidomethylation: 57.02
Variable Modifications:
Oxidation (M): 15.99
Deamidation (NQ): 0.98
Acetylation (K): 42.01
Acetylation (Protein N-term): 42.01
Acetylation (N-term): 42.01
Amidation: -0.98
Beta-methylthiolation: 45.99 Biotinylation: 226.08 and 303 more...
Max Variable PTM Per Peptide: 3

Table 7. Instrument parameters.

Fractions: 13x.raw
Ion Source: ESI(nano-spray)
Fragmentation Mode: CID, CAD(y and b ions)
MS Scan Mode: FT-ICR/Orbitrap
MS/MS Scan Mode: FT-ICR/Orbitrap

Searched Entry: 602
FDR Estimation: Enabled
De novo score(%) threshold: 15
Peptide hit threshold (-10logP): 30.0
Peaks run ID: 94
Merge Options: no merge
Precursor Options: corrected
Charge Options: no correction
Filter m/z: 200.0 - 2000.0
Filter Charge: 2 - 8
Process: true
Associate chimera: yes

Protein List

Protein Accession Contains:
Protein Description Contains:
Protein Sample Area >=
Protein PTM Contains:

Protein Group	Protein ID	Accession	-10lgP	Coverage (%)	Coverage (%) Control RAW 13	Area Control RAW 13	#Peptides	#Unique	#Spec Control RAW 13	PTM	Avg. Mass	Description
**1**	10065	A0A0A0P7E3|A0A0A0P7E3_CORAV	493.98	24	24	2.5794E6	22	22	44	Y	58837	Cor a 9 allergen OS=Corylus avellana OX=13451 PE=2 SV=1
**7**	10069	sp|Q9ATH2|NLTP_CORAV	228.69	40	40	3.4607E5	4	4	6	Y	11806	Non-specific lipid-transfer protein Cor a 8 OS=Corylus avellana OX=13451 PE=1 SV=1
**total 2 proteins**												

A0A0A0P7E3|A0A0A0P7E3_CORAV

back to list

| Protein Coverage | Supporting Peptides |

1 MAKLILVSFS LCLLVLFNGC LGIDVGLRRQ QQRHFGECNL DRLNALEPTN RIEAEAGQIE SWDHNDQQFQ CAGVAVIRRT
81 IEPNGLLLPQ YSNAPELIYI ERGRGITGVL FPGCPETFED PQQQSQQGQG QGQSQRSEQD RHQKIRYFQE GDIIALPAGV
161 AHWCYNDGDS PVVAVSLLHT NNYANQLDEN PRHFYLAGNP DDEHQRQGQQ QFGQRRRQQQ HSRGKEGEQE QQGEGNNVFS
319
241 GFDAEF**LADA FNVDVDTAR**R LQSNQDKRRN IVKVEGRLQM VRPERSRQEW ERQERQERES EQERERQRRQ GGRGRDVN**GF**
325 330
321 **EETICSLRLM ENIGSRSRAD IYTEQVGRIN TVNSNTLPVL R**WLQLSAERG DLQREGLYVP HWNLNAHSVV YAIRGRARVQ
401 VVDDNGNTVF DDELRQGQVL **TIPQNFAVAK** R**AESEGFEWV AFKTNDNAQI SPLAGRTSAI RALPDDVLAN AFQISREEAR**
481 RLK**YNRQETT LAR**SSRSSSE RMRRRSESEG RAEA

a Amidination of lysines or N-terminal amines with methyl acetimidate (+41.03)
c Carbamidomethylation (+57.02)
c Carbamidomethylation (DHKE, X@N-term) (+57.02)
d Dimethylation(KR) (+28.03)
o Oxidation (M) (+15.99)
o 2-OH-ethyl thio-Ser (+60.00)

Protein Coverage:

Supporting Peptides:

Peptide	Uniq	-10lgP	Mass	Length	ppm	m/z	z	RT	Fraction	Scan	Source File	Area Control RAW 13	#Feature	#Feature Control RAW 13	Start	End	PTM	AScore	Found By
R.ALPDDVLANAFQISR.E	Y	200.00	1628.8522	15	-1.1	815.4325	2	13.40	12	5297	13x.raw	9.2226E4	1	1	462	476			PEAKS DB
K.TNDNAQISPLAGR.T	Y	140.88	1355.6793	13	-1.1	678.8462	2	9.86	12	3900	13x.raw	1.6633E4	1	1	444	456			PEAKS DB
F.LADAFNVDVDTAR.R	Y	117.88	1405.6837	13	-1.2	703.8483	2	11.40	12	4498	13x.raw	3.899E4	1	1	247	259			PEAKS DB
N.GFEETIC(+57.02)SLR.L	Y	117.48	1210.5652	10	-0.6	606.2895	2	11.47	12	4538	13x.raw	1.2754E6	1	1	319	328	Carbamidomethylation	C7:Carbamidomethylation:1000.00	PEAKS DB
K.YNRQETTLAR.S	Y	114.64	1250.6367	10	0.8	417.8865	3	7.77	12	3052	13x.raw	3.198E4	1	1	484	493			PEAKS DB
R.AESEGFEWVAFK.T	Y	110.83	1398.6455	12	-1.7	700.3289	2	12.77	12	5048	13x.raw	1.4503E4	1	1	432	443			PEAKS DB
N.DNAQISPLAGR.T	Y	109.40	1140.5887	11	-1.3	571.3009	2	10.13	12	3997	13x.raw	3.6614E4	1	1	446	456			PEAKS DB
R.SRADIYTEQVGR.I	Y	108.50	1393.6949	12	0.5	465.5725	3	8.99	12	3555	13x.raw	1.0572E5	1	1	337	348			PEAKS DB
R.INTVNSNTLPVLR.W	Y	108.44	1439.8096	13	-0.1	720.9120	2	11.25	12	4435	13x.raw	1.0379E5	1	1	349	361			PEAKS DB
R.ALPDDVLANAFQISREEAR.R	Y	105.26	2114.0754	19	0.5	705.6995	3	12.86	12	5082	13x.raw	5.3095E4	1	1	462	480			PEAKS DB
R.TSAIRALPDDVLAN.A	Y	98.56	1454.7728	14	-0.7	728.3932	2	11.74	12	4639	13x.raw	6.2884E4	1	1	457	470			PEAKS DB
N.G(+57.02)FEETIC(+57.02)SLR.L	Y	98.41	1267.5867	10	0.6	634.8010	2	11.52	12	4552	13x.raw	8.2942E4	1	1	319	328	Carbamidomethylation (DHKE, X@N-term); Carbamidomethylation	G1:Carbamidomethylation (DHKE, X@N-term):39.76;C7:Carbamidomethylation:1000.00	PEAKS PTM
N.SNTLPVLR.W	Y	96.85	898.5236	8	-0.5	450.2689	2	10.47	12	4151	13x.raw	1.2044E5	1	1	354	361			PEAKS DB
R.ADIYTEQVGR.I	Y	95.94	1150.5618	10	-1.9	576.2870	2	9.67	12	3814	13x.raw	3.9645E4	1	1	339	348			PEAKS DB
D.NAQISPLAGR.T	Y	82.14	1025.5618	10	-1.9	513.7872	2	9.64	12	3799	13x.raw	3.8301E4	1	1	447	456			PEAKS DB
R.INTVN(+.98)SNTLPVLR.W	Y	81.80	1440.7936	13	-0.1	721.4040	2	11.53	12	4555	13x.raw	0	0	0	349	361		N5:Deamidation (NQ):14.04	PEAKS DB
N.G(+41.03)FEETIC(+57.02)SLR.L	Y	80.55	1251.5918	10	-1.9	626.8020	2	11.47	12	4525	13x.raw	4.8682E4	1	1	319	328	Amidination of lysines or Nterminal amines with methyl acetimidate; Carbamidomethylation	G1:Amidination of lysines or N-terminal amines with methyl acetimidate:1000.00;C7:Carbamidomethylation:1000.00	PEAKS PTM
L.TIPQNFAVAK.R	Y	78.45	1087.6025	10	0.3	544.8087	2	10.23	12	4074	13x.raw	9.1846E4	1	1	421	430			PEAKS DB
R.TSAIRALPDDVLANA.F	Y	78.24	1525.8099	15	-0.6	763.9118	2	12.15	12	4800	13x.raw	0	0	0	457	471			PEAKS DB
N.GFE(+14.02)ETIC(+57.02)SLR.L	Y	75.54	1224.5808	10	-0.7	613.2972	2	11.85	12	4688	13x.raw	8.0941E4	1	1	319	328	Carbamidomethylation	E3:Methylation(others):17.01;C7:Carbamidomethylation:1000.00	PEAKS PTM
R.TSAIRALPDDVLANAF.Q	Y	73.95	1672.8784	16	-1.3	837.4454	2	13.22	12	5226	13x.raw	0	0	0	457	472			PEAKS DB
L.TIPQNFAVAK(+28.03).R	Y	70.38	1115.6339	10	0.2	558.8243	2	10.61	12	4190	13x.raw	9.4634E4	1	1	421	430	Dimethylation(KR)	K10:Dimethylation(KR):1000.00	PEAKS PTM

R.LM(+15.99)ENIGSR.S	Y	69.08	934.4542	8	-0.2	468.2343	2	8.42	12	3303	13x.raw	8.5016E4	1	1	329	336	Oxidation (M)	M2:Oxidation (M):1000.00	PEAKS DB
R.TSAIRALPDDVLA.N	Y	67.76	1340.7300	13	1.8	671.3735	2	11.92	12	4707	13x.raw	0	0	0	457	469			PEAKS DB
C.SLRLM(+15.99)ENIGSR.S	Y	67.34	1290.6714	11	-2.0	431.2302	3	9.37	12	3697	13x.raw	1.7652E4	1	1	326	336	Oxidation (M)	M5:Oxidation (M):1000.00	PEAKS DB
A.QISPLAGR.T	Y	67.12	840.4817	8	-1.1	421.2477	2	9.43	12	3719	13x.raw	2.5825E4	1	1	449	456			PEAKS DB
N.GFEETIC(+57.02)S(+60.00)LR.L	Y	67.10	1270.5686	10	-0.4	636.2913	2	11.93	12	4713	13x.raw	0	0	0	319	328	Carbamidomethylation; 2-OHethyl thio-Ser	C7:Carbamidomethylation:1000.00;S8:2-OH-ethyl thio-Ser:28.36	PEAKS PTM
R.ALPDDVLANAFQISREE.A	Y	65.75	1886.9374	17	-2.5	944.4736	2	13.58	12	5383	13x.raw	2.1722E4	1	1	462	478			PEAKS DB
**total 28 peptides**																			

sp|Q9ATH2|NLTP_CORAV

back to list

| Protein Coverage | Supporting Peptides | Protein

Coverage:

27 C, 37 C

1 MGSLKLVCAV LLCMMVAAPV ARA**SLTCPQI KGNLTPCVLY LK**NGGVLPPS CCKGVRAVND ASRTTSDRQS ACNCLKDTAK

81 **GIAGLNPNLA AGLPGK**CGVN IPYK**ISPSTN CNNVK**

C Carbamidomethylation (+57.02)

Supporting Peptides:

Peptide	Uniq	-10lgP	Mass	Length	ppm	m/z	z	RT	Fraction	Scan	Source File	Area Control RAW 13	#Feature	#Feature Control RAW 13	Start	End	PTM	AScore	Found By
K.GIAGLNPNLAAGLPGK.C	Y	122.74	1461.8303	16	0.0	731.9224	2	11.89	12	4700	13x.raw	2.0148E5	1	1	81	96			PEAKS DB
K.ISPSTNC(+57.02)NNVK	Y	103.80	1232.5819	11	-0.8	617.2977	2	7.80	12	3068	13x.raw	1.6753E4	1	1	105	115	Carbamidomethylation	C7:Carbamidomethylation: 1000.00	PEAKS DB
K.GNLTPC(+57.02)VLYLK.N	Y	101.73	1276.6849	11	-1.3	639.3489	2	11.89	12	4701	13x.raw	6.2313E4	1	1	32	42	Carbamidomethylation	C6:Carbamidomethylation: 1000.00	PEAKS DB
A.SLTC(+57.02)PQIK.G	Y	80.55	945.4954	8	-0.3	473.7548	2	9.40	12	3703	13x.raw	6.5528E4	1	1	24	31	Carbamidomethylation	C4:Carbamidomethylation: 1000.00	PEAKS DB
**total 4 peptides**																			

Peptide List

Prepared with PEAKS ™ (bioinfor.com)

Summary

Spot 14 from RAW control hazelnut

1. Notes

Only Cor a 9

2. Result Statistics

Figure 1. False discovery rate (FDR) curve. X axis is the number of peptides being kept. Y axis is the corresponding FDR.

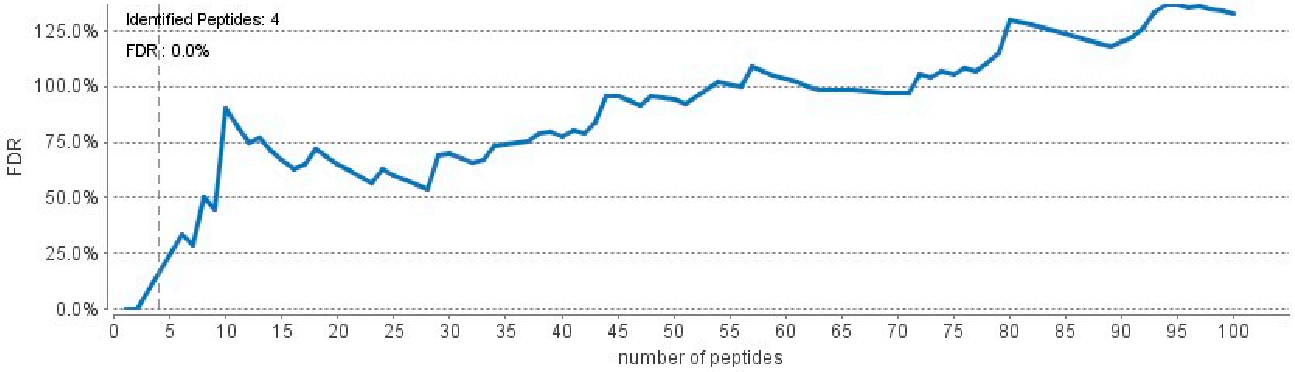

Figure 2. PSM score distribution. **(a)** Distribution of PEAKS peptide score; **(b)** Scatterplot of PEAKS peptide score versus precursor mass error.

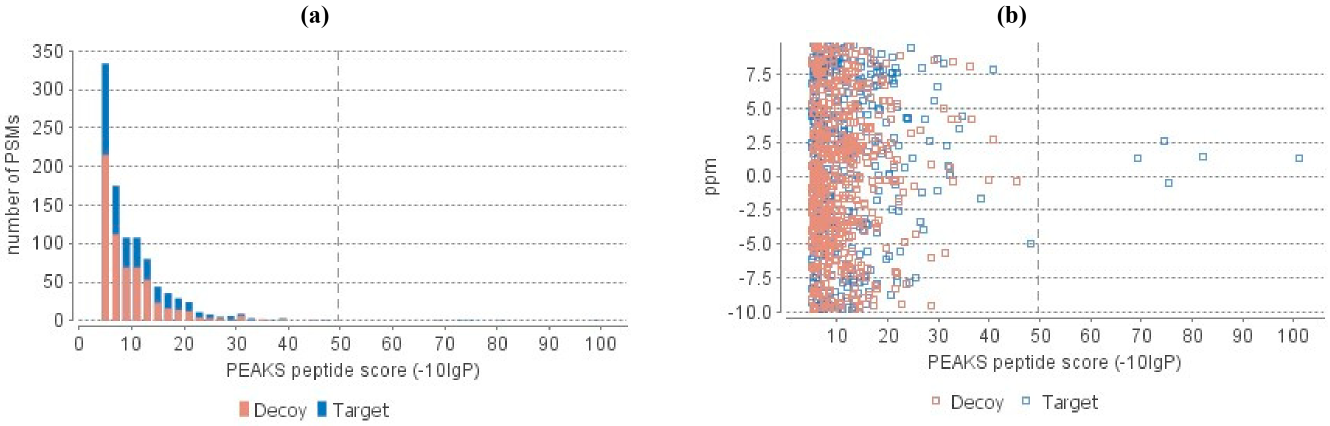

Table 1. Statistics of data.

	#Scans			#Features	Identified			#Peptides	#Sequences	#Proteins*		
	MS1	MS/MS	#Chimera		#PSMs	#Scans	#Features**			Groups	All	Top
Total	4348	4596	12	2187	5	5	1	4	4	1	2	2
Control RAW 14	4348	4596	12	2187	5	5	1	4	4	1	2	2

* proteins with significant peptides are used in counts.
** features are identified by DB search only.

Figure 3. Sample overlap for Proteins and Peptides (up to 8 samples). **(a)** All Proteins; **(b)** Top Proteins; **(c)** Peptides;

(a) **Not applicable to only one sample**

(b) **Not applicable to only one sample**

(c) **Not applicable to only one sample**

Figure 4. Distribution of peptide feature detection. **(a)** Feature m/z distribution; **(b)** Feature RT distribution.

(a)

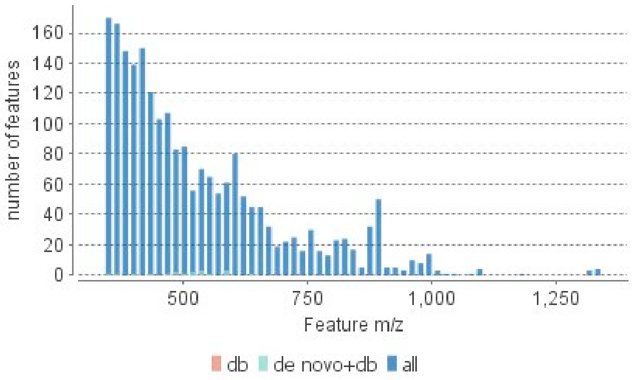

(b)

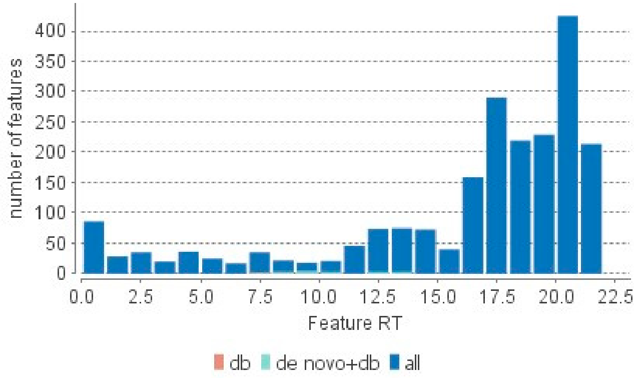

Figure 5. Distribution of identified peptide features. **(a)** Feature abundance distribution; **(b)** *De novo* sequencing validation.

(a)

(b)

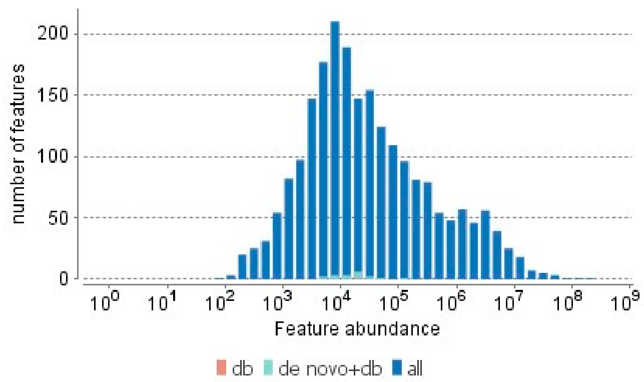

Table 2. Result filtration parameters.

Peptide -10lgP	≥49.6
PTM Ascore	≥20
Protein -10lgP	≥20
Proteins unique peptides	≥1
De novo score(%)	≥50%

Table 3. Statistics of filtered result.

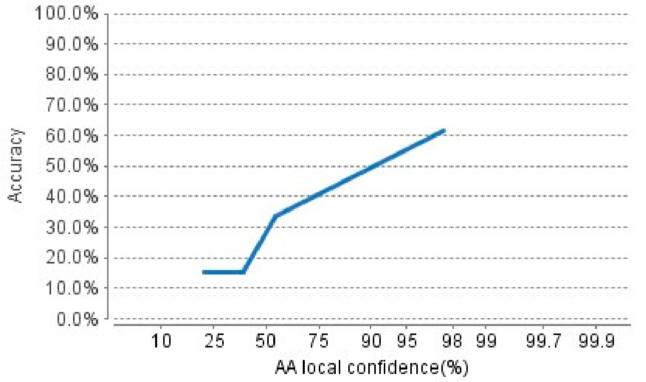

FDR (Peptide-Spectrum Matches)	0.0%
FDR (Peptide Sequences)	0.0%
FDR (Protein Group)	0.0%
De Novo Only Spectra	130

3. Experiment Control

Table 4. PTM profile.

Name	ΔMass	Position	#PSM	-10lgP	Abundance	AScore

Figure 6. Precursor mass error of peptide-spectrum matches (PSM) in filtered result. **(a)** Distribution of precursor mass error in ppm; **(b)** Scatterplot of precursor m/z versus precursor mass error in ppm.

(a) **(b)**

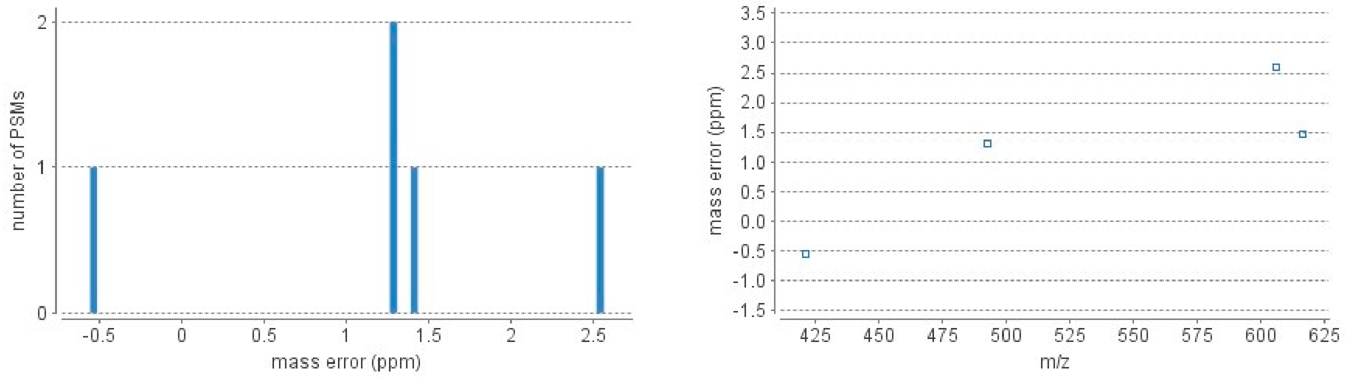

Table 5. Number of identified peptides in each sample by the number of missed cleavages.

Missed Cleavages 0 1 2 3 4+ Control RAW 14 3 1
0 0 0

4. Other Information

Table 6. Search parameters.

PEAKS Version: PEAKS Studio 10.6 build 20201015
Search Engine Name: PEAKS
Parent Mass Error Tolerance: 10.0 ppm
Fragment Mass Error Tolerance: 0.02 Da
Precursor Mass Search Type: monoisotopic
Enzyme: Trypsin
Max Missed Cleavages: 2
Digest Mode: Semispecific
Fixed Modifications:
Carbamidomethylation: 57.02
Variable Modifications: Oxidation (M): 15.99
Deamidation (NQ): 0.98
Acetylation (K): 42.01
Acetylation (Protein N-term): 42.01
Acetylation (N-term): 42.01
Amidation: -0.98
Beta-methylthiolation: 45.99
Biotinylation: 226.08 and 303 more...
Searched Entry: 602
FDR Estimation: Enabled
De novo score(%) threshold: 15
Peptide hit threshold (-10logP): 30.0
Peaks run ID: 103
Merge Options: no merge
Precursor Options: corrected
Charge Options: no correction
Filter m/z: 200.0 - 2000.0
Filter Charge: 2 - 8
Process: true
Max Variable PTM Per Peptide: 3
Database: Corylus aveliana iz 2020 od 156 KB
Taxon: All
Contaminant Database: crap

Table 7. Instrument parameters.

Fractions: 14x.raw
Ion Source: ESI(nano-spray)
Fragmentation Mode: CID, CAD(y and b ions)
MS Scan Mode: FT-ICR/Orbitrap
MS/MS Scan Mode: FT-ICR/Orbitrap

Associate chimera: yes

Protein List

Protein Accession Contains:

Protein Description Contains:

Peptide	Uniq	-10lgP	Mass	Length	ppm	m/z	z	RT	Fraction	Scan	Source File	Area Control RAW 14	#Feature	#Feature Control RAW 14	Start	End	PTM	AScore	Found By

total 1 peptides

Protein Sample Area >=
Protein PTM Contains:

Protein Group	Protein ID	Accession	-10lgP	Coverage (%)	Coverage (%) Control RAW 14	Area Control RAW 14	#Peptides	#Unique	#Spec Control RAW 14	PTM	Avg. Mass	Description
**2**	10065	A0A0A0P7E3|A0A0A0P7E3_CORAV	74.37	2	2	0	1	1	1	Y	58837	Cor a 9 allergen OS=Corylus avellana OX =13451 PE=2 SV=1
**2**	10066	Q8W1C2|Q8W1C2_CORAV	74.37	2	2	0	1	1	1	Y	59127	11S globulin-like protein OS=Corylus avel lana OX=13451 PE=2 SV=1
**total 2 proteins**												

A0A0A0P7E3|A0A0A0P7E3_CORAV

back to list

| Protein Coverage | Supporting Peptides |

Protein Coverage:

C Carbamidomethylation (+57.02)

```
  1 MAKLILVSFS LCLLVLFNGC LGIDVGLRRQ QQRHFGECNL DRLNALEPTN RIEAEAGQIE SWDHNDQQFQ CAGVAVIRRT
 81 IEPNGLLLPQ YSNAPELIYI ERGRGITGVL FPGCPETFED PQQQSQQGQG QGQSQRSEQD RHQKIRYFQE GDIIALPAGV
161 AHWCYNDGDS PVVAVSLLHT NNYANQLDEN PRHFYLAGNP DDEHQRQGQQ QFGQRRRQQQ HSRGKEGEQE QQGEGNNVFS
241 GFDAEFLADA FNVDVDTARR LQSNQDKRRN IVKVEGRLQM VRPERSRQEW ERQERQERES EQERERQRRQ GGRGRDVNGF
321 EETICSLRLM ENIGSRSRAD IYTEQVGRIN TVNSNTLPVL RWLQLSAERG DLQREGLYVP HWNLNAHSVV YAIRGRARVQ
401 VVDDNGNTVF DDELRQGQVL TIPQNFAVAK RAESEGFEWV AFKTNDNAQI SPLAGRTSAI RALPDDVLAN AFQISREEAR
481 RLKYNRQETT LARSSRSSSE RMRRRSESEG RAEA
```

Supporting Peptides:

Peptide	Uniq	-10lgP	Mass	Length	ppm	m/z	z	RT	Fraction	Scan	Source File	Area Control RAW 14	#Feature	#Feature Control RAW 14	Start	End	PTM	AScore	Found By
N.GFEETIC(+57.02)SLR.L	Y	74.37	1210.5652	10	2.6	606.2914	2	11.48	15	4525	14x.raw	0	0	0	319	328	Carbamidomethylation	C7:Carbam idomethyla tion:1000. 00	PEAKS DB
**total 1 peptides**																			

Q8W1C2|Q8W1C2_CORAV

back to list

| Protein Coverage | Supporting Peptides |

Protein Coverage:

C Carbamidomethylation (+57.02)

```
  1 MAKLILVSFS LCLLVLFNGC LGINVGLRRQ QQRYFGECNL DRLNALEPTN RIEAEACQIE SWDHNDQQFQ CAGVAVIRRT
 81 IEPNGLLLPQ YSNAPELIYI ERGRGITGVL FPGCPETFED PQQQSQQGQR QGQGQSQRSE QDRHQKIRHF REGDIIALPA
161 GVAHWCYNDG DSPVVTVSLL HTNNYANQLD ENPRHFYLAG NPDDEHQRQG QQQFGQRRRQ QQHSHGEQGE QEQQGEGNNV
241 FSGFDAEFLA DAFNVDVDTA RRLQSNQDKR RNIVKVEGRL QVVRPERSRQ EWERQERQER ESEQERERQR RQGGRGRDVN
321 GFEETICSLR LRENICTRSR ADIYTEQVGR INTVNSNTLP VLRWLQLSAE RGDLQREGLY VPHWNLNAHS VVYAIRGRAR
401 VQVVDDNGNT VFDDELRQGQ VLTIPQNFAV AKRAESEGFE WVAFKTNDNA QISPLAGRTS AIRALPDDVL ANAFQISREE
481 ARRLKYNRQE TTLVRSSRSS SERKRRSESE GRAEA
```

Supporting Peptides:

Peptide	Uniq	-10lgP	Mass	Length	ppm	m/z	z	RT	Fraction	Scan	Source File	Area Control RAW 14	#Feature	#Feature Control RAW 14	Start	End	PTM	AScore	Found By
N.GFEETIC(+57.02)SLR.L	Y	74.37	1210.5652	10	2.6	606.2914	2	11.48	15	4525	14x.raw	0	0	0	321	330	Carbamidomethylation	C7:Carbamidomethylation:1000.00	PEAKS DB
**total 1 peptides**																			

Peptide List

Prepared with PEAKS ™ (bioinfor.com)

Summary

Spot 15 from RAW control hazelnut

1. Notes

Cor a 9, Cor a 8 & 2s albumins

2. Result Statistics

Figure 1. False discovery rate (FDR) curve. X axis is the number of peptides being kept. Y axis is the corresponding FDR.

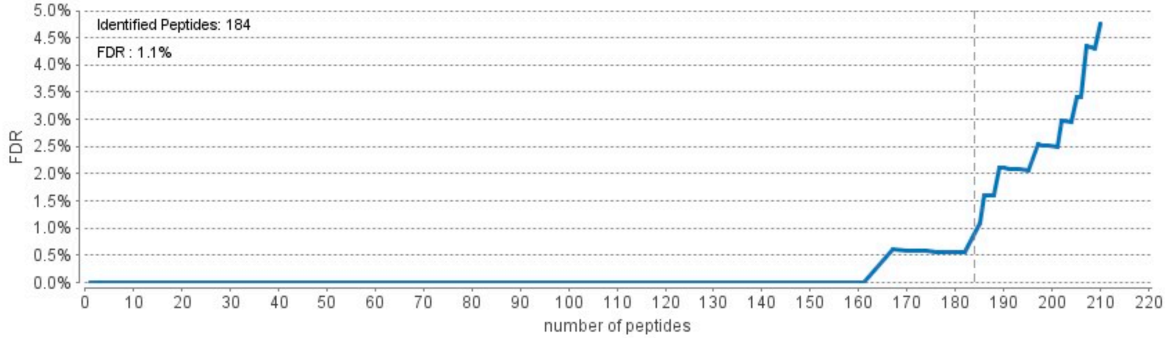

Figure 2. PSM score distribution. **(a)** Distribution of PEAKS peptide score; **(b)** Scatterplot of PEAKS peptide score versus precursor mass error.

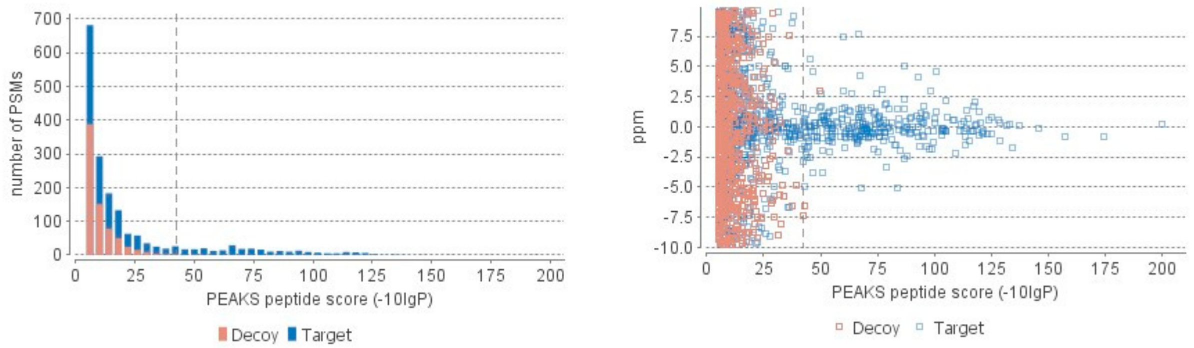

Table 1. Statistics of data.

	#Scans			#Features	Identified			#Peptides	#Sequences	#Proteins*		
	MS1	MS/MS	#Chimera		#PSMs	#Scans	#Features**			Groups	All	Top
Total	3354	5637	27	4287	283	282	147	184	147	5	11	11

(a) (b)

Control RAW 15	3354	5637	27	4287	283	282	147	184	147	5	11	11

* proteins with significant peptides are used in counts.
** features are identified by DB search only.

Figure 3. Sample overlap for Proteins and Peptides (up to 8 samples). **(a)** All Proteins; **(b)** Top Proteins; **(c)** Peptides;

(a) **Not applicable to only one sample**

(b) **Not applicable to only one sample**

(c) **Not applicable to only one sample**

Figure 4. Distribution of peptide feature detection. **(a)** Feature m/z distribution; **(b)** Feature RT distribution.

(a) **(b)**

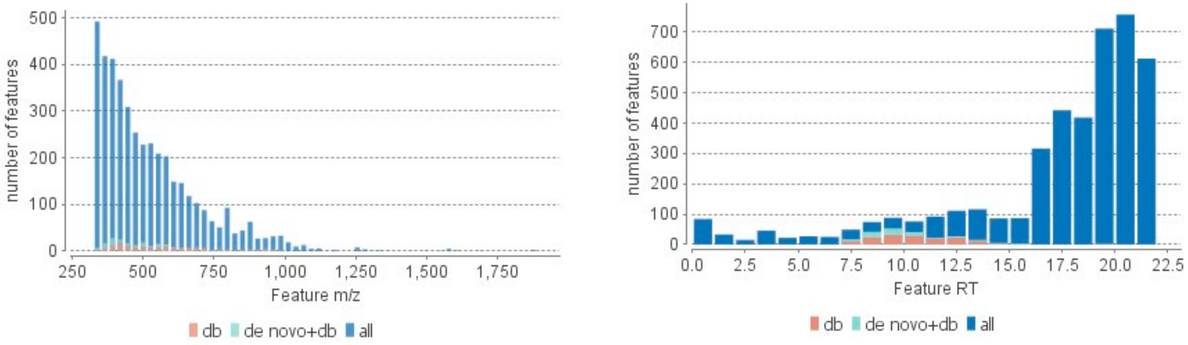

Figure 5. Distribution of identified peptide features. **(a)** Feature abundance distribution; **(b)** *De novo* sequencing validation.

(a) **(b)**

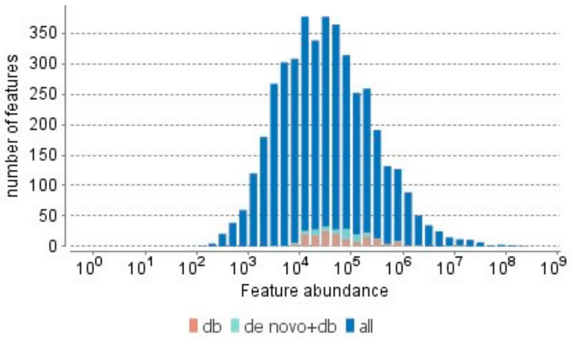

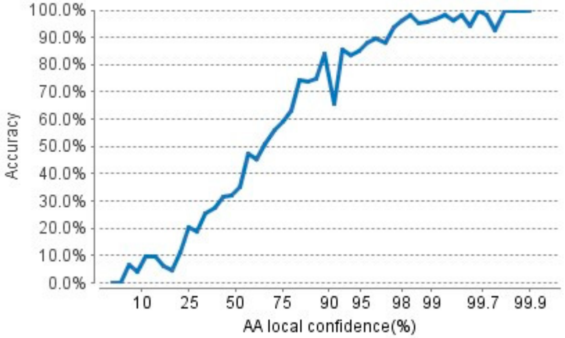

Table 2. Result filtration parameters.

Peptide -10lgP	≥42.6
PTM Ascore	≥20
Protein -10lgP	≥20
Proteins unique peptides	≥1
De novo score(%)	≥50%

Table 3. Statistics of filtered result.

FDR (Peptide-Spectrum Matches)	0.7%
FDR (Peptide Sequences)	1.1%
FDR (Protein Group)	20.0%
De Novo Only Spectra	175

Table 4. PTM profile.

Name	ΔMass	Position	#PSM	-10lgP	Abundance	AScore
Deamidation	.98	NQ	24	121.31	9.45E4	13.67
Oxidation	15.99	M	14	145.91	5.34E4	93.17
Carbamidomethyl	57.02	C	12	112.99	2.4E5	1000.00
Carbamidomethyl	57.02	N-term	7	75.61	3.09E4	45.01

3. Experiment Control

Figure 6. Precursor mass error of peptide-spectrum matches (PSM) in filtered result. **(a)** Distribution of precursor mass error in ppm; **(b)** Scatterplot of precursor m/z versus precursor mass error in ppm.

(a) **(b)**

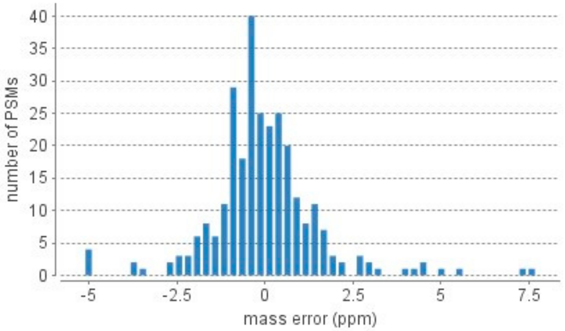

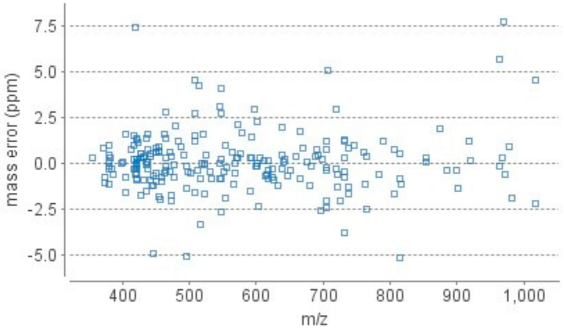

Table 5. Number of identified peptides in each sample by the number of missed cleavages.

Missed Cleavages 0 1 2 3 4+ Control RAW 15 106 74 4 0 0

4. Other Information

Table 6. Search parameters.
PEAKS Version: PEAKS Studio 10.6 build 20201015
Search Engine Name: PEAKS
Parent Mass Error Tolerance: 10.0 ppm
Fragment Mass Error Tolerance: 0.02 Da
Precursor Mass Search Type: monoisotopic
Enzyme: Trypsin
Max Missed Cleavages: 2
Digest Mode: Semispecific
Fixed Modifications:
Carbamidomethylation: 57.02
Variable Modifications:
Oxidation (M): 15.99
Deamidation (NQ): 0.98
Acetylation (K): 42.01
Acetylation (Protein N-term): 42.01
Acetylation (N-term): 42.01
Amidation: -0.98
Beta-methylthiolation: 45.99
Biotinylation: 226.08 and
303 more...
Max Variable PTM Per Peptide: 3
Database: Corylus aveliana iz 2020 od 156 KB
Taxon: All
Contaminant Database: crap
Searched Entry: 602

Table 7. Instrument parameters.
Fractions: 15x.raw
Ion Source: ESI(nano-spray)
Fragmentation Mode: CID, CAD(y and b ions)
MS Scan Mode: FT-ICR/Orbitrap
MS/MS Scan Mode: FT-ICR/Orbitrap

FDR Estimation: Enabled
De novo score(%) threshold: 15
Peptide hit threshold (-10logP): 30.0
Peaks run ID: 108
Merge Options: no merge
Precursor Options: corrected
Charge Options: no correction
Filter m/z: 200.0 - 2000.0
Filter Charge: 2 - 8
Process: true
Associate chimera: yes

Protein List

Protein Accession Contains:
Protein Description Contains:
Protein Sample Area >=
Protein PTM Contains:

Protein Group	Protein ID	Accession	-10lgP	Coverage (%)	Coverage (%) Control RAW 15	Area Control RAW 15	#Peptides	#Unique	#Spec Control RAW 15	PTM	Avg. Mass	Description
**2**	10066	Q8W1C2|Q8W1C2_CORAV	454.66	21	21	3.7691E5	27	4	70	Y	59127	11S globulin-like protein OS=Corylus avellana OX=13451 PE=2 SV=1
**1**	10065	A0A0A0P7E3|A0A0A0P7E3_CORAV	450.60	21	21	1.7825E6	26	3	80	Y	58837	Cor a 9 allergen OS=Corylus avellana OX=13451 PE=2 SV=1
**8**	10069	sp|Q9ATH2|NLTP_CORAV	252.49	57	57	1.3556E6	7	7	14	Y	11806	Non-specific lipid-transfer protein Cor a 8 OS=Corylus avellana OX=13451 PE=1 SV=1
**12**	10123	D0PWG2|D0PWG2_CORAV	62.51	6	6	1.8414E4	1	1	1	Y	17078	2S albumin OS=Corylus avellana OX=13451 PE=2 SV=1
**15**	10100	C7A7I7|C7A7I7_CORAV	49.90	5	5	1.8409E4	1	1	1	N	18167	NBS-containing resistance-like protein (Fragment) OS=Corylus avellana OX=13451 PE=4 SV=1
**15**	10099	C7A748|C7A748_CORAV	49.90	5	5	1.8409E4	1	1	1	N	18297	NBS-containing resistance-like protein (Fragment) OS=Corylus avellana OX=13451 PE=4 SV=1
**15**	10101	C7A766|C7A766_CORAV	49.90	5	5	1.8409E4	1	1	1	N	18332	NBS-containing resistance-like protein (Fragment) OS=Corylus avellana OX=13451 PE=4 SV=1
**15**	10105	C7A767|C7A767_CORAV	49.90	5	5	1.8409E4	1	1	1	N	18828	NBS-containing resistance-like protein (Fragment) OS=Corylus avellana OX=13451 PE=4 SV=1
**15**	10104	C7A763|C7A763_CORAV	49.90	5	5	1.8409E4	1	1	1	N	18886	NBS-containing resistance-like protein (Fragment) OS=Corylus avellana OX=13451 PE=4 SV=1
**15**	10103	C7A770|C7A770_CORAV	49.90	5	5	1.8409E4	1	1	1	N	18902	NBS-containing resistance-like protein (Fragment) OS=Corylus avellana OX=13451 PE=4 SV=1
**15**	10102	C7A765|C7A765_CORAV	49.90	5	5	1.8409E4	1	1	1	N	18943	NBS-containing resistance-like protein (Fragment) OS=Corylus avellana OX=13451 PE=4 SV=1
**total 11 proteins**												

Q8W1C2|Q8W1C2_CORAV

back to list

| Protein Coverage | Supporting Peptides |

Protein Coverage:

1 MAKLILVSFS LCLLVLFNGC LGINVGLRRQ QQRYFGECNL DRLNALEPTN RIEAEACQIE SWDHNDQQFQ CAGVAVIRRT
81 IEPNGLLLPQ YSNAPELIYI ERGRGITGVL FPGCPETFED PQQQSQQGQR QGQGQSQRSE QDRHQKIRHF REGDIIALPA
161 GVAHWCYNDG DSPVVTVSLL HTNNYANQLD ENPRHFYLAG NPDDEHQRQG QQQFGQRRRQ QQHSHGEQGE QEQQGEGNNV
241 FSGFDAEFLA DAFNVDVDTA RRLQSNQDKR RNIVKVEGRL QVVRPERSRQ EWERQERQER ESEQERERQR RQGGRGRDVN
321 GFEETICSLR LRENICTRSR ADIYTEQVGR INTVNSNTLP VLRWLQLSAE RGDLQREGLY VPHWNLNAHS VVYAIRGRAR
401 VQVVDDNGNT VFDDELRQGQ VLTIPQNFAV AKRAESEGFE WVAFKTNDNA QISPLAGRTS AIRALPDDVL ANAFQISREE
481 ARRLKYNRQE TTLVRSSRSS SERKRRSESE GRAEA

c Carbamidomethylation (+57.02)
c Carbamidomethylation (DHKE, X@N-term) (+57.02)
d Deamidation (NQ) (+0.98)

Supporting Peptides:

Peptide	Uniq	-10lgP	Mass	Length	ppm	m/z	z	RT	Fraction	Scan	Source File	Area Control RAW 15	#Feature	#Feature Control RAW 15	Start	End	PTM	AScore	Found By
R.ALPDDVLANAFQISR.E	N	174.35	1628.8522	15	-0.8	815.4327	2	13.41	17	5288	15x.raw	1.9815E5	2	2	464	478			PEAKS DB
K.YNRQETTLVR.S	Y	128.25	1278.6680	10	0.5	427.2302	3	8.30	17	3261	15x.raw	1.8659E5	1	1	486	495			PEAKS DB
F.LADAFNVDVDTAR.R	N	115.74	1405.6837	13	-0.4	703.8489	2	11.37	17	4506	15x.raw	1.7726E6	1	1	249	261			PEAKS DB
N.DNAQISPLAGR.T	N	105.15	1140.5887	11	2.1	571.3029	2	10.13	17	4001	15x.raw	1.3105E4	1	1	448	458			PEAKS DB
L.ANAFQISREEAR.R	N	104.01	1390.6953	12	-0.4	464.5722	3	9.23	17	3633	15x.raw	5.2959E5	2	2	471	482			PEAKS DB

F.KTNDNAQISPLAGR.T	N	102.82	1483.7743	14	-0.1	495.5986	3	8.97	17	3537	15x.raw	4.082E4	1	1	445	458			PEAKS DB

total 41 peptides

Peptide	Uniq	-10lgP	Mass	Length	ppm	m/z	z	RT	Fraction	Scan	Source File	Area Control RAW 15	#Feature	#Feature Control RAW 15	Start	End	PTM	AScore	Found By
R.ALPDDVLANAFQISREEAR.R	N	100.49	2114.0754	19	1.2	705.7000	3	12.86	17	5104	15x.raw	3.6677E5	2	2	464	482			PEAKS DB
L.ANAFQISR.E	N	100.01	905.4719	8	0.6	453.7435	2	9.83	17	3875	15x.raw	2.8406E5	1	1	471	478			PEAKS DB
R.TSAIRALPDDVLANAFQISR.E	N	98.33	2157.1541	20	2.9	720.0607	3	12.99	17	5132	15x.raw	0	0	0	459	478			PEAKS DB
D.AFNVDVDTAR.R	N	95.68	1106.5356	10	1.0	554.2756	2	10.35	17	4087	15x.raw	3.9169E4	1	1	252	261			PEAKS DB
L.ADAFNVDVDTAR.R	N	94.57	1292.5996	12	-0.7	647.3066	2	10.77	17	4268	15x.raw	1.5168E4	1	1	250	261			PEAKS DB
R.ALPDDVLAN(+.98)AFQISREEAR.R	N	86.61	2115.0596	19	5.1	706.0307	3	12.99	17	5134	15x.raw	0	0	0	464	482		N9:Deamidation (NQ):0.00	PEAKS DB
W.VAFKTNDNAQISPLAGR.T	N	86.51	1800.9482	17	2.2	601.3247	3	10.20	17	4030	15x.raw	5.5813E4	1	1	442	458			PEAKS DB
A.FNVDVDTAR.R	N	79.83	1035.4985	9	0.0	518.7565	2	10.19	17	4023	15x.raw	5.5892E4	1	1	253	261			PEAKS DB
F.LADAFNVDVDTARR.L	N	75.75	1561.7848	14	-0.1	521.6022	3	10.60	17	4185	15x.raw	6.478E5	1	1	249	262			PEAKS DB
F.L(+57.02)ADAFNVDVDTAR.R	N	75.61	1462.7052	13	-0.8	732.3593	2	11.49	17	4541	15x.raw	3.0871E4	1	1	249	261	Carbamidomethylation (DHKE, X@N-term)	L1:Carbamidomethylation (DHKE, X@N-term):45.01	PEAKS PTM
K.YNRQ(+.98)ETTLVR.S	Y	73.71	1279.6521	10	0.5	427.5582	3	8.51	17	3357	15x.raw	1.5196E4	1	1	486	495		Q4:Deamidation (NQ):0.00	PEAKS DB
L.A(+57.02)NAFQISR.E	N	73.61	962.4933	8	1.2	482.2545	2	9.87	17	3895	15x.raw	2.1389E4	1	1	471	478	Carbamidomethylation (DHKE, X@N-term)	A1:Carbamidomethylation (DHKE, X@N-term):1000.00	PEAKS PTM
L.AN(+.98)AFQISR.E	N	73.14	906.4559	8	-1.8	454.2344	2	10.19	17	4031	15x.raw	1.4985E4	1	1	471	478	Deamidation (NQ)	N2:Deamidation (NQ):47.09	PEAKS DB
R.INTVNSNTLPVLR.W	N	69.84	1439.8096	13	-0.6	720.9116	2	11.23	17	4456	15x.raw	1.5255E4	1	1	351	363			PEAKS DB
K.YNRQETTL.V	N	67.69	1023.4985	8	-0.4	512.7563	2	8.75	17	3445	15x.raw	5.5087E4	1	1	486	493			PEAKS DB
F.NVDVDTAR.R	N	67.48	888.4301	8	-0.6	445.2220	2	8.13	17	3193	15x.raw	1.3638E5	1	1	254	261			PEAKS DB
D.NAQISPLAGR.T	N	67.35	1025.5618	10	4.3	513.7903	2	9.68	17	3819	15x.raw	0	0	0	449	458			PEAKS DB
N.GFEETIC(+57.02)SLR.L	N	67.02	1210.5652	10	0.3	606.2900	2	11.47	17	4538	15x.raw	1.5075E4	1	1	321	330	Carbamidomethylation	C7:Carbamidomethylation:1000.00	PEAKS DB
R.NIVKVEGR.L	N	65.51	913.5345	8	-0.8	457.7742	2	7.91	17	3130	15x.raw	1.5869E5	1	1	272	279			PEAKS DB
A.FQISREEAR.R	N	63.88	1134.5781	9	-0.6	568.2960	2	8.65	17	3401	15x.raw	6.2552E5	1	1	474	482			PEAKS DB
L.ANAFQ(+.98)ISREEAR.R	N	63.36	1391.6793	12	2.8	464.9017	3	9.43	17	3715	15x.raw	3.5685E4	1	1	471	482	Deamidation (NQ)	Q5:Deamidation (NQ):47.09	PEAKS DB

A.F(+57.02)QISREEAR.R	N	60.14	1191.5996	9	0.0	398.2072	3	8.91	17	3527	15x.raw	9.8057E5	1	1	474	482	Carbamidomethylation (DHKE, X@N-term)	F1:Carbamidomethylation (DHKE, X@N-term):47.14	PEAKS PTM
R.LQVVRPE.R	Y	55.26	839.4865	7	-0.2	420.7504	2	9.35	17	3690	15x.raw	9.8418E4	1	1	280	286			PEAKS DB
R.N(+.98)IVKVEGR.L	N	54.25	914.5185	8	-1.0	458.2661	2	8.57	17	3378	15x.raw	0	0	0	272	279	Deamidation (NQ)	N1:Deamidation (NQ):1000.00	PEAKS DB
F.N(+57.02)VDVDTAR.R	N	53.82	945.4515	8	-2.0	473.7321	2	8.36	17	3295	15x.raw	0	0	0	254	261		N1:Carbamidomethylation (DHKE, X@N-term):14.04	PEAKS PTM
A.FQ(+.98)ISREEAR.R	N	53.80	1135.5621	9	-1.1	379.5276	3	8.83	17	3488	15x.raw	7.3333E5	2	2	474	482	Deamidation (NQ)	Q2:Deamidation (NQ):1000. 00	PEAKS DB
R.QE(+14.02)TTLVR.S	Y	53.48	859.4763	7	0.4	430.7456	2	9.06	17	3569	15x.raw	2.5652E4	1	1	489	495		E2:Methylation(others):10.11	PEAKS PTM
L.AN(+.98)AFQISREEAR.R	N	53.42	1391.6793	12	0.3	464.9005	3	9.72	17	3840	15x.raw	2.5205E4	1	1	471	482	Deamidation (NQ)	N2:Deamidation (NQ):47.09	PEAKS DB
F.LADAFN(+.98)VDVDTAR.R	N	50.81	1406.6677	13	-2.0	704.3397	2	11.63	17	4595	15x.raw	0	0	0	249	261	Deamidation (NQ)	N6:Deamidation (NQ):1000.00	PEAKS DB

total 41 peptides

Peptide	Uniq	-10lgP	Mass	Length	ppm	m/z	z	RT	Fraction	Scan	Source File	Area Control RAW 15	#Feature	#Feature Control RAW 15	Start	End	PTM	AScore	Found By
R.LKYNRQETTLVR.S	Y	50.11	1519.8470	12	0.3	380.9691	4	8.56	17	3369	15x.raw	5.1059E4	2	2	484	495			PEAKS DB
A.FQISREEARR.L	N	49.43	1290.6792	10	0.3	431.2338	3	8.11	17	3186	15x.raw	2.4722E5	1	1	474	483			PEAKS DB
A.FQISR(+14.02)EEAR.R	N	45.09	1148.5938	9	-0.3	383.8717	3	9.12	17	3597	15x.raw	5.8729E5	1	1	474	482		R5:Methylation(KR):8.26	PEAKS PTM
F.NVDVDTARR.L	N	44.87	1044.5312	9	-1.7	523.2720	2	7.54	17	2967	15x.raw	2.9001E4	1	1	254	262			PEAKS DB
N.IVKVEGR.L	N	42.89	799.4916	7	0.0	400.7531	2	7.90	17	3112	15x.raw	0	0	0	273	279			PEAKS DB
R.N(+27.99)IVKVEGR.L	N	42.89	941.5294	8	0.6	471.7723	2	9.64	17	3802	15x.raw	0	0	0	272	279		N1:Formylation:12.33	PEAKS PTM

total 41 peptides

A0A0A0P7E3|A0A0A0P7E3_CORAV

back to list

| Protein Coverage | Supporting Peptides |

Protein Coverage:

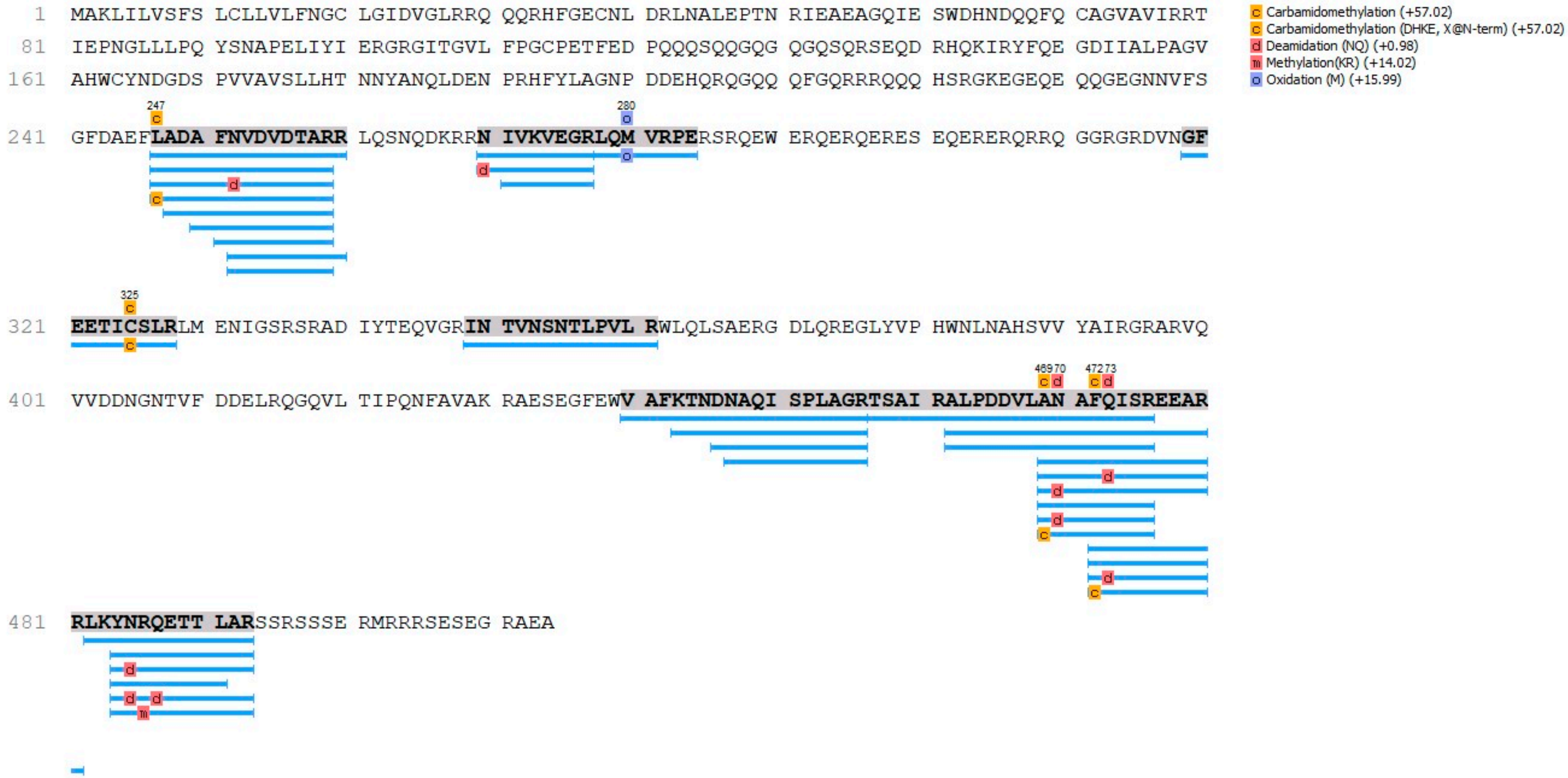

Supporting Peptides:

Peptide	Uniq	-10lgP	Mass	Length	ppm	m/z	z	RT	Fraction	Scan	Source File	Area Control RAW 15	#Feature	#Feature Control RAW 15	Start	End	PTM	AScore	Found By
R.ALPDDVLANAFQISR.E	N	174.35	1628.8522	15	-0.8	815.4327	2	13.41	17	5288	15x.raw	1.9815E5	2	2	462	476			PEAKS DB
K.YNRQ(+.98)ETTLAR.S	Y	121.31	1251.6207	10	-0.6	418.2139	3	7.98	17	3147	15x.raw	9.4455E4	1	1	484	493		Q4:Deamidation (NQ):13.67	PEAKS DB
K.YNRQETTLAR.S	Y	121.17	1250.6367	10	-0.3	417.8860	3	7.75	17	3054	15x.raw	1.1452E6	3	3	484	493			PEAKS DB
F.LADAFNVDVDTAR.R	N	115.74	1405.6837	13	-0.4	703.8489	2	11.37	17	4506	15x.raw	1.7726E6	1	1	247	259			PEAKS DB
N.DNAQISPLAGR.T	N	105.15	1140.5887	11	2.1	571.3029	2	10.13	17	4001	15x.raw	1.3105E4	1	1	446	456			PEAKS DB
L.ANAFQISREEAR.R	N	104.01	1390.6953	12	-0.4	464.5722	3	9.23	17	3633	15x.raw	5.2959E5	2	2	469	480			PEAKS DB

F.KTNDNAQISPLAGR.T	N	102.82	1483.7743	14	-0.1	495.5986	3	8.97	17	3537	15x.raw	4.082E4	1	1	443	456			PEAKS DB
R.ALPDDVLANAFQISREEAR.R	N	100.49	2114.0754	19	1.2	705.7000	3	12.86	17	5104	15x.raw	3.6677E5	2	2	462	480			PEAKS DB
L.ANAFQISR.E	N	100.01	905.4719	8	0.6	453.7435	2	9.83	17	3875	15x.raw	2.8406E5	1	1	469	476			PEAKS DB
R.TSAIRALPDDVLANAFQISR.E	N	98.33	2157.1541	20	2.9	720.0607	3	12.99	17	5132	15x.raw	0	0	0	457	476			PEAKS DB
D.AFNVDVDTAR.R	N	95.68	1106.5356	10	1.0	554.2756	2	10.35	17	4087	15x.raw	3.9169E4	1	1	250	259			PEAKS DB
L.ADAFNVDVDTAR.R	N	94.57	1292.5996	12	-0.7	647.3066	2	10.77	17	4268	15x.raw	1.5168E4	1	1	248	259			PEAKS DB
R.ALPDDVLAN(+.98)AFQISREEAR.R	N	86.61	2115.0596	19	5.1	706.0307	3	12.99	17	5134	15x.raw	0	0	0	462	480		N9:Deamidation (NQ):0.00	PEAKS DB
W.VAFKTNDNAQISPLAGR.T	N	86.51	1800.9482	17	2.2	601.3247	3	10.20	17	4030	15x.raw	5.5813E4	1	1	440	456			PEAKS DB
K.YN(+.98)RQETTLAR.S	Y	86.15	1251.6207	10	-0.3	418.2141	3	8.20	17	3232	15x.raw	2.2138E4	1	1	484	493	Deamidation (NQ)	N2:Deamidation (NQ):26.31	PEAKS DB
A.FNVDVDTAR.R	N	79.83	1035.4985	9	0.0	518.7565	2	10.19	17	4023	15x.raw	5.5892E4	1	1	251	259			PEAKS DB
R.LKYNRQETTLAR.S	Y	78.44	1491.8158	12	-0.4	498.2790	3	8.12	17	3192	15x.raw	3.0662E5	2	2	482	493			PEAKS DB
F.LADAFNVDVDTARR.L	N	75.75	1561.7848	14	-0.1	521.6022	3	10.60	17	4185	15x.raw	6.478E5	1	1	247	260			PEAKS DB
F.L(+57.02)ADAFNVDVDTAR.R	N	75.61	1462.7052	13	-0.8	732.3593	2	11.49	17	4541	15x.raw	3.0871E4	1	1	247	259	Carbamidomethylation (DHKE, X@N-term)	L1:Carbamidomethylation (DHKE, X@N-term):45.01	PEAKS PTM
L.A(+57.02)NAFQISR.E	N	73.61	962.4933	8	1.2	482.2545	2	9.87	17	3895	15x.raw	2.1389E4	1	1	469	476	Carbamidomethylation (DHKE, X@N-term)	A1:Carbamidomethylation (DHKE, X@N-term):1000.00	PEAKS PTM
L.AN(+.98)AFQISR.E	N	73.14	906.4559	8	-1.8	454.2344	2	10.19	17	4031	15x.raw	1.4985E4	1	1	469	476	Deamidation (NQ)	N2:Deamidation (NQ):47.09	PEAKS DB
R.INTVNSNTLPVLR.W	N	69.84	1439.8096	13	-0.6	720.9116	2	11.23	17	4456	15x.raw	1.5255E4	1	1	349	361			PEAKS DB
K.YNRQETTL.A	N	67.69	1023.4985	8	-0.4	512.7563	2	8.75	17	3445	15x.raw	5.5087E4	1	1	484	491			PEAKS DB
F.NVDVDTAR.R	N	67.48	888.4301	8	-0.6	445.2220	2	8.13	17	3193	15x.raw	1.3638E5	1	1	252	259			PEAKS DB
D.NAQISPLAGR.T	N	67.35	1025.5618	10	4.3	513.7903	2	9.68	17	3819	15x.raw	0	0	0	447	456			PEAKS DB
N.GFEETIC(+57.02)SLR.L	N	67.02	1210.5652	10	0.3	606.2900	2	11.47	17	4538	15x.raw	1.5075E4	1	1	319	328	Carbamidomethylation	C7:Carbamidomethylation:1000.00	PEAKS DB
R.NIVKVEGR.L	N	65.51	913.5345	8	-0.8	457.7742	2	7.91	17	3130	15x.raw	1.5869E5	1	1	270	277			PEAKS DB
A.FQISREEAR.R	N	63.88	1134.5781	9	-0.6	568.2960	2	8.65	17	3401	15x.raw	6.2552E5	1	1	472	480			PEAKS DB
**total 44 peptides**																			
**Peptide**	**Uniq**	**-10lgP**	**Mass**	**Length**	**ppm**	**m/z**	**z**	**RT**	**Fraction**	**Scan**	**Source File**	**Area Control RAW 15**	**#Feature**	**#Feature Control RAW 15**	**Start**	**End**	**PTM**	**AScore**	**Found By**

L.ANAFQ(+.98)ISREEAR.R	N	63.36	1391.6793	12	2.8	464.9017	3	9.43	17	3715	15x.raw	3.5685E4	1	1	469	480	Deamidation (NQ)	Q5:Deamidation (NQ):47.09	PEAKS DB
A.F(+57.02)QISREEAR.R	N	60.14	1191.5996	9	0.0	398.2072	3	8.91	17	3527	15x.raw	9.8057E5	1	1	472	480	Carbamidomethylation (DHKE, X@N-term)	F1:Carbamidomethylation (D HKE, X@N-term):47.14	PEAKS PTM
K.YN(+.98)RQ(+.98)ETTLAR.S	Y	60.01	1252.6047	10	7.4	418.5453	3	7.72	17	3040	15x.raw	0	0	0	484	493	Deamidation (NQ)	N2:Deamidation (NQ):1000.00;Q4:Deamidation (NQ):1000.00	PEAKS DB
R.N(+.98)IVKVEGR.L	N	54.25	914.5185	8	-1.0	458.2661	2	8.57	17	3378	15x.raw	0	0	0	270	277	Deamidation (NQ)	N1:Deamidation (NQ):1000.00	PEAKS DB
F.N(+57.02)VDVDTAR.R	N	53.82	945.4515	8	-2.0	473.7321	2	8.36	17	3295	15x.raw	0	0	0	252	259		N1:Carbamidomethylation (D HKE, X@N-term):14.04	PEAKS PTM
A.FQ(+.98)ISREEAR.R	N	53.80	1135.5621	9	-1.1	379.5276	3	8.83	17	3488	15x.raw	7.3333E5	2	2	472	480	Deamidation (NQ)	Q2:Deamidation (NQ):1000.00	PEAKS DB
K.YNR(+14.02)QETTLAR.S	Y	53.51	1264.6523	10	1.1	422.5585	3	8.25	17	3253	15x.raw	1.2146E4	1	1	484	493	Methylation(KR)	R3:Methylation(KR):52.22	PEAKS PTM
L.AN(+.98)AFQISREEAR.R	N	53.42	1391.6793	12	0.3	464.9005	3	9.72	17	3840	15x.raw	2.5205E4	1	1	469	480	Deamidation (NQ)	N2:Deamidation (NQ):47.09	PEAKS DB
R.LQM(+15.99)VRPE.R	Y	53.04	887.4535	7	-1.2	444.7335	2	8.26	17	3257	15x.raw	2.0195E5	1	1	278	284	Oxidation (M)	M3:Oxidation (M):1000.00	PEAKS DB
F.LADAFN(+.98)VDVDTAR.R	N	50.81	1406.6677	13	-2.0	704.3397	2	11.63	17	4595	15x.raw	0	0	0	247	259	Deamidation (NQ)	N6:Deamidation (NQ):1000.00	PEAKS DB
A.FQISREEARR.L	N	49.43	1290.6792	10	0.3	431.2338	3	8.11	17	3186	15x.raw	2.4722E5	1	1	472	481			PEAKS DB
A.FQISR(+14.02)EEAR.R	N	45.09	1148.5938	9	-0.3	383.8717	3	9.12	17	3597	15x.raw	5.8729E5	1	1	472	480		R5:Methylation(KR):8.26	PEAKS PTM
F.NVDVDTARR.L	N	44.87	1044.5312	9	-1.7	523.2720	2	7.54	17	2967	15x.raw	2.9001E4	1	1	252	260			PEAKS DB
R.LKYNRQ(+.98)ETTLAR.S	Y	43.55	1492.7998	12	-0.8	374.2069	4	8.33	17	3282	15x.raw	0	0	0	482	493		Q6:Deamidation (NQ):0.00	PEAKS DB
N.IVKVEGR.L	N	42.89	799.4916	7	0.0	400.7531	2	7.90	17	3112	15x.raw	0	0	0	271	277			PEAKS DB
R.N(+27.99)IVKVEGR.L	N	42.89	941.5294	8	0.6	471.7723	2	9.64	17	3802	15x.raw	0	0	0	270	277		N1:Formylation:12.33	PEAKS PTM
**total 44 peptides**																			

sp|Q9ATH2|NLTP_CORAV

back to list

| Protein Coverage | Supporting Peptides |

Protein Coverage:

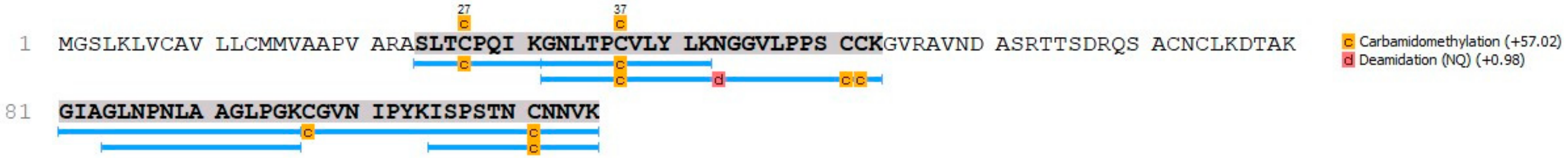

Supporting Peptides:

Peptide	Uniq	-10lgP	Mass	Length	ppm	m/z	z	RT	Fraction	Scan	Source File	Area Control RAW 15	#Feature	#Feature Control RAW 15	Start	End	PTM	AScore	Found By
K.GIAGLNPNLAAGLPGK.C	Y	120.62	1461.8303	16	-0.4	731.9221	2	11.89	17	4698	15x.raw	7.1362E5	1	1	81	96			PEAKS DB
K.GNLTPC(+57.02)VLYLK.N	Y	112.99	1276.6849	11	0.5	639.3500	2	11.87	17	4699	15x.raw	2.3996E5	1	1	32	42	Carbamidomethylation	C6:Carbamidomethylation:1000.00	PEAKS DB

total 8 peptides

Peptide	Uniq	-10lgP	Mass	Length	ppm	m/z	z	RT	Fraction	Scan	Source File	Area Control RAW 15	#Feature	#Feature Control RAW 15	Start	End	PTM	AScore	Found By
A.SLTC(+57.02)PQIK.G	Y	78.75	945.4954	8	0.4	473.7551	2	9.38	17	3708	15x.raw	3.2805E5	1	1	24	31	Carbamidomethylation	C4:Carbamidomethylation:1000.00	PEAKS DB
K.ISPSTNC(+57.02)NNVK	Y	68.24	1232.5819	11	0.2	617.2983	2	7.80	17	3074	15x.raw	1.335E4	1	1	105	115	Carbamidomethylation	C7:Carbamidomethylation:1000.00	PEAKS DB
K.GNLTPC(+57.02)VLYLKN(+.98)GGVLPPSC(+57.02)C(+57.02)K.G	Y	66.89	2447.2012	22	-1.2	816.7401	3	11.84	17	4674	15x.raw	0	0	0	32	53	Carbamidomethylation; Deamidation (NQ)	C6:Carbamidomethylation:1000.00;N12:Deamidation (NQ):21.51;C20:Carbamidomethylation:1000.00;C21:Carbamidomethylation:1000.00	PEAKS DB
K.C(+57.02)GVNIPYKISPSTNC(+57.02)NNVK	Y	65.90	2164.0405	19	-1.3	722.3532	3	10.33	17	4089	15x.raw	1.2868E4	1	1	97	115	Carbamidomethylation	C1:Carbamidomethylation:1000.00;C15:Carbamidomethylation:1000.00	PEAKS DB
K.GIAGLNPN(+.98)LAAGLPGK.C	Y	55.44	1462.8143	16	1.3	732.4154	2	12.09	17	4791	15x.raw	1.7785E4	1	1	81	96		N8:Deamidation (NQ):14.04	PEAKS DB
A.GLNPNLAAGLPGK.C	Y	53.89	1220.6876	13	0.2	611.3512	2	10.96	17	4340	15x.raw	3.0011E4	1	1	84	96			PEAKS DB

total 8 peptides

D0PWG2|D0PWG2_CORAV

back to list

| Protein Coverage | Supporting Peptides |

Protein Coverage:

1 MARLATLAAL FAALLLVAHA AAFRTTITTV DVDEDIVNQQ GRRGESCREQ AQRQQNLNQC QRYMRQQSQY GSYDGSNQQQ

81 QQELEQCCQQ LRQMDERCRC EGLRQAVMQQ QGEMRGEEMR EVMETARDLP NQCR**LSPQRC EIR**SARF

C Carbamidomethylation (+57.02)

Supporting Peptides:

Peptide	Uniq	-10lgP	Mass	Length	ppm	m/z	z	RT	Fraction	Scan	Source File	Area Control RAW 15	#Feature	#Feature Control RAW 15	Start	End	PTM	AScore	Found By
R.LSPQRC(+57.02)EIR.S	Y	62.51	1157.5975	9	-0.6	386.8729	3	7.94	17	3120	15x.raw	1.8414E4	1	1	135	143	Carbamidomethylation	C6:Carbamidomethylation:1000.00	PEAKS DB
total 1 peptides																			

C7A7I7|C7A7I7_CORAV

back to list

| Protein Coverage | Supporting Peptides |

Protein Coverage:

1 LAEIVYKRIA DKFDASCFIP NVREETKNYG LLHLQKQVIS EILGESETNI HNLCVATNMI ANRIRKRRVL IILDDVDEDE

81 QVEALAKSHN WFGHGSRIII TSRDLHFLNK LEVNTYKVKK LNNEEALELF SWKAFKKPHP EENYVELS**KD IVSYAQ**

Supporting Peptides:

Peptide	Uniq	-10lgP	Mass	Length	ppm	m/z	z	RT	Fraction	Scan	Source File	Area Control RAW 15	#Feature	#Feature Control RAW 15	Start	End	PTM	AScore	Found By
S.KDIVSYAQ	Y	49.90	922.4760	8	1.6	462.2460	2	9.95	17	3928	15x.raw	1.8409E4	1	1	149	156			PEAKS DB
total 1 peptides																			

C7A748|C7A748_CORAV

back to list

| Protein Coverage | Supporting Peptides |

Protein Coverage:

1 LAEIIYKRIA NKFEASCFIH NVREETKNYG LLHLQKQVIY EILSESETNI QNLCVATNII ANRIRKRRVL IILDDVDEDE

81 QVEALAMSHN WFGQGSRIII TSRDLHFLNR LEVNTYKVKK LNNTEALELF SWKAFKKSHP EENYVELS**KD IVSYAQ**

Supporting Peptides:

Peptide	Uniq	-10lgP	Mass	Length	ppm	m/z	z	RT	Fraction	Scan	Source File	Area Control RAW 15	#Feature	#Feature Control RAW 15	Start	End	PTM	AScore	Found By
S.KDIVSYAQ	Y	49.90	922.4760	8	1.6	462.2460	2	9.95	17	3928	15x.raw	1.8409E4	1	1	149	156			PEAKS DB
total 1 peptides																			

C7A766|C7A766_CORAV

back to list

| Protein Coverage | Supporting Peptides |

Protein Coverage:

1 TLAKVIYESI AYKFEASCFI PNIREQTKRH GLLYLQIQLI SQILGESETN IQINTCVATS MIANRIRQRR VLIILDDMDG
81 DEQVQALAGS HDWFGQGSRI IITSRDLHFL KIGLGVGDDA YEVELLNNEE ALELFSRKAF KKSHPKENYV ELS**KDIVSYA**
161 **Q**

Supporting Peptides:

Peptide	Uniq	-10lgP	Mass	Length	ppm	m/z	z	RT	Fraction	Scan	Source File	Area Control RAW 15	#Feature	#Feature Control RAW 15	Start	End	PTM	AScore	Found By
S.KDIVSYAQ	Y	49.90	922.4760	8	1.6	462.2460	2	9.95	17	3928	15x.raw	1.8409E4	1	1	154	161			PEAKS DB
**total 1 peptides**																			

C7A767|C7A767_CORAV

back to list

| Protein Coverage | Supporting Peptides |

Protein Coverage:

1 TLAKVIYESI AYKFEASCFI PNIREQTKKH GLLYLQIQLI SQILGESETN IQINTCVATS MIANRIRQRR VLIILGDMDG
81 DEQVQALAGS HDWFGQGSRI IITSRDLHFL KIVLRDVGDV GDDTYLVELL NNEEALELFS WKAFKKPHPE KNYVELS**KDI**
161 **VSYAQ**

Supporting Peptides:

Peptide	Uniq	-10lgP	Mass	Length	ppm	m/z	z	RT	Fraction	Scan	Source File	Area Control RAW 15	#Feature	#Feature Control RAW 15	Start	End	PTM	AScore	Found By
S.KDIVSYAQ	Y	49.90	922.4760	8	1.6	462.2460	2	9.95	17	3928	15x.raw	1.8409E4	1	1	158	165			PEAKS DB
**total 1 peptides**																			

C7A763|C7A763_CORAV

back to list

| Protein Coverage | Supporting Peptides |

Protein Coverage:

1 TLAKVIYESI AYKFEASCFI PNIREQTKKH GLLYLQIQLI SQILGESETN IQINTCVATS MIANRIRQRR VLIILDDMDG
81 DEQVQALAGS HDWFGQGSRI IITSRDLHFL KIVLRDVGDV GDDTYLVELL NNEEALELFS WKAFKKPHPE KNYVELS**KDI**
161 **VSYAQ**

Supporting Peptides:

Peptide	Uniq	-10lgP	Mass	Length	ppm	m/z	z	RT	Fraction	Scan	Source File	Area Control RAW 15	#Feature	#Feature Control RAW 15	Start	End	PTM	AScore	Found By
S.KDIVSYAQ	Y	49.90	922.4760	8	1.6	462.2460	2	9.95	17	3928	15x.raw	1.8409E4	1	1	158	165			PEAKS DB
**total 1 peptides**																			

C7A770|C7A770_CORAV

back to list

| Protein Coverage | Supporting Peptides |

Protein Coverage:

1 TLAKVIYESI AYKFEASCFI PNIREQTKKH GLLYLQIQLI SQILGESETN IQINTCVATS MIANRIRQRR VLIILDDMDG
81 DEQVQALAGS HDWFGQGSRI IITSRDLHFL KIVLRDVGDV GDDTYEVELL NNEEALELFS WKAFKKPHPE KNYVELS**KDI**
161 **VSYAQ**

Supporting Peptides:

Peptide	Uniq	-10lgP	Mass	Length	ppm	m/z	z	RT	Fraction	Scan	Source File	Area Control RAW 15	#Feature	#Feature Control RAW 15	Start	End	PTM	AScore	Found By
S.KDIVSYAQ	Y	49.90	922.4760	8	1.6	462.2460	2	9.95	17	3928	15x.raw	1.8409E4	1	1	158	165			PEAKS DB
**total 1 peptides**																			

C7A765|C7A765_CORAV

back to list

| Protein Coverage | Supporting Peptides |

Protein Coverage:

1 TLAKVIYESI AYKFEASCFI PNIREQTKKH GLLYLQIQLI SQILSENETN IQINTCVATS MIANRIRQRR VLIILDDMDG
81 DEQVQALAGS HDWFGQGSRI IITSRDLHFL KIVLRDVGDV GDDTYLVELL NNEEALELFS WKAFKKPHPE KNYVELS**KDI**
161 **VSYAQ**

Supporting Peptides:

Peptide	Uniq	-10lgP	Mass	Length	ppm	m/z	z	RT	Fraction	Scan	Source File	Area Control RAW 15	#Feature	#Feature Control RAW 15	Start	End	PTM	AScore	Found By
**total 1 peptides**																			
**Peptide**	**Uniq**	**-10lgP**	**Mass**	**Length**	**ppm**	**m/z**	**z**	**RT**	**Fraction**	**Scan**	**Source File**	**Area Control RAW 15**	**#Feature**	**#Feature Control RAW 15**	**Start**	**End**	**PTM**	**AScore**	**Found By**
S.KDIVSYAQ	Y	49.90	922.4760	8	1.6	462.2460	2	9.95	17	3928	15x.raw	1.8409E4	1	1	158	165			PEAKS DB
**total 1 peptides**																			

Peptide List

Prepared with PEAKS ™ (bioinfor.com)

Summary

1. Notes

Spot 15 from RAW digested hazelnut

Cor a 8,cor a 9, cor a 11 & oleosin

2. Result Statistics

Figure 1. False discovery rate (FDR) curve. X axis is the number of peptides being kept. Y axis is the corresponding FDR.

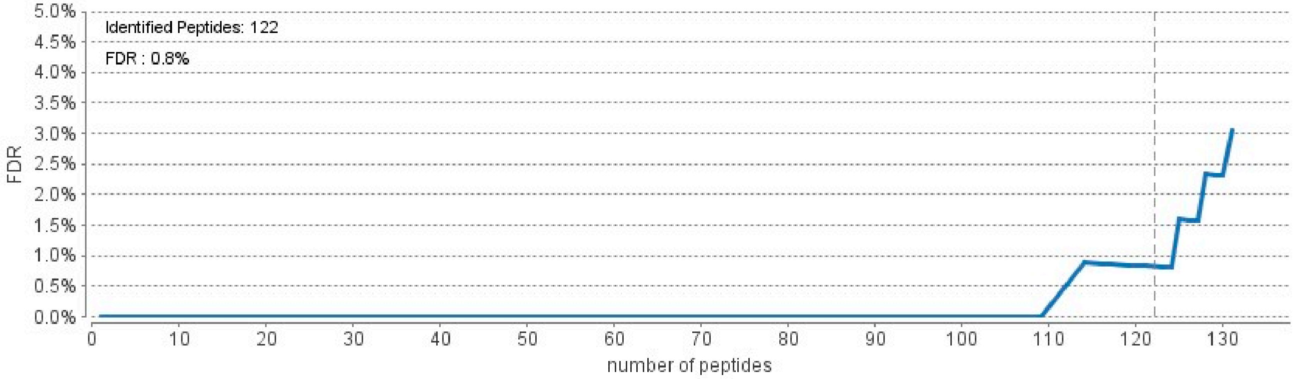

Figure 2. PSM score distribution. **(a)** Distribution of PEAKS peptide score; **(b)** Scatterplot of PEAKS peptide score versus precursor mass error.

(a) (b)

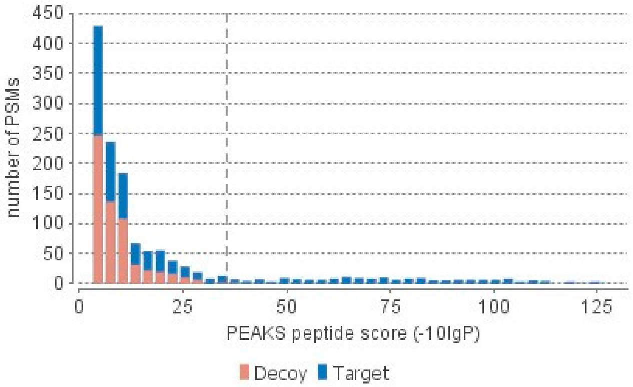

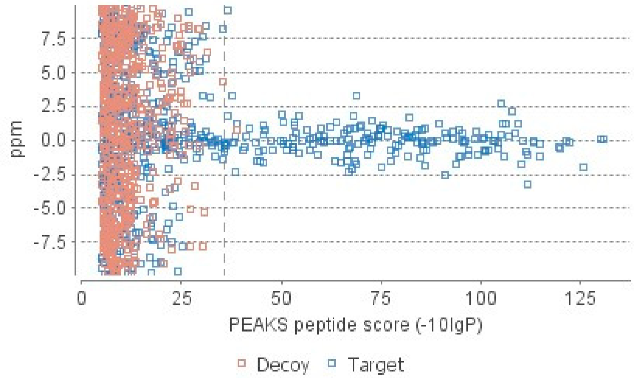

Table 1. Statistics of data.

	#Scans			#Features	Identified			#Peptides	#Sequences	#Proteins*		
	MS1	MS/MS	#Chimera		#PSMs	#Scans	#Features**			Groups	All	Top
Total	4024	4944	9	2954	186	186	102	122	116	5	5	5
Gastric RAW 15	4024	4944	9	2954	186	186	102	122	116	5	5	5

* proteins with significant peptides are used in counts.

** features are identified by DB search only.

Figure 3. Sample overlap for Proteins and Peptides (up to 8 samples). **(a)** All Proteins; **(b)** Top Proteins; **(c)** Peptides;

(a) **Not applicable to only one sample**

(b) **Not applicable to only one sample**

(c) **Not applicable to only one sample**

Figure 4. Distribution of peptide feature detection. **(a)** Feature m/z distribution; **(b)** Feature RT distribution.

(a) **(b)**

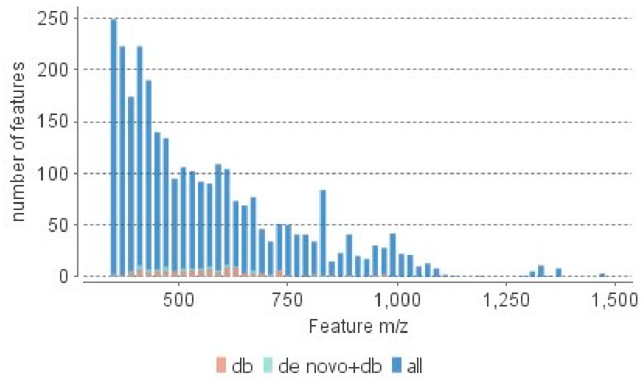

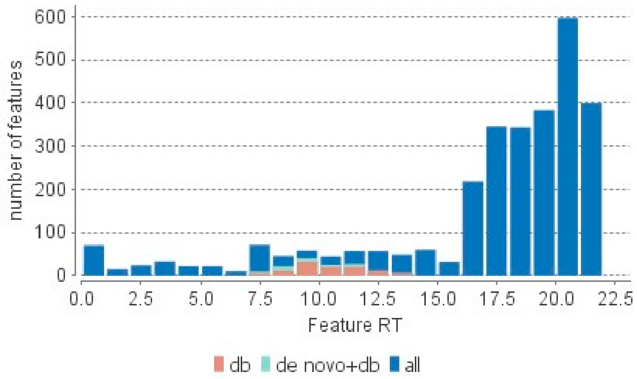

Figure 5. Distribution of identified peptide features. **(a)** Feature abundance distribution; **(b)** *De novo* sequencing validation.

(a)

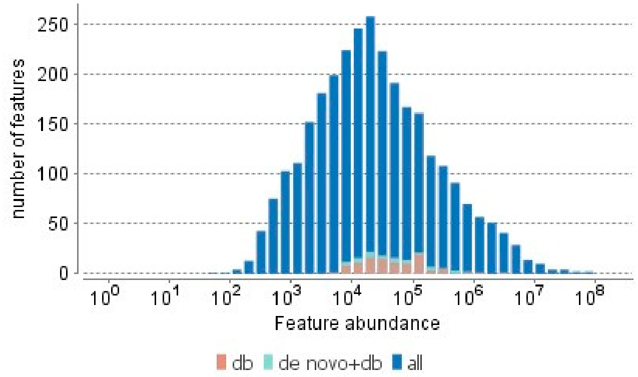

(b)

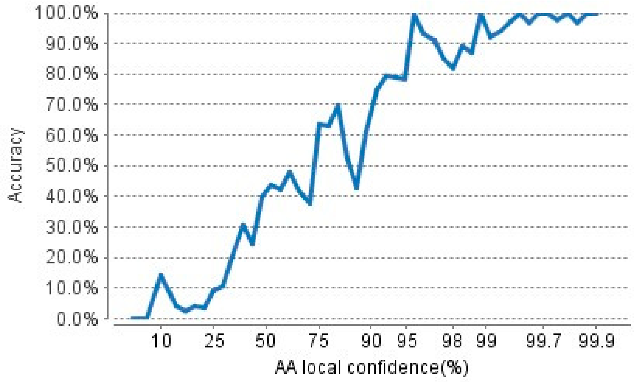

Table 2. Result filtration parameters.

Peptide -10lgP	≥35.7
PTM Ascore	≥20
Protein -10lgP	≥20
Proteins unique peptides	≥1
De novo score(%)	≥50%

Table 3. Statistics of filtered result.

FDR (Peptide-Spectrum Matches)	0.5%
FDR (Peptide Sequences)	0.8%
FDR (Protein Group)	20.0%
De Novo Only Spectra	112

3. Experiment Control

Table 4. PTM profile.

Name	ΔMass	Position	#PSM	-10lgP	Abundance	AScore
Oxidation	15.99	M	20	119.27	2.3E5	1000.00
Carbamidomethyl	57.02	C	7	108.46	1.23E4	1000.00

Figure 6. Precursor mass error of peptide-spectrum matches (PSM) in filtered result. **(a)** Distribution of precursor mass error in ppm; **(b)** Scatterplot of precursor m/z versus precursor mass error in ppm.

(a)

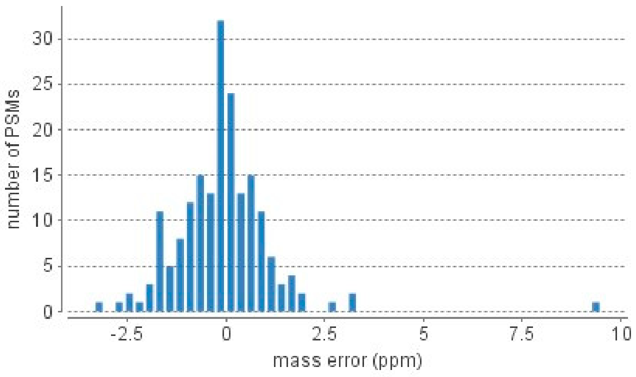

(b)

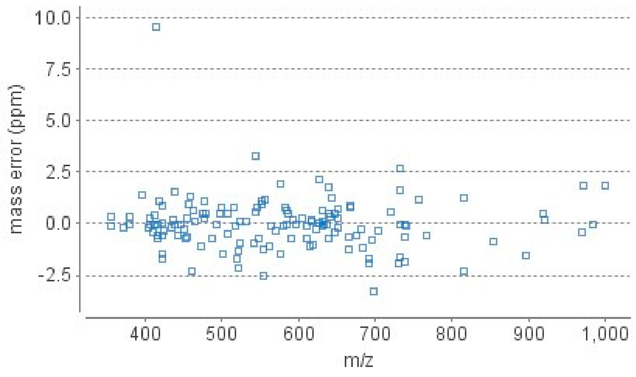

Table 5. Number of identified peptides in each sample by the number of missed cleavages.

Missed Cleavages	0	1	2	3	4+
Gastric RAW	15	99	22	1	0	0

4. Other Information

Table 6. Search parameters.

PEAKS Version: PEAKS Studio 10.6 build 20201015
Search Engine Name: PEAKS
Parent Mass Error Tolerance: 10.0 ppm
Fragment Mass Error Tolerance: 0.02 Da
Precursor Mass Search Type: monoisotopic
Enzyme: Trypsin
Max Missed Cleavages: 2
Digest Mode: Semispecific
Fixed Modifications:
Carbamidomethylation: 57.02
Variable Modifications:
Oxidation (M): 15.99
Deamidation (NQ): 0.98
Acetylation (K): 42.01
Acetylation (Protein N-term): 42.01
Acetylation (N-term): 42.01
Amidation: -0.98
Beta-methylthiolation: 45.99
Biotinylation: 226.08 and 303 more...
Max Variable PTM Per Peptide: 3
Database: Corylus aveliana iz 2020 od 156 KB
Taxon: All
Contaminant Database: crap

Table 7. Instrument parameters.

Fractions: 15y.raw
Ion Source: ESI(nano-spray)
Fragmentation Mode: CID, CAD(y and b ions)
MS Scan Mode: FT-ICR/Orbitrap
Searched Entry: 602
FDR Estimation: Enabled
De novo score(%) threshold: 15
Peptide hit threshold (-10logP): 30.0
Peaks run ID: 112
Merge Options: no merge
Precursor Options: corrected
Charge Options: no correction
Filter m/z: 200.0 - 2000.0
Filter Charge: 2 - 8
Process: true
Associate chimera: yes

MS/MS Scan Mode: FT-ICR/Orbitrap

Protein List

Protein Accession Contains:
Protein Description Contains:
Protein Sample Area >=
Protein PTM Contains:

Protein Group	Protein ID	Accession	-10lgP	Coverage (%)	Coverage (%) Gastric RAW 15	Area Gastric RAW 15	#Peptides	#Unique	#Spec Gastric RAW 15	PTM	Avg. Mass	Description
**6**	10069	sp|Q9ATH2|NLTP_CORAV	267.57	50	50	1.7989E6	7	7	15	Y	11806	Non-specific lipid-transfer protein Cor a 8 OS=Corylus avellana OX=13451 PE=1 SV=1
**9**	10065	A0A0A0P7E3|A0A0A0P7E3_CORAV	226.18	17	17	6.4707E3	9	1	9	N	58837	Cor a 9 allergen OS=Corylus avellana OX=13451 PE=2 SV=1
**8**	10066	Q8W1C2|Q8W1C2_CORAV	221.80	16	16	9.5282E5	9	1	9	Y	59127	11S globulin-like protein OS=Corylus avellana OX=13451 PE=2 SV=1
**13**	9619	Q8S4P9|Q8S4P9_CORAV	78.90	2	2	2.4963E4	1	1	1	N	50856	48-kDa glycoprotein OS=Corylus avellana OX=13451 PE=2 SV=1
**15**	10135	Q84T21|Q84T21_CORAV	38.43	5	5	0	1	1	1	Y	16698	Oleosin OS=Corylus avellana OX=13451 PE=2 SV=2
**total 5 proteins**												

sp|Q9ATH2|NLTP_CORAV

back to list

| Protein Coverage | Supporting Peptides |

Protein Coverage:

27 C 37 C

1 MGSLKLVCAV LLCMMVAAPV ARA**SLTCPQI KGNLTPCVLY LK**NGGVLPPS CCKGVRAVND ASRTTSDRQS ACNCLK**DTAK**

C Carbamidomethylation (+57.02)

97 C

81 **GIAGLNPNLA AGLPGKCGVN IPYKISPSTN CNNVK**

Supporting Peptides:

Peptide	Uniq	-10lgP	Mass	Length	ppm	m/z	z	RT	Fraction	Scan	Source File	Area Gastric RAW 15	#Feature	#Feature Gastric RAW 15	Start	End	PTM	AScore	Found By
Peptide	Uniq	-10lgP	Mass	Length	ppm	m/z	z	RT	Fraction	Scan	Source File	Area Gastric RAW 15	#Feature	#Feature Gastric RAW 15	Start	End	PTM	AScore	Found By
K.GIAGLNPNLAAGLPGK.C	Y	113.04	1461.8303	16	0.0	731.9224	2	11.88	18	4722	15y.raw	1.3108E6	1	1	81	96			PEAKS DB
K.DTAKGIAGLNPNLAAGLPGK.C	Y	110.35	1877.0370	20	0.0	626.6863	3	11.53	18	4573	15y.raw	8.1313E3	1	1	77	96			PEAKS DB
K.C(+57.02)GVNIPYK.I	Y	108.46	949.4691	8	0.5	475.7421	2	9.99	18	3962	15y.raw	1.233E4	1	1	97	104	Carbamidomethylation	C1:Carbamidomethylation:1000.00	PEAKS DB
K.GIAGLNPN(+.98)LAAGLPGK.C	Y	105.00	1462.8143	16	2.7	732.4164	2	12.07	18	4788	15y.raw	4.4999E4	1	1	81	96		N8:Deamidation (NQ):14.04	PEAKS DB
K.GNLTPC(+57.02)VLYLK.N	Y	104.99	1276.6849	11	0.5	639.3500	2	11.88	18	4699	15y.raw	1.1064E5	1	1	32	42	Carbamidomethylation	C6:Carbamidomethylation:1000.00	PEAKS DB
A.GLNPNLAAGLPGK.C	Y	87.93	1220.6876	13	-0.1	611.3510	2	10.95	18	4335	15y.raw	2.9769E4	1	1	84	96			PEAKS DB
A.SLTC(+57.02)PQIK.G	Y	66.61	945.4954	8	-1.1	473.7545	2	9.37	18	3699	15y.raw	1.2946E5	1	1	24	31	Carbamidomethylation	C4:Carbamidomethylation:1000.00	PEAKS DB
K.ISPSTNC(+57.02)NNVK	Y	57.82	1232.5819	11	0.2	617.2983	2	7.78	18	3090	15y.raw	1.546E4	1	1	105	115	Carbamidomethylation	C7:Carbamidomethylation:1000.00	PEAKS DB

K.GNLTP(+15.99)C(+57.02)VLYLK.N	Y	36.14	1292.6798	11	-0.4	647.3469	2	11.68	18	4614	15y.raw	1.3733E5	1	1	32	42	Carbamidomethylation	P5:Oxidation or Hydroxylation: 19.86;C6: Carbamidomethylation:1000.00	PEAKS PTM
**total 9 peptides**																			

A0A0A0P7E3|A0A0A0P7E3_CORAV

back to list

| Protein Coverage | Supporting Peptides |

Protein Coverage:

1 MAKLILVSFS LCLLVLFNGC LGIDVGLRRQ QQRHFGECNL DRLNALEPTN RIEAEAGQIE SWDHNDQQFQ CAGVAVIRRT

81 IEPNGLLLPQ YSNAPELIYI ERGRGITGVL FPGCPETFED PQQQSQQGQG QGQSQRSEQD RHQKIRYFQE GDIIALPAGV

161 AHWCYNDGDS PVVAVSLLHT NNYANQLDEN PRHFYLAGNP DDEHQRQGQQ QFGQRRRQQQ HSRGKEGEQE QQGEGNNVFS

241 GFDAEF**LADA FNVDVDTAR**R LQSNQDKRR**N IVKVEGR**LQM VRPERSRQEW ERQERQERES EQERERQRRQ GGRGRDVNGF

321 EETICSLRLM ENIGSRSR**AD IYTEQVGRIN TVNSNTLPVL R**WLQLSAERG DLQREGLYVP HWNLNAHSVV YAIRGR**ARVQ**

401 **VVDDNGNTVF** DDELRQGQVL TIPQNFAVAK RAESEGFEWV AFKTNDNAQI SPLAGRTSAI R**ALPDDVLAN AFQISREEAR**

481 RLK**YNRQETT LAR**SSRSSSE RMRRRSESEG RAEA

Supporting Peptides:

Peptide	Uniq	-10lgP	Mass	Length	ppm	m/z	z	RT	Fraction	Scan	Source File	Area Gastric RAW 15	#Feature	#Feature Gastric RAW 15	Start	End	PTM	AScore	Found By
R.INTVNSNTLPVLR.W	N	90.95	1439.8096	13	0.6	720.9125	2	11.21	18	4449	15y.raw	1.6311E4	1	1	349	361			PEAKS DB
L.ANAFQISR.E	N	81.73	905.4719	8	0.2	453.7433	2	9.82	18	3888	15y.raw	3.1768E4	1	1	469	476			PEAKS DB
F.LADAFNVDVDTAR.R	N	78.66	1405.6837	13	-0.4	703.8489	2	11.39	18	4512	15y.raw	0	0	0	247	259			PEAKS DB
R.ARVQVVDDNGNTVF.D	N	72.36	1532.7583	14	-0.6	767.3860	2	11.02	18	4365	15y.raw	1.852E4	1	1	397	410			PEAKS DB
K.YNRQETTLAR.S	Y	70.13	1250.6367	10	1.1	417.8866	3	7.74	18	3066	15y.raw	6.4707E3	1	1	484	493			PEAKS DB
R.ALPDDVLANAFQISR.E	N	66.69	1628.8522	15	-2.3	815.4315	2	13.43	18	5316	15y.raw	9.7378E3	1	1	462	476			PEAKS DB
R.ADIYTEQVGR.I	N	66.51	1150.5618	10	-1.5	576.2873	2	9.70	18	3841	15y.raw	0	0	0	339	348			PEAKS DB

R.NIVKVEGR.L	N	50.51	913.5345	8	1.3	457.7751	2	7.91	18	3129	15y.raw	0	0	0	270	277			PEAKS DB
A.FQISREEAR.R	N	49.70	1134.5781	9	0.0	379.2000	3	8.64	18	3413	15y.raw	1.319E5	1	1	472	480			PEAKS DB
**total 9 peptides**																			

Q8W1C2|Q8W1C2_CORAV

back to list

| Protein Coverage | Supporting Peptides |

Protein Coverage:

P Pyro-glu from Q (-17.03)

```
  1 MAKLILVSFS LCLLVLFNGC LGINVGLRRQ QQRYFGECNL DRLNALEPTN RIEAEACQIE SWDHNDQQFQ CAGVAVIRRT
 81 IEPNGLLLPQ YSNAPELIYI ERGRGITGVL FPGCPETFED PQQQSQQGQR QGQGQSQRSE QDRHQKIRHF REGDIIALPA
161 GVAHWCYNDG DSPVVTVSLL HTNNYANQLD ENPRHFYLAG NPDDEHQRQG QQQFGQRRRQ QQHSHGEQGE QEQQGEGNNV
241 FSGFDAEFLA DAFNVDVDTA RRLQSNQDKR RNIVKVEGRL QVVRPERSRQ EWERQERQER ESEQERERQR RQGGRGRDVN
321 GFEETICSLR LRENICTRSR ADIYTEQVGR INTVNSNTLP VLRWLQLSAE RGDLQREGLY VPHWNLNAHS VVYAIRGRAR
401 VQVVDDNGNT VFDDELRQGQ VLTIPQNFAV AKRAESEGFE WVAFKTNDNA QISPLAGRTS AIRALPDDVL ANAFQISREE
481 ARRLKYNRQE TTLVRSSRSS SERKRRSESE GRAEA
```

Supporting Peptides:

Peptide	Uniq	-10lgP	Mass	Length	ppm	m/z	z	RT	Fraction	Scan	Source File	Area Gastric RAW 15	#Feature	#Feature Gastric RAW 15	Start	End	PTM	AScore	Found By
R.INTVNSNTLPVLR.W	N	90.95	1439.8096	13	0.6	720.9125	2	11.21	18	4449	15y.raw	1.6311E4	1	1	351	363			PEAKS DB
**total 9 peptides**																			

Peptide	Uniq	-10lgP	Mass	Length	ppm	m/z	z	RT	Fraction	Scan	Source File	Area Gastric RAW 15	#Feature	#Feature Gastric RAW 15	Start	End	PTM	AScore	Found By
L.ANAFQISR.E	N	81.73	905.4719	8	0.2	453.7433	2	9.82	18	3888	15y.raw	3.1768E4	1	1	471	478			PEAKS DB
F.LADAFNVDVDTAR.R	N	78.66	1405.6837	13	-0.4	703.8489	2	11.39	18	4512	15y.raw	0	0	0	249	261			PEAKS DB
R.ARVQVVDDNGNTVF.D	N	72.36	1532.7583	14	-0.6	767.3860	2	11.02	18	4365	15y.raw	1.852E4	1	1	399	412			PEAKS DB
R.ALPDDVLANAFQISR.E	N	66.69	1628.8522	15	-2.3	815.4315	2	13.43	18	5316	15y.raw	9.7378E3	1	1	464	478			PEAKS DB
R.ADIYTEQVGR.I	N	66.51	1150.5618	10	-1.5	576.2873	2	9.70	18	3841	15y.raw	0	0	0	341	350			PEAKS DB

R.NIVKVEGR.L	N	50.51	913.5345	8	1.3	457.7751	2	7.91	18	3129	15y.raw	0	0	0	272	279			PEAKS DB
A.FQISREEAR.R	N	49.70	1134.5781	9	0.0	379.2000	3	8.64	18	3413	15y.raw	1.319E5	1	1	474	482			PEAKS DB
L.Q(-17.03)VVRPER.S	Y	38.22	865.4770	7	-0.2	433.7457	2	8.55	18	3385	15y.raw	9.5282E5	1	1	281	287	Pyro-glu from Q	Q1:Pyro-glu from Q:1000.00	PEAKS PTM
**total 9 peptides**																			

Q8S4P9|Q8S4P9_CORAV

back to list

| Protein Coverage | Supporting Peptides |

Protein Coverage:

```
  1 MLPKEDPELK KCKHKCRDER QFDEQQRRDG KQICEEKARE RQQEEGNSSE ESYGKEQEEN PYVFQDEHFE SRVKTEEGRV
 81 QVLENFTKRS RLLSGIENFR LAILEANPHT FISPAHFDAE LVLFVAKGRA TITMVREEKR ESFNVEHGDI IRIPAGTPVY
161 MINRDENEKL FIVKILQPVS APGHFEAFYG AGGEDPESFY RAFSWEVLEA ALKVRREQLE KVFGEQSKGS IVKASREKIR
241 ALSQHEEGPP RIWPFGGESS GPINLLHKHP SQSNQFGRLY EAHPDDHKQL QDLDLMVSFA NITKGSMAGP YYNSRATKIS
321 VVVEGEGFFE MACPHLSSSS GSYQKISARL RRGVVFVAPA GHPVAVIASQ NNNLQVLCFE VNAHGNSRFP LAGKGNIVNE
401 FERDAKELAF NLPSREVERI FKNQDQAFFF PGPNKQQEEG GRGGRAFE
```

Supporting Peptides:

Peptide	Uniq	-10lgP	Mass	Length	ppm	m/z	z	RT	Fraction	Scan	Source File	Area Gastric RAW 15	#Feature	#Feature Gastric RAW 15	Start	End	PTM	AScore	Found By
K.ILQPVSAPGHF.E	Y	78.90	1164.6292	11	0.6	583.3222	2	11.17	18	4423	15y.raw	2.4963E4	1	1	175	185			PEAKS DB
**total 1 peptides**																			

Q84T21|Q84T21_CORAV

back to list

| Protein Coverage | Supporting Peptides |

Protein Coverage:

```
  1 MADRPQQLQV HPQRGHGHYE GGIKNQRGGG PSAVKVMAVL AALPVGGTLL ALAGLTLAGS VIGLLVTSPL FIIFSPVLVP
 81 AAIVVGLAVA SFLSSGALGL TGLSSLSWVL NYLRCASQSL PREMDQAKRR MQDMAAFVGQ KTREVGQEIQ SRAQEGRRT
```

C Carbamidomethylation (+57.02)

Supporting Peptides:

Peptide	Uniq	-10lgP	Mass	Length	ppm	m/z	z	RT	Fraction	Scan	Source File	Area Gastric RAW 15	#Feature	#Feature Gastric RAW 15	Start	End	PTM	AScore	Found By
Peptide	Uniq	-10lgP	Mass	Length	ppm	m/z	z	RT	Fraction	Scan	Source File	Area Gastric RAW 15	#Feature	#Feature Gastric RAW 15	Start	End	PTM	AScore	Found By
R.C(+57.02)ASQSLPR.E	Y	38.43	917.4389	8	-2.3	459.7257	2	8.25	18	3263	15y.raw	0	0	0	115	122	Carbamidomethylation	C1:Carbamidomethylation:1000.00	PEAKS DB

total 1 peptides

Peptide List

Prepared with PEAKS ™ (bioinfor.com)

Summary

1. Notes

Spot 15 from roasted digested hazelnut

2. Result Statistics

Figure 1. False discovery rate (FDR) curve. X axis is the number of peptides being kept. Y axis is the corresponding FDR.

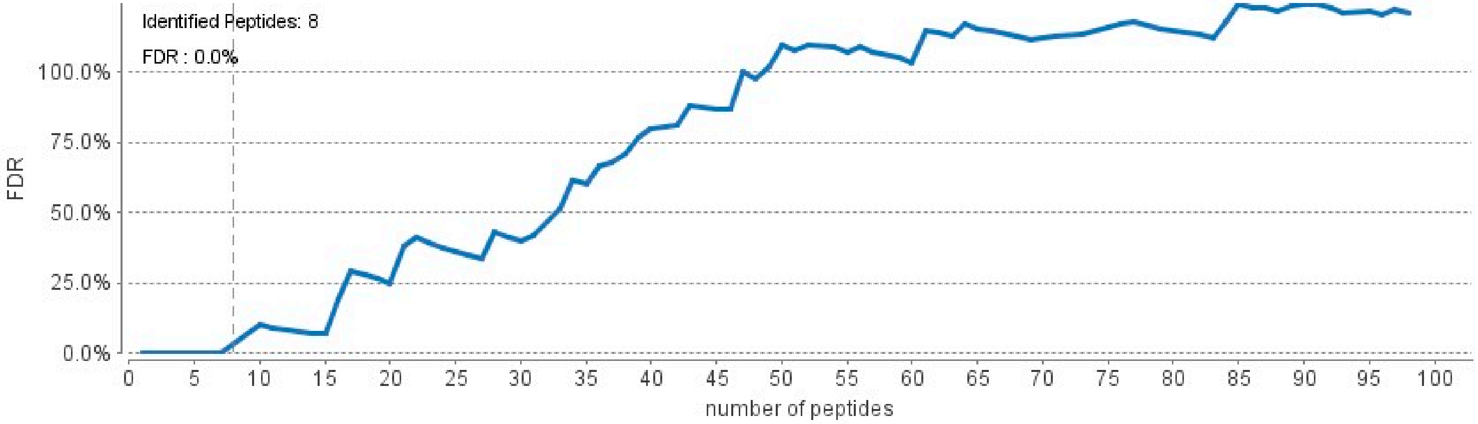

Figure 2. PSM score distribution. **(a)** Distribution of PEAKS peptide score; **(b)** Scatterplot of PEAKS peptide score versus precursor mass error.

(a) **(b)**

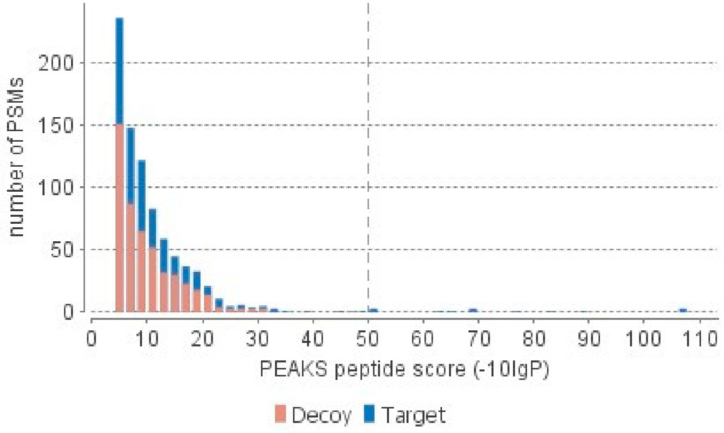

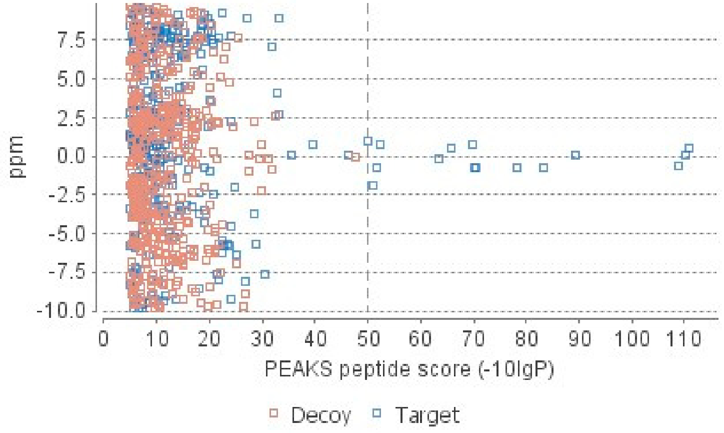

Table 1. Statistics of data.

	#Scans			#Features	Identified			#Peptides	#Sequences	#Proteins*		
	MS1	MS/MS	#Chimera		#PSMs	#Scans	#Features**			Groups	All	Top
Total	4721	4211	5	2403	14	14	5	8	8	1	1	1
Gastric RAOSTED 13	4721	4211	5	2403	14	14	5	8	8	1	1	1

* proteins with significant peptides are used in counts.

** features are identified by DB search only.

Figure 3. Sample overlap for Proteins and Peptides (up to 8 samples). **(a)** All Proteins; **(b)** Top Proteins; **(c)** Peptides;

(a) **Not applicable to only one sample**

(b) **Not applicable to only one sample**

(c) **Not applicable to only one sample**

Figure 4. Distribution of peptide feature detection. **(a)** Feature m/z distribution; **(b)** Feature RT distribution.

(a) (b)

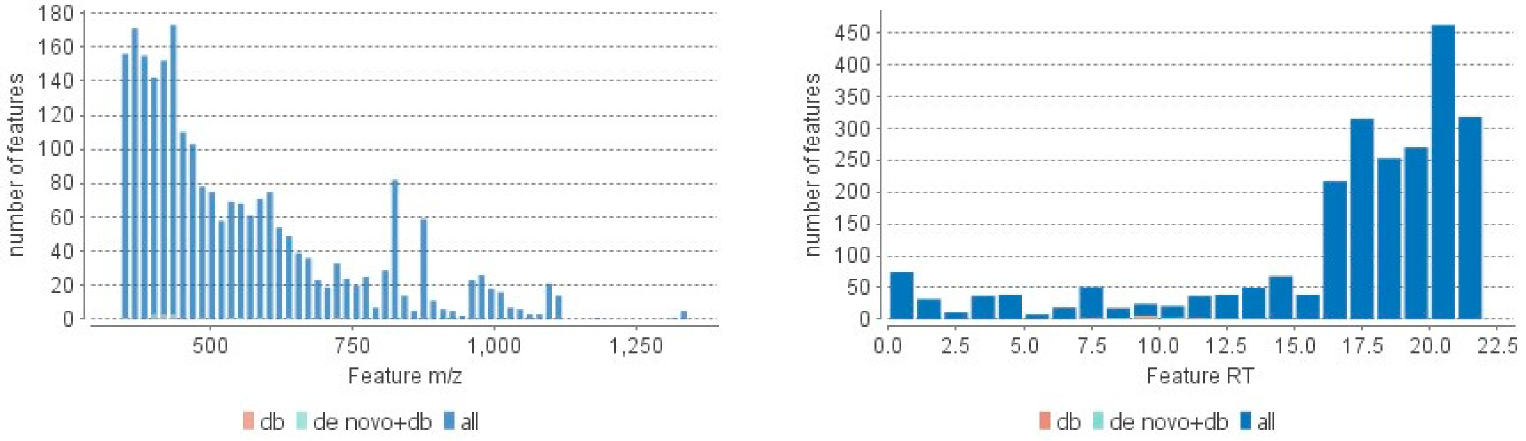

Figure 5. Distribution of identified peptide features. **(a)** Feature abundance distribution; **(b)** *De novo* sequencing validation.

(a) (b)

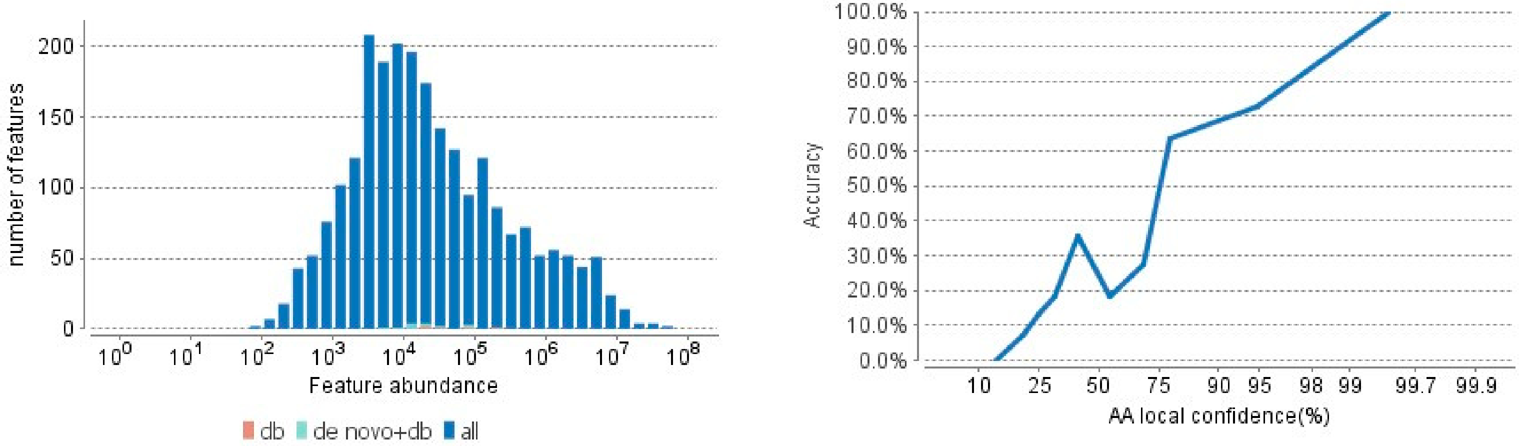

Table 2. Result filtration parameters.

Peptide -10lgP	≥50.1
PTM Ascore	≥20

Protein -10lgP	≥20
Proteins unique peptides	≥1
De novo score(%)	≥50%

Table 3. Statistics of filtered result.

Table 4. PTM profile.

FDR (Peptide-Spectrum Matches)	0.0%
FDR (Peptide Sequences)	0.0%
FDR (Protein Group)	0.0%
De Novo Only Spectra	92

3. Experiment Control

Figure 6. Precursor mass error of peptide-spectrum matches (PSM) in filtered result. **(a)** Distribution of precursor mass error in ppm; **(b)** Scatterplot of precursor m/z versus precursor mass error in ppm.

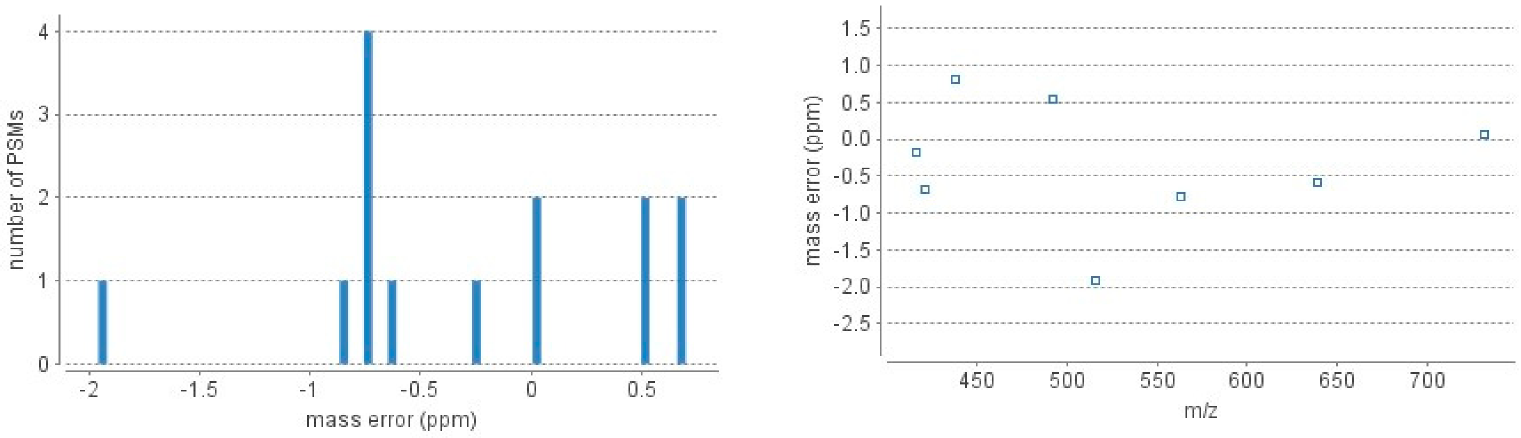

(a) **(b)**

Table 5. Number of identified peptides in each sample by the number of missed cleavages.

**Name**	**ΔMass** **Position**
	**#PSM** **-10lgP**
	**Abundance** **AScore**

proteins

Missed Cleavages	0	1	2	3	4+
Gastric RAOSTED 13	6	2	0	0	0

4. Other Information

Table 6. Search parameters.

PEAKS Version: PEAKS Studio 10.6 build 20201015

Search Engine Name: PEAKS

Parent Mass Error Tolerance: 10.0 ppm

Table 7. Instrument parameters.

Fractions: 13d.raw
Ion Source: ESI(nano-spray)
Fragmentation Mode: CID, CAD(y and b ions) Fragment Mass Error Tolerance: 0.02 Da
Precursor Mass Search Type: monoisotopic
Enzyme: Trypsin
Max Missed Cleavages: 2
Digest Mode: Semispecific Fixed Modifications: Carbamidomethylation: 57.02 Variable

Database: Corylus aveliana iz 2020 od 156 KB
Taxon: All
Contaminant Database: crap
Searched Entry: 602
FDR Estimation: Enabled
De novo score(%) threshold: 15
Peptide hit threshold (-10logP): 30.0
Peaks run ID: 119
Merge Options: no merge
Precursor Options: corrected
Charge Options: no correction
Filter m/z: 200.0 - 2000.0
Filter Charge: 2 - 8
Process: true
Associate chimera: yes

Protein List

Protein Group	Protein ID	Accession	-10lgP	Coverage (%)	Coverage (%) Gastric RAOSTED 13	Area Gastric RAOSTED 13	#Peptides	#Unique	#Spec Gastric RAOSTED 13	PTM	Avg. Mass	Description
**total 1 proteins**												

Modifications: Oxidation (M): 15.99
Deamidation (NQ): 0.98
Acetylation (K): 42.01
Acetylation (Protein N-term): 42.01
Acetylation (N-term): 42.01
Amidation: -0.98
Beta-methylthiolation: 45.99 Biotinylation: 226.08 and 303 more... Max Variable PTM Per Peptide: 3

Protein Accession Contains:
Protein Description Contains:
Protein Sample Area >= Protein PTM Contains:
MS Scan Mode: FT-ICR/Orbitrap MS/MS Scan Mode: FT-ICR/Orbitrap

Protein Group	Protein ID	Accession	-10lgP	Coverage (%)	Coverage (%) Gastric RAOSTED 13	Area Gastric RAOSTED 13	#Peptides	#Unique	#Spec Gastric RAOSTED 13	PTM	Avg. Mass	Description
**4**	10069	sp|Q9ATH2|NLTP_CORAV	135.37	14	14	9.3628E4	1	1	2	N	11806	Non-specific lipid-transfer protein Cor a 8 OS=Corylus avellana OX=13451 PE=1 SV=1
**total 1 proteins**												

sp|Q9ATH2|NLTP_CORAV

back to list

| Protein Coverage | Supporting Peptides | Protein

Coverage:

```
 1 MGSLKLVCAV LLCMMVAAPV ARASLTCPQI KGNLTPCVLY LKNGGVLPPS CCKGVRAVND ASRTTSDRQS ACNCLKDTAK
81 GIAGLNPNLA AGLPGKCGVN IPYKISPSTN CNNVK
```

Supporting Peptides:

Peptide	Uniq	-10lgP	Mass	Length	ppm	m/z	z	RT	Fraction	Scan	Source File	Area Gastric RAOSTED 13	#Feature	#Feature Gastric RAOSTED 13	Start	End	PTM	AScore	Found By
K.GIAGLNPNLAAGLPGK.C	Y	110.33	1461.8303	16	0.1	731.9225	2	11.85	11	4660	13d.raw	9.3628E4	1	1	81	96			PEAKS DB
**total 1 peptides**																			

Peptide List

Prepared with PEAKS ™ (bioinfor.com)
